# Supplementary material for: A Systematic Review with a Meta-Analysis of the Motivational Climate and Hedonic Well-Being Constructs: The Importance of the Athlete Level
Source: Eur J Investig Health Psychol Educ. 2024 Apr 9;14(4):976–1001. doi: 10.3390/ejihpe14040064 (PMC11048888; doi:10.3390/ejihpe14040064)
Supplement: Supplementary file 1 [file ejihpe-14-00064-s001.zip › ejihpe-2919154-supplementary.pdf]

Supplemental Tables and Figures

Contents:

- **Supplemental Table 1.** PRISMA checklist.
- **Supplemental Table 2.** Reviewed study references.
- **Supplement Table 3.** Correlates entered for each study.
- **Supplement Figures.** Remove-one study figures.
- **Supplement Figures.** Cumulative analysis by year figures.

Supplemental Table 1. PRISMA checklist.

Section and Topic	Item #	Checklist item	Location where item is reported
**TITLE**			
Title	1	Identify the report as a systematic review.	Title
**ABSTRACT**			
Abstract	2	See the PRISMA 2020 for Abstracts checklist.	Yes, within word limits.
**INTRODUCTION**			
Rationale	3	Describe the rationale for the review in the context of existing knowledge.	Introduction
Objectives	4	Provide an explicit statement of the objective(s) or question(s) the review addresses.	1.1.
**METHODS**			
Eligibility criteria	5	Specify the inclusion and exclusion criteria for the review and how studies were grouped for the syntheses.	2.1.
Information sources	6	Specify all databases, registers, websites, organisations, reference lists and other sources searched or consulted to identify studies. Specify the date when each source was last searched or consulted.	2.2.
Search strategy	7	Present the full search strategies for all databases, registers and websites, including any filters and limits used.	2.2., supplement table, fig.1
Selection process	8	Specify the methods used to decide whether a study met the inclusion criteria of the review, including how many reviewers screened each record and each report retrieved, whether they worked independently, and if applicable, details of automation tools used in the process.	2.1.
Data collection process	9	Specify the methods used to collect data from reports, including how many reviewers collected data from each report, whether they worked independently, any processes for obtaining or confirming data from study investigators, and if applicable, details of automation tools used in the process.	2.3.
Data items	10a	List and define all outcomes for which data were sought. Specify whether all results that were compatible with each outcome domain in each study were sought (e.g. for all measures, time points, analyses), and if not, the methods used to decide which results to collect.	2.3.
	10b	List and define all other variables for which data were sought (e.g. participant and intervention characteristics, funding sources). Describe any assumptions made about any missing or unclear information.	2.3.
Study risk of bias assessment	11	Specify the methods used to assess risk of bias in the included studies, including details of the tool(s) used, how many reviewers assessed each study and whether they worked independently, and if applicable, details of automation tools used in the process.	2.4.
Effect measures	12	Specify for each outcome the effect measure(s) (e.g. risk ratio, mean difference) used in the synthesis or presentation of results.	2.5.
Synthesis	13a	Describe the processes used to decide which studies were eligible for each synthesis (e.g. tabulating the study	2.5.

Section and Topic	Item #	Checklist item	Location where item is reported
methods		intervention characteristics and comparing against the planned groups for each synthesis (item #5)).	
	13b	Describe any methods required to prepare the data for presentation or synthesis, such as handling of missing summary statistics, or data conversions.	2.5.
	13c	Describe any methods used to tabulate or visually display results of individual studies and syntheses.	2.5.
	13d	Describe any methods used to synthesize results and provide a rationale for the choice(s). If meta-analysis was performed, describe the model(s), method(s) to identify the presence and extent of statistical heterogeneity, and software package(s) used.	2.5.
	13e	Describe any methods used to explore possible causes of heterogeneity among study results (e.g. subgroup analysis, meta-regression).	2.5.
	13f	Describe any sensitivity analyses conducted to assess robustness of the synthesized results.	2.5.
Reporting bias assessment	14	Describe any methods used to assess risk of bias due to missing results in a synthesis (arising from reporting biases).	2.5.
Certainty assessment	15	Describe any methods used to assess certainty (or confidence) in the body of evidence for an outcome.	2.5.
**RESULTS**			
Study selection	16a	Describe the results of the search and selection process, from the number of records identified in the search to the number of studies included in the review, ideally using a flow diagram.	3.1.
	16b	Cite studies that might appear to meet the inclusion criteria, but which were excluded, and explain why they were excluded.	3.1.
Study characteristics	17	Cite each included study and present its characteristics.	3.1.
Risk of bias in studies	18	Present assessments of risk of bias for each included study.	3.2.
Results of individual studies	19	For all outcomes, present, for each study: (a) summary statistics for each group (where appropriate) and (b) an effect estimate and its precision (e.g. confidence/credible interval), ideally using structured tables or plots.	3.2., 3.3
Results of syntheses	20a	For each synthesis, briefly summarise the characteristics and risk of bias among contributing studies.	3.2., 3.3
	20b	Present results of all statistical syntheses conducted. If meta-analysis was done, present for each the summary estimate and its precision (e.g. confidence/credible interval) and measures of statistical heterogeneity. If comparing groups, describe the direction of the effect.	3.2., 3.3, 3.5
	20c	Present results of all investigations of possible causes of heterogeneity among study results.	3.2., 3.3,
	20d	Present results of all sensitivity analyses conducted to assess the robustness of the synthesized results.	3.2., 3.3., 3.4

Section and Topic	Item #	Checklist item	Location where item is reported
Reporting biases	21	Present assessments of risk of bias due to missing results (arising from reporting biases) for each synthesis assessed.	3.2., 3.3
Certainty of evidence	22	Present assessments of certainty (or confidence) in the body of evidence for each outcome assessed.	Discussion
**DISCUSSION**			
Discussion	23a	Provide a general interpretation of the results in the context of other evidence.	Discussion, 4.1.
	23b	Discuss any limitations of the evidence included in the review.	4.2.
	23c	Discuss any limitations of the review processes used.	4.2
	23d	Discuss implications of the results for practice, policy, and future research.	4.2, Conclusions
**OTHER INFORMATION**			
Registration and protocol	24a	Provide registration information for the review, including register name and registration number, or state that the review was not registered.	Abstract, 2.
	24b	Indicate where the review protocol can be accessed, or state that a protocol was not prepared.	Described in the methods
	24c	Describe and explain any amendments to information provided at registration or in the protocol.	Not needed
Support	25	Describe sources of financial or non-financial support for the review, and the role of the funders or sponsors in the review.	Reported in funding statement.
Competing interests	26	Declare any competing interests of review authors.	No competing interests
Availability of data, code and other materials	27	Report which of the following are publicly available and where they can be found: template data collection forms; data extracted from included studies; data used for all analyses; analytic code; any other materials used in the review.	Supplement files and manuscript tables

Supplemental Table 2. Reviewed study references.

Citation (sorted A to Z)
2013 NASPSPA Conference abstract collection: Motor learning and control; Developmental perspectives: Motor control/coordination/rehabilitation; Sport and exercise psychology. (2013). Journal of Sport & Exercise Psychology, 35(Suppl), 16–122.
Abatzoglou, G., Kalogiannis, P., Sagovits, A., & Papaioannou, A. (2009). The Influence of Physical Education Teacher, Mother, and Best Friend, in Goal Achievement, Satisfaction in Physical Education and Exercise Participation of the Students that are not Athletes. Inquiries in Sport & Physical Education, 7(3), 265–278.
Abós Catalán, Á., Sevil Serrano, J., Martín-Albo Lucas, J., Julián Clemente, J. A., & García-González, L. (2018). An integrative framework to validate the Need-Supportive Teaching Style Scale (NSTSS) in secondary teachers through exploratory structural equation modeling. Contemporary Educational Psychology, 52, 48–60. https://doi.org/10.1016/j.cedpsych.2018.01.001
Abrahamsen, F. E., & Kristiansen, E. (2015). The Dark Side of a Mastery Emphasis: Can Mastery Involvement Create Stress and Anxiety? International Journal of Applied Sports Sciences, 27(2), 76–86. https://doi.org/10.24985/ijass.2015.27.2.76
Abrahamsen, F. E., & Pensgaard, A. M. (2012). Longitudinal Changes in Motivational Climate and Performance Anxiety Among Elite Handball Players. International Journal of Applied Sports Sciences, 24(1), 31–42. https://doi.org/10.24985/ijass.2012.24.1.31
Abrahamsen, F. E., Roberts, G. C., & Pensgaard, A. M. (2008). Achievement goals and gender effects on multidimensional anxiety in national elite sport. Psychology of Sport & Exercise, 9(4), 449–464.
Abrahamsen, F. E., Roberts, G. C., Pensgaard, A. M., & Ronglan, L. T. (2008). Perceived ability and social support as mediators of achievement motivation and performance anxiety. Scandinavian Journal of Medicine & Science in Sports, 18(6), 810–821.
Abraldes, J. A., Granero-Gallegos, A., & Baena-Extremera, A. (2016). Orientaciones de meta, satisfacción, creencias de éxito y clima motivacional en nadadores / Goal Orientations, Satisfaction, Beliefs in Sport Success and Motivational Climate in Swimmers. Revista Internacional de Medicina y Ciencias de La Actividad Física y Del Deporte, 63(2016). https://doi.org/10.15366/rimcafd2016.63.011

Ahmadi, M., Mehdi Namazizadeh, M., & Mokhtari, P. (2012). Perceived motivational climate, basic psychological needs and self-determined motivation in youth male athletes. World Applied Sciences Journal 16 (9): 1189-1195.

Al-Yaaribi, A., & Kavussanu, M. (2018). Consequences of prosocial and antisocial behaviors in adolescent male soccer players: The moderating role of motivational climate. Psychology of Sport and Exercise, 37, 91–99. https://doi.org/10.1016/j.psychsport.2018.04.005

Alesi, M., Gómez-López, M., Chicau Borrego, C., Monteiro, D., & Granero-Gallegos, A. (2019). Effects of a Motivational Climate on Psychological Needs Satisfaction, Motivation and Commitment in Teen Handball Players. International Journal of Environmental Research and Public Health, 16(15), 2702. https://doi.org/10.3390/ijerph16152702

Alferman et al. 2013

Alfermann, D., Geisler, G., & Okade, Y. (2013). Goal orientation, evaluative fear, and perceived coach behavior among competitive youth swimmers in Germany and Japan. Psychology of Sport and Exercise, 14(3), 307–315. https://doi.org/10.1016/j.psychsport.2012.11.005

Alfermann, D., Geisler, G., & Okade, Y. (2013). Goal orientation, evaluative fear, and perceived coach behavior among competitive youth swimmers in Germany and Japan. Psychology of Sport and Exercise, 14(3), 307–315. https://doi.org/10.1016/j.psychsport.2012.11.005

Allen, J. B., & Hodge, K. (2006). Fostering a Learning Environment: Coaches and the Motivational Climate. International Journal of Sports Science & Coaching, 1(3), 261–277. https://doi.org/10.1260/174795406778604564

Allen, J. B., & Shaw, S. (2009). “Everyone rolls up their sleeves and mucks in”: Exploring volunteers’ motivation and experiences of the motivational climate of a sporting event. Sport Management Review, 12(2), 79–90.

Almagro, B. J., & Conde, C. (2012). Factores motivacionales como predictores de la intención de ser físicamente activos en jóvenes jugadores de baloncesto. / Motivational factors as predictors of young basketball players´ intention to be physically active. Cuadernos de Psicología Del Deporte, 12(S1), 1–4.

Almagro, B. J., Sáenz-López, P., González-Cutre, D., & Moreno-Murcia, J. A. (2011). Clima motivacional percibido, necesidades psicológicas y motivación intrínseca como predictores del compromiso deportivo en adolescentes. (Perceived motivational climate, psychological needs and intrinsic motivation as predictors of sport commitment in adolescent athletes). RICYDE. Revista Internacional de Ciencias Del Deporte, 7(24), 250–265. https://doi.org/10.5232/ricyde2011.02501

Almagro, B. J., Sáenz-López, P., González-Cutre, D., & Moreno-Murcia, J. A. (2011). Clima motivacional percibido, necesidades psicológicas y motivación intrínseca como predictores del compromiso deportivo en adolescentes. (Perceived motivational climate, psychological needs and intrinsic motivation as predictors of sport commitment in adolescent athletes). RICYDE. Revista Internacional de Ciencias Del Deporte, 7(24), 250–265. https://doi.org/10.5232/ricyde2011.02501
Almagro, Bartolomé J.; Sáenz-López, Pedro; Moreno-Murcia, Juan Antonio Perfiles motivacionales de deportistas adolescentes españoles Revista de Psicología del Deporte, vol. 21, núm. 2, 2012, pp. 223-231
Almolda-Tomás, F. J., Sevil-Serrano, J., Julián-Clemente, J. A., Abarca-Sos, A., Aibar-Solana, A., & García-González, L. (2014). Application of teaching strategies for improving students' situational motivation in physical education. Electronic Journal of Research in Educational Psychology, 12(2), 391–418.
Alvarez, M. S., Balaguer, I., Castillo, I., & Duda, J. L. (2012). The Coach-Created Motivational Climate, Young Athletes' Well-Being, and Intentions to Continue Participation. Journal of Clinical Sport Psychology, 6(2), 166–179. https://doi.org/10.1123/jcsp.6.2.166
Amaro, N., Monteiro, D., Rodrigues, F., Matos, R., Jacinto, M., Cavaco, B., Jorge, S., & Antunes, R. (2023). Task-Involving Motivational Climate and Enjoyment in Youth Male Football Athletes: The Mediation Role of Self-Determined Motivation. International Journal of Environmental Research and Public Health, 20(4), 3044. https://doi.org/10.3390/ijerph20043044
Appleton, P. R., & Duda, J. L. (2016). Examining the interactive effects of coach-created empowering and disempowering climate dimensions on athletes' health and functioning. Psychology of Sport and Exercise, 26, 61–70. https://doi.org/10.1016/j.psychsport.2016.06.007
Appleton, P., Hall, H., & Hill, A. (2011). Examining the influence of the parent-initiated and coach-created motivational climates upon athletes' perfectionistic cognitions. Journal of Sports Sciences, 29(7), 661–671.
Atkins, M. R., Johnson, D. M., Force, E. C., & Petrie, T. A. (2013). "Do I Still Want to Play?" Parents' and Peers' Influences on Girls' Continuation in Sport. Journal of Sport Behavior, 36(4), 329–345.
Atkins, M. R., Johnson, D. M., Force, E. C., & Petrie, T. A. (2015). Peers, parents, and coaches, oh my! The relation of the motivational climate to boys' intention to continue in sport. Psychology of Sport and Exercise, 16, 170–180. https://doi.org/10.1016/j.psychsport.2014.10.008

Baena-Extremera, A., Gómez-López, M., Granero-Gallegos, A., & Martínez-Molina, M. (2016). Modelo de predicción de la satisfacción y diversión en Educación Física a partir de la autonomía y el clima motivacional = Prediction model of satisfaction and enjoyment in Physical Education from the autonomy and motivational climate. Universitas Psychologica, 15(2), 15–25.

Baena-Extremera, A., Gómez-López, M., Granero-Gallegos, A., & Ortiz-Camacho, M. del M. (2015). Predicting Satisfaction in Physical Education From Motivational Climate and Self-determined Motivation. Journal of Teaching in Physical Education, 34(2), 210–224. https://doi.org/10.1123/jtpe.2013-0165

Balaguer, I., Castillo, I., Duda, J. L., & García-Merita, M. (2011). Asociaciones entre la percepción del clima motivacional creado por el entrenador,orientaciones disposicionales de meta, regulaciones motivacionales y vitalidad subjetiva en jóvenes jugadoras de tenis = Associations between the perception of motivational climate created by coaches, dispositional goal orientations, forms of self-regulation and subjective vitality in young tennis players. Revista de Psicología Del Deporte, 20(1), 133–148.

Balaguer, I., Duda, J. L., & Castillo, I. (2017). Motivational Antecedents of Well-Being and Health Related Behaviors in Adolescents. Journal of Human Kinetics, 59(1), 121–130. https://doi.org/10.1515/hukin-2017-0152

Balaguer, I., Duda, J. L., & Crespo, M. (1999). Motivational climate and goal orientations as predictors of perceptions of improvement, satisfaction and coach ratings among tennis players. Scandinavian Journal of Medicine & Science in Sports, 9(6), 381.

Balaguer, I., Duda, J. L., Atienza, F. L., & Mayo, C. (2002). Situational and dispositional goals as predictors of perceptions of individual and team improvement, satisfaction and coach ratings among elite female handball teams. Psychology of Sport and Exercise, 3(4), 293–308. https://doi.org.lib.ttu.edu/10.1016/S1469-0292(01)00025-5

Baños, R., & Arrayales, E. (2020). Predicción del aburrimiento en la educación física a partir del clima motivacional (Prediction of boredom in physical education from the motivational climate). Retos, 38, 83–88. https://doi.org/10.47197/retos.v38i38.74301

BAO Xiao-ling, YANG Jun-min, & ZHANG Jiang-ying. (2010). The Intervention Research on the Influence of Subjective Well-being from Different Motivational Climate in P. E among College and Middle School Students. Journal of Beijing Sport University, 33(5), 96–100.

Barić, R. (2000). Verification of a motivational climate inventory in a sports setting. Kinesiology, 32(2), 106–116.

Barić, R. (2011). Psychological pressure and athletes' perception of motivational climate in team sports. Review of Psychology, 18(1), 45–49.

Barkoukis, V., Koidou, E., & Tsorbatzoudis, H. (2010). Effects of a motivational climate intervention on state anxiety, self-efficacy, and skill development in physical education. European Journal of Sport Science, 10(3), 167–177.
Barkoukis, V., Ntoumanis, N., & Thøgersen-Ntoumani, C. (2010). Developmental changes in achievement motivation and affect in physical education: Growth trajectories and demographic differences. Psychology of Sport & Exercise, 11(2), 83–90.
Bekiari, A. & Syrmpas, I. (2015). Coaches' verbal aggressiveness and motivational climate as predictors of athletes’ satisfaction. British Journal of Education, Society & Behavioural Science 9(4): 318-329, 2015.
Blecharz et al. 2014 Study 2 data
Blecharz, J., Horodyska, K., Zarychta, K., Adamiec, A., & Luszczynska, A. (2015). Intrinsic Motivation Predicting Performance Satisfaction in Athletes: Further Psychometric Evaluations of the Sport Motivation Scale-6. Polish Psychological Bulletin, 46(2), 309–319. https://doi.org/10.1515/ppb-2015-0037
Blecharz, J., Luszczynska, A., Tenenbaum, G., Scholz, U., & Cieslak, R. (2014). Self-Efficacy Moderates but Collective Efficacy Mediates between Motivational Climate and Athletes’ Well-Being. Applied Psychology: Health and Well-Being, 6(3), 280–299. Portico. https://doi.org/10.1111/aphw.12028
Boiché, J., Gourlan, M., & Rubin, L. (2018). Impact of a residential program on the psychological needs, motivation and physical activity of obese adults: A controlled trial based on Self-Determination Theory. Movement & Sport Sciences - Science & Motricité, 101, 33–40. https://doi.org/10.1051/sm/2018013
Boixadós i Anglès, M., & Cruz i Feliu, J. (1999). Relaciones entre clima motivacional y satisfacción, percepción de habilidad y actitudes de fairplay en futbolistas jóvenes = Relationships between motivational climate and satisfaction, perceived ability and fairplay attitudes in young soccer players. Revista de Psicología Social Aplicada, 9(1), 45–64.
Boixadós, M., Cruz, J., Torregrosa, M., & Valiente, L. (2003). Relationships Among Motivational Climate, Satisfaction, Perceived Ability, and Fair Play Attitudes in Young Soccer Players. Journal of Applied Sport Psychology, 16(4), 301–317. https://doi.org.lib.ttu.edu/10.1080/10413200490517977
Bono, B., & Livi, S. (2016). Motivazione al successo in atleti di élite: applicazione del 2X2 Achievement Goal Framework nel nuoto = Achievement motivation in elite athletes: Application of 2X2 Achievement Goal Framework in swimming. Rassegna Di Psicologia, 33(1), 51–66.

Borghouts, L., Slingerland, M., Weeldenburg, G., van Dijk-van Eijk, B., Laurijssens, S., Remmers, T., & Haerens, L. (2023). Effectiveness of a lesson study intervention on teacher behaviour and student motivation in physical education lessons. Physical Education and Sport Pedagogy, 28(2), 121–138. https://doi.org/10.1080/17408989.2021.1958175

Bortoli, L., Bertollo, M., & Robazza, C. (2009). Dispositional goal orientations, motivational climate, and psychobiosocial states in youth sport. Personality and Individual Differences, 47(1), 18–24. https://doi-org.lib-e2.lib.ttu.edu/10.1016/j.paid.2009.01.042

Bortoli, L., Bertollo, M., Comani, S., & Robazza, C. (2011). Competence, achievement goals, motivational climate, and pleasant psychobiosocial states in youth sport. Journal of Sports Sciences, 29(2), 171–180. https://doi.org/10.1080/02640414.2010.530675

Bortoli, L., Vitali, F., Di Battista, R., Ruiz, M. C., & Robazza, C. (2018). Initial validation of the psychobiosocial states in physical education (PBS-SPE) scale. Frontiers in Psychology, 9.

Braithwaite, R., Spray, C. M., & Warburton, V. E. (2011). Motivational climate interventions in physical education: A meta-analysis. Psychology of Sport and Exercise, 12(6), 628–638. https://doi-org.lib-e2.lib.ttu.edu/10.1016/j.psychsport.2011.06.005

Breiger, J., Cumming, S. P., Smith, R. E., Smoll, F., & Brewer, B. (2015). Winning, Motivational Climate, and Young Athletes' Competitive Experiences: Some Notable Sex Differences. International Journal of Sports Science & Coaching, 10(2/3), 395–411.

Breske, M. P., Fry, M. D., Fry, A. C., & Hogue, C. M. (2017). The effects of goal priming on cortisol responses in an ego-involving climate. Psychology of Sport and Exercise, 32, 74–82. https://doi.org/10.1016/j.psychsport.2017.06.001

Brinkman-Majewski, R. E., & Weiss, W. M. (2015). Examination of the Motivational Climate in the Athletic Training Room. Journal of Sport Behavior, 38(2), 143–160.

Brinkman-Majewski, R. E., & Weiss, W. M. (2018). The Motivational Climate and Intrinsic Motivation in the Rehabilitation Setting. Journal of Sport Rehabilitation, 27(5), 460–468. https://doi.org/10.1123/jsr.2016-0228

Brinkman-Majewski, R. E., & Weiss, W. M. (2022). The Influence of the Motivational Climate on Rehabilitation Behaviors and Patient Satisfaction. Journal of Sport Rehabilitation, 31(8), 1016–1022. https://doi.org/10.1123/jsr.2021-0369

Brisimis, E., Krommidas, C., Syrmpas, I., Karamitrou, A., Hatzigeorgiadis, A., & Comoutos, N. (2022). Motivational Climate, Basic Psychological Needs, and Students' Self-Talk in Physical Education. The Physical Educator, 79(3), 280–304. https://doi.org/10.18666/tpe-2022-v79-i3-10851

Broo, N., Ballart, P., Juan, B., Valls, A., & Latinjak, A. (2012). Motivación Situacional Y Estado Afectivo en Clases Dirigidas De Actividad Física. Motricidad: European Journal of Human Movement, 29, 147–158.

Brown, T. C., & Fry, M. D. (2014a). Motivational Climate, Staff and Members' Behaviors, and Members' Psychological Well-Being at a National Fitness Franchise. Research Quarterly for Exercise and Sport, 85(2), 208–217. https://doi.org/10.1080/02701367.2014.893385

Brown, T. C., & Fry, M. D. (2014b). College Exercise Class Climates, Physical Self-Concept, and Psychological Well-Being. Journal of Clinical Sport Psychology, 8(3), 299–313. https://doi.org/10.1123/jcsp.2014-0031

Brown, T. C., Fry, M. D., & Moore, E. W. G. (2017). A motivational climate intervention and exercise-related outcomes: A longitudinal perspective. Motivation Science, 3(4), 337–353. https://doi.org/10.1037/mot0000059

Brown, T. C., Fry, M. D., & Moore, E. W. G. (2017). A motivational climate intervention and exercise-related outcomes: A longitudinal perspective. Motivation Science, 3(4), 337–353. https://doi.org/10.1037/mot0000059

Brown, T. C., Fry, M. D., Wilkinson, T. J., Breske, M. P., & Susumu Iwasaki. (2019). Motivational Climate and Athletes' Likelihood of Reporting Concussions in a Youth Competitive Soccer League. Journal of Sport Behavior, 42(1), 29–47.

Bryan, C. L., & Solmon, M. A. (2012). Student Motivation in Physical Education and Engagement in Physical Activity. Journal of Sport Behavior, 35(3), 267–285.

Calvo, C., & Topa, G. (2019). Leadership and Motivational Climate: The Relationship with Objectives, Commitment, and Satisfaction in Base Soccer Players. Behavioral Sciences, 9(3), 29. https://doi.org/10.3390/bs9030029

Carr, S., & Wyon, M. (2003). The Impact of Motivational Climate on Dance Students' Achievement Goals, Trait Anxiety, and Perfectionism. Journal of Dance Medicine & Science, 7(4), 105–114. https://doi.org/10.1177/1089313x0300700401

Carr, S., Phil, M., & Wyon, M. (2003). The impact of motivational climate on dance students' achievement goals, trait anxiety, and perfectionism. Journal of Dance Medicine & Science, 7(4), 105–114.

Castillo Jiménez, N., López-Walle, J. M., Tomás, I., & Balaguer, I. (2017). Relación del clima empowering con la motivación autodeterminada a través de la satisfacción de las necesidades psicológicas básicas. Journal of Sport Psychology / Revista de Psicología Del Deporte, 26, 33–39.

Castillo, I., Duda, J. L., Álvarez, M. S., Mercé, J., & Balaguer, I. (2011). Clima motivacional, metas de logro de aproximación y evitación y bienestar en futbolistas cadetes = Motivational climate, approach-avoidance achievement goals and well-being in young soccer players. Revista de Psicología Del Deporte, 20(1), 149–164.

Castillo, I., Ramis, Y., Cruz, J., & Balaguer, I. (2015). Formación de Entrenadores de Fútbol Base en el Proyecto PAPA = Grassroots coaches' training in the PAPA project. Revista de Psicología Del Deporte, 24(1), 131–138.

Castro-Sánchez, M., Zurita-Ortega, F., Chacón-Cuberos, R., & Lozano-Sánchez, A. M. (2018). Clima motivacional y niveles de ansiedad en futbolistas de categorías inferiores (Motivational climate and levels of anxiety in soccer players of lower divisions). Retos, 35, 164–169. https://doi.org/10.47197/retos.v0i35.63308

Castro-Sánchez, M., Zurita-Ortega, F., Zafra-Santos, E., Rodríguez-Fernández, S., Chacón-Cuberos, R., & Valdivia-Moral, P. (2019). MOTIVACIÓN EN LA PRÁCTICA DEL JUDO EN DEPORTISTAS NO PROFESIONALES. Revista Internacional de Medicina y Ciencias de La Actividad Física y Del Deporte, 19(74). https://doi.org/10.15366/rimcafd2019.74.005

Cecchini, J. A., Carriedo, A., & Méndez-Giménez, A. (2019). Testing a circular, feedback model in physical education from self-determination theory. Journal of Educational Research, 112(4), 473–482. https://doi.org/10.1080/00220671.2018.1555788

Cecchini, J. A., Carriedo, A., & Méndez-Giménez, A. (2019). Testing a circular, feedback model in physical education from self-determination theory. The Journal of Educational Research, 112(4), 473–482. https://doi.org/10.1080/00220671.2018.1555788

Cecchini, J. A., Fernandez-Rio, J., Mendez-Gimenez, A., Cecchini, C., & Martins, L. (2014). Epstein's TARGET Framework and Motivational Climate in Sport: Effects of a Field-Based, Long-Term Intervention Program. International Journal of Sports Science & Coaching, 9(6), 1325–1340. https://doi.org/10.1260/1747-9541.9.6.1325

Cecchini, J. A., González, C., Carmona, Á. M., & Contreras, O. (2004). Relaciones entre clima motivacional, la orientación de meta, la motivación intrínseca, la auto-confianza, la ansiedad y el estado de ánimo en jóvenes deportistas = Relationships among motivational climate, achievement goals, intrinsic motivation, self-confidence, anxiety, and mood in young sport players. Psicothema, 16(1), 104–109.

Cecchini, J. A., Gonzalez, C., Carmona, A. M., Arruza, J., Escarti, A., & Balague, G. (2001). The Influence of the Physical Education Teacher on Intrinsic Motivation, Self-Confidence, Anxiety, and Pre- and Post-Competition Mood States. European Journal of Sport Science, 1(4), 1.

Cecchini, J. A., González, C., Prado, J. L., & Brustad, R. J. (2005). Relación del Clima Motivacional Percibido con la Orientación de Meta, la Motivación Intrínseca y las Opiniones y Conductas de Fair Play = The relationship between perceived motivational climate and goal orientation, intrinsic motivation and fair play conducts and viewpoints. Revista Mexicana de Psicología, 22(2), 469–479.

Cervelló, E. M., & Santos-Rosa, F. J. (2001). Motivation in sport: An achievement goal perspective in young Spanish recreational athletes. Perceptual and Motor Skills, 92(2), 527–534. https://doi.org.lib.ttu.edu/10.2466/PMS.92.2.527-534

Chacón-Cuberos, R., Castro-Sánchez, M., Pérez-Turpin, J. A., Olmedo-Moreno, E. M., & Zurita Ortega, F. (2019). Levels of Physical Activity Are Associated With the Motivational Climate and Resilience in University Students of Physical Education From Andalucía: An Explanatory Model. Frontiers in Psychology, 10. https://doi.org/10.3389/fpsyg.2019.01821

Chan, D. K., Lonsdale, C., & Fung, H. H. (2011). Influences of coaches, parents, and peers on the motivational patterns of child and adolescent athletes. Scandinavian Journal of Medicine & Science in Sports, 22(4), 558–568. Portico. https://doi.org/10.1111/j.1600-0838.2010.01277.x

Chen, L. H., Wu, C.-H., Ni, Y.-L., & Kuo, C.-C. (2021). The differential interaction effect of mastery and performance climate on athletes' emotional and physical exhaustion: The role of athletes' gratitude. Sport, Exercise, and Performance Psychology, 10(3), 394–407. https://doi.org/10.1037/spy0000257

Cheon, S. H., Reeve, J., & Ntoumanis, N. (2019). An intervention to help teachers establish a prosocial peer climate in physical education. Learning and Instruction, 64, 101223. https://doi.org/10.1016/j.learninstruc.2019.101223

Chu, T. L. (Alan), Treacy, A., Moore, E. W. G., Petrie, T. A., Albert, E., & Zhang, T. (2023). Intersectionality matters: Gender, race/ethnicity, and sport level differentiate perceived coach-created motivational climates and psychological needs. Sport, Exercise, and Performance Psychology. https://doi-org.lib-e2.lib.ttu.edu/10.1037/spy0000331.supp (Supplemental)

Chu, T. L. (Alan), Treacy, A., Moore, E. W. G., Petrie, T. A., Albert, E., & Zhang, T. (2023). Intersectionality matters: Gender, race/ethnicity, and sport level differentiate perceived coach-created motivational climates and psychological needs. Sport, Exercise, and Performance Psychology. https://doi/org.10.1037/spy0000331.supp

Chu, T. L. (Alan), Zhang, X., Lee, J., & Zhang, T. (2021). Perceived coach-created environment directly predicts high school athletes' physical activity during sport. International Journal of Sports Science & Coaching, 16(1), 70–80. https://doi.org/10.1177/1747954120959733

Cid, L., Pires, A., Borrego, C., Duarte-Mendes, P., Teixeira, D. S., Moutão, J. M., & Monteiro, D. (2019). Motivational determinants of physical education grades and the intention to practice sport in the future. PLOS ONE, 14(5), e0217218. https://doi.org/10.1371/journal.pone.0217218

Cid, L., Pires, A., Borrego, C., Duarte-Mendes, P., Teixeira, D. S., Moutão, J. M., & Monteiro, D. (2019). Motivational determinants of physical education grades and the intention to practice sport in the future. PLoS ONE, 14(5). https://doi-org.lib-e2.lib.ttu.edu/10.1371/journal.pone.0217218

Clancy, R. B., Herring, M. P., MacIntyre, T. E., & Campbell, M. J. (2016). A review of competitive sport motivation research. Psychology of Sport & Exercise, 27, 232–242.

Connaughton, D., Wadey, R., Hanton, S., & Jones, G. (2008). The development and maintenance of mental toughness: Perceptions of elite performers. Journal of Sports Sciences, 26(1), 83–95.

Connaughton, D., Wadey, R., Hanton, S., & Jones, G. (2008). The development and maintenance of mental toughness: Perceptions of elite performers. Journal of Sports Sciences, 26(1), 83–95. https://doi.org/10.1080/02640410701310958

Conroy, D. E., & Douglas Coatsworth, J. (2007). Assessing autonomy-supportive coaching strategies in youth sport. Psychology of Sport and Exercise, 8(5), 671–684. https://doi.org/10.1016/j.psychsport.2006.12.001

Coudevylle, G. R., & Martin Ginis, K. A. (2007). An experimental investigation of determinants and consequences of self-handicapping strategies across motivational climates. Journal of Sport & Exercise Psychology, 29, S153–S154.

Cox, A., & Williams, L. (2008). The Roles of Perceived Teacher Support, Motivational Climate, and Psychological Need Satisfaction in Students' Physical Education Motivation. Journal of Sport and Exercise Psychology, 30(2), 222–239. https://doi.org/10.1123/jsep.30.2.222

Cronin, L., Marchant, D., Allen, J., Mulvenna, C., Cullen, D., Williams, G., & Ellison, P. (2019). Students' perceptions of autonomy-supportive versus controlling teaching and basic need satisfaction versus frustration in relation to life skills development in PE. Psychology of Sport and Exercise, 44, 79–89. https://doi.org.l/10.1016/j.psychsport.2019.05.003

Cumming, S. P., Smith, R. E., Smoll, F. L., Standage, M., & Grossbard, J. R. (2008). Development and validation of the Achievement Goal Scale for Youth Sports. Psychology of Sport & Exercise, 9(5), 686–703.

Cumming, S. P., Smoll, F. L., Smith, R. E., & Grossbard, J. R. (2007). Is Winning Everything? The Relative Contributions of Motivational Climate and Won-Lost Percentage in Youth Sports. Journal of Applied Sport Psychology, 19(3), 322–336. https://doi.org/10.1080/10413200701342640

Cunningham, G. B., & Xiang, P. (2008). Testing the mediating role of perceived motivational climate in the relationship between achievement goals and satisfaction: Are these relationships invariant across sex? Journal of Teaching in Physical Education, 27(2), 192–204. https://doi-org.lib-e2.lib.ttu.edu/10.1123/jtpe.27.2.192

Curran, T., Hill, A. P., Hall, H. K., & Jowett, G. E. (2015). Relationships Between the Coach-Created Motivational Climate and Athlete Engagement in Youth Sport. Journal of Sport and Exercise Psychology, 37(2), 193–198. https://doi.org/10.1123/jsep.2014-0203

Decroos, S., Lines, R. L. J., Morgan, P. B. C., Fletcher, D., Sarkar, M., Fransen, K., Boen, F., & Vande Broek, G. (2017). Development and validation of the Characteristics of Resilience in Sports Teams Inventory. Sport, Exercise, and Performance Psychology, 6(2), 158–178. https://doi.org/10.1037/spy0000089

Di Battista, R., Robazza, C., Ruiz, M. C., Bertollo, M., Vitali, F., & Bortoli, L. (2019). Student intention to engage in leisure-time physical activity: The interplay of task-involving climate, competence need satisfaction and psychobiosocial states in physical education. European Physical Education Review, 25(3), 761–777. https://doi.org/10.1177/1356336x18770665

Digelidis, N., Kotsaki, Z., & Papaioannou, A. (2005). Differences between Junior and Senior High School Students Concerning Intrinsic - Extrinsic Motivation in the Contextual Level, Goal Orientations, Motivational Climate and Perceived Athletic Ability in Greek Athletic Classes. Inquiries in Sport & Physical Education, 3(1), 77–89.

Digellidis, N., & Krommidas, H. (2008). Fair Play in Physical Education Classes: Differences between Sex, Class and the Relationship between Fair Play and Lesson Satisfaction, Perceived Motivational Climate and Goal Orientations. Inquiries in Sport & Physical Education, 6(2), 1–14.

Dorsch, T. E., Smith, A. L., & Dotterer, A. M. (2016). Individual, relationship, and context factors associated with parent support and pressure in organized youth sport. Psychology of Sport and Exercise, 23, 132–141. https://doi.org/10.1016/j.psychsport.2015.12.003

Draugelis, S., Martin, J., & Garn, A. (2014). Psychosocial Predictors of Well-Being in Collegiate Dancers. The Sport Psychologist, 28(1), 1–9. https://doi.org/10.1123/tsp.2012-0093

Duda, J. L., Quested, E., Haug, E., Samdal, O., Wold, B., Balaguer, I., Castillo, I., Sarrazin, P., Papaioannou, A., Ronglan, L. T., Hall, H., & Cruz, J. (2013). Promoting Adolescent health through an intervention aimed at improving the quality of their participation in Physical Activity (PAPA): Background to the project and main trial protocol. International Journal of Sport and Exercise Psychology, 11(4), 319–327. https://doi.org/10.1080/1612197x.2013.839413

Duguay, A. M., Loughead, T. M., & Munroe-Chandler, K. J. (2016). The Development, Implementation, and Evaluation of an Athlete Leadership Development Program With Female Varsity Athletes. The Sport Psychologist, 30(2), 154–166. https://doi.org/10.1123/tsp.2015-0050

Elsborg, P., Appleton, P. R., Pons, J., Wikman, J. M., Bentsen, P., & Nielsen, G. (2023). Factorial validity, predictive validity and measurement invariance of the Danish version of the coach-created Empowering Disempowering Motivational Climate Questionnaire (EDMCQ-C). Journal of Sports Sciences, 41(8), 715–726. https://doi.org/10.1080/02640414.2023.2230707

Elsborg, P., Appleton, P., Wikman, J. M., & Nielsen, G. (2023). The associations between motivational climate, basic psychological needs and dropout in volleyball – A comparison across competitive levels. European Journal of Sport Science, 23(3), 393–403. https://doi.org/10.1080/17461391.2022.2041100

Erikstad, M. K., Høigaard, R., Côté, J., Turnnidge, J., & Haugen, T. (2021). An Examination of the Relationship Between Coaches' Transformational Leadership and Athletes' Personal and Group Characteristics in Elite Youth Soccer. Frontiers in Psychology, 12. https://doi.org/10.3389/fpsyg.2021.707669

Escartí, A., & Gutiérrez, M. (2001). Influence of the motivational climate in physical education on the intention to practice physical activity or sport. European Journal of Sport Science, 1(4), 1–12. https://doi.org/10.1080/17461390100071406

Eys, M. A., Jewitt, E., Evans, M. B., Wolf, S., Bruner, M. W., & Loughead, T. M. (2013). Coach-Initiated Motivational Climate and Cohesion in Youth Sport. Research Quarterly for Exercise and Sport, 84(3), 373–383. https://doi.org/10.1080/02701367.2013.814909

Fenton, S. A. M., Duda, J. L., Appleton, P. R., & Barrett, T. G. (2017). Empowering youth sport environments: Implications for daily moderate-to-vigorous physical activity and adiposity. Journal of Sport & Health Science, 6(4), 423–433.

Fernandes, V., Silva, S., & Dias, C. (2022). Clima motivacional e treino de competências psicológicas: Um estudo na ginástica de trampolins. Revista Portuguesa de Ciências Do Desporto, 22(2), 28–39. https://doi.org/10.5628/rpcd.22.02.28

Fernandez-Rio, J., & Morales-Sallés, P. (2020). Student-designed games in secondary education Effects and perspectives from students and teachers. The Journal of Educational Research, 113(3), 204–212. https://doi-org.lib-e2.lib.ttu.edu/10.1080/00220671.2020.1778614

Fernández-Rio, J., Cecchini, J. A., & Méndez-Giménez, A. (2017). Does Context, Practice or Competition Affect Female Athletes' Achievement Goal Dominance, Goal Pursuit, Burnout and Motivation? Journal of Human Kinetics, 59(1), 91–105. https://doi.org/10.1515/hukin-2017-0150

Fernandez-Rio, J., Méndez-Giménez, A., & Estrada, J. A. C. (2014). A cluster analysis on students' perceived motivational climate: Implications on psycho-social variables. The Spanish Journal of Psychology, 17.

Fernandez-Rio, J., Sanz, N., Fernandez-Cando, J., & Santos, L. (2017). Impact of a sustained cooperative learning intervention on student motivation. Physical Education and Sport Pedagogy, 22(1), 89–105.

Fry, M. D., Hogue, C. M., Iwasaki, S., & Solomon, G. B. (2021). The Relationship Between the Perceived Motivational Climate in Elite Collegiate Sport and Athlete Psychological Coping Skills. Journal of Clinical Sport Psychology, 15(4), 334–350. https://doi.org/10.1123/jcsp.2020-0002

Fuster-Parra, P., García-Mas, A., Ponseti, F. J., Palou, P., & Cruz, J. (2013). A Bayesian network to discover relationships between negative features in sport: a case study of teen players. Quality & Quantity, 48(3), 1473–1491. https://doi.org/10.1007/s11135-013-9848-y

García-Calvo, T., Leo, F. M., Gonzalez-Ponce, I., Sánchez-Miguel, P. A., Mouratidis, A., & Ntoumanis, N. (2014). Perceived coach-created and peer-created motivational climates and their associations with team cohesion and athlete satisfaction: evidence from a longitudinal study. Journal of Sports Sciences, 32(18), 1738–1750. https://doi.org/10.1080/02640414.2014.918641

Garcia-Mas, A., Fuster-Parra, P., Ponseti, F. J., Palou, P., Olmedilla, A., & Cruz, J. (2015). Análisis bayesiano de la motivación, el clima motivacional y la ansiedad en jóvenes jugadores de equipo = A Bayesian analysis of the motivation, motivational climate and anxiety in young competitive team players. Anales de Psicología, 31(1), 355–366. https://doi-org.lib-e2.lib.ttu.edu/10.6018/analesps.31.1.167531

Garcia-Mas, A., Palou, P., Smith, R. E. ., Ponseti, X., Almeida, P., Lameiras, J., Jiménez, R., & Leiva, A. (2011). Ansiedad competitiva y clima motivacional en jóvenes futbolistas de competición, enrelación con las habilidades y el rendimiento percibido por susentrenadores = Performance anxiety and motivational climate in young competitive soccer players in relation to performance and skills perceived by their coaches. Revista de Psicología Del Deporte, 20(1), 197–207.

Gayman, A. M., Eys, M., & Coleman, T. (2022). Group dynamics in Canadian recreational team sports later in life. Psychology of Sport & Exercise, 60, N.PAG.

Giannoudis, G., Digelidis, N., & Papaioannou, A. (2009). The Motivation Climate and Perceived Sport Teachers' Behavior toward Disciplined - Undisciplined Students. Inquiries in Sport & Physical Education, 7(1), 10–21.

Gil-Arias, A., Claver, F., Práxedes, A., Villar, F. D., & Harvey, S. (2020). Autonomy support, motivational climate, enjoyment and perceived competence in physical education: Impact of a hybrid teaching games for understanding/sport education unit. European Physical Education Review, 26(1), 36–53.

Gillham, A., Burton, D., & Gillham, E. (2013). Going beyond Won-Loss Record to Identify Successful Coaches: Development and Preliminary Validation of the Coaching Success Questionnaire-2. International Journal of Sports Science & Coaching, 8(1), 115–138. https://doi.org/10.1260/1747-9541.8.1.115

Girard, S., Desbiens, J.-F., & Hogue, A.-M. (2023). Effects of a training course on creation of an empowering motivational climate in physical education: a quasi-experimental study. Physical Education & Sport Pedagogy, 28(1), 56–75.

Girard, S., Lemoyne, J., Blais, D., & St-Amand, J. (2022). An analysis of mechanisms underlying social goals in physical education: a comparison between ordinary and special classes. Physical Education and Sport Pedagogy, 27(3), 320–337. https://doi.org/10.1080/17408989.2021.1879767

Gjesdal, S., Haug, E. M., & Ommundsen, Y. (2019). A conditional process analysis of the coach-created mastery climate, task goal orientation, and competence satisfaction in youth soccer: The moderating role of controlling coach behavior. Journal of Applied Sport Psychology, 31(2), 203–217. https://doi.org/10.1080/10413200.2017.1413690

Gjesdal, S., Stenling, A., Solstad, B. E., & Ommundsen, Y. (2018). A study of coach-team perceptual distance concerning the coach-created motivational climate in youth sport. Scandinavian Journal of Medicine & Science in Sports, 29(1), 132–143. Portico. https://doi.org/10.1111/sms.13306

Goal Orientation and Perceptions of the Motivational Climate Initiated by Parents. (1996). Pediatric Exercise Science, 8(2), 122–129.

Gómez-López, M., Chicau Borrego, C., Marques da Silva, C., Granero-Gallegos, A., & González-Hernández, J. (2020). Effects of Motivational Climate on Fear of Failure and Anxiety in Teen Handball Players. International Journal of Environmental Research and Public Health, 17(2), 592. https://doi.org/10.3390/ijerph17020592

Gómez-López, M., Granero-Gallegos, A., Baena-Extremera, A., Amador, C. B., & Pérez Quero, F. J. (2015). Efectos de Interacción de Sexo y Práctica de Ejercicio Físico sobre las Estrategias para la Disciplina, Motivación y Satisfacción con la Educación Física = Interaction effects of sex and practice of physical exercise on strategies for discipline, motivation and satisfaction with physical education. Revista Iberoamericana de Diagnóstico y Evaluación Psicologica, 40(2), 6–16.

GONZÁLEZ-CALVO, G., BORES-GARCÍA, D., HORTIGÜELA-ALCALÁ, D., & BARBA-MARTÍN, R. A. (2018). Adherence to a Physical Exercise Program in School and Extracurricular Activities. / Adherència a un programa d'exercici físic en els àmbits educatius i extraescolar. Apunts: Educació Física i Esports, 134, 39–54.
González-Calvo, G., Bores-García, D., Hortigüela-Alcalá, D., & Barba-Martín, R. A. (2018). Adherence to a Physical Exercise Program in School and Extracurricular Activities. / Adherència a un programa d'exercici físic en els àmbits educatius i extraescolar. Apunts: Educació Física i Esports, 134, 39–54.
González-Calvo, G., Bores-García, D., Hortigüela-Alcalá, D., & Barba-Martín, R. A. (2018). Adherència a un programa d'exercici físic en els àmbits educatius i extraescolar. Apunts Educació Física i Esports, 134, 39–54. https://doi.org/10.5672/apunts.2014-0983.cat.(2018/4).134.03
González-Cutre, D., & Sicilia, Á. (2012). Motivation and Exercise Dependence: A Study Based on Self-Determination Theory. Research Quarterly for Exercise and Sport, 83(2), 318–329. https://doi.org/10.5641/027013612800745194
Gotzaridis, C., Papaioannou, A., Antoniou, P., & Albanidis, E. (2007). The Effect of an Interdisciplinary Teaching Approach on Seventh-Grade Pupils' Motivation in Physical Education Class. Inquiries in Sport & Physical Education, 5(1), 52–62.
Granero-Gallegos, Antonio, Manuel Gómez-López, Nuria Rodríguez-Suárez, J. Arturo Abraldes, Marianna Alesi, and Antonino Bianco. 2017. "Importance of the Motivational Climate in Goal, Enjoyment, and the Causes of Success in Handball Players." Frontiers in Psychology 8 (December). doi:10.3389/fpsyg.2017.02081.
Gråstén, A., & Watt, A. (2017). A Motivational Model of Physical Education and Links to Enjoyment, Knowledge, Performance, Total Physical Activity and Body Mass Index. Journal of Sports Science & Medicine, 16(3), 318–327.
Gråstén, A., Jaakkola, T., Liukkonen, J., Watt, A., & Yli-Piipari, S. (2012). Prediction of enjoyment in school physical education. Journal of Sports Science & Medicine, 11(2), 260–269.
Gray, S., Sproule, J., & Morgan, K. (2009). Teaching team invasion games and motivational climate. / Der Unterricht in Mannschaftssportspielarten und das motivationale Klima. European Physical Education Review, 15(1), 65–89.
Griffin, K., Meaney, K., & Hart, M. (2013). The Impact of a Mastery Motivational Climate on Obese and Overweight Children's Commitment to and Enjoyment of Physical Activity: A Pilot Study. American Journal of Health Education, 44(1), 1–8.
Grove, J. R., & Weigland, D. A. (1999). Goal orientations and climate. Journal of Sport & Exercise Psychology, 21(3), 295.

Gu, X., & Solmon, M. A. (2016). Motivational processes in children’s physical activity and health-related quality of life. Physical Education and Sport Pedagogy, 21(4), 407–424. https://doi.org/10.1080/17408989.2015.1017456

Gu, X., & Solmon, M. A. (2016). Motivational processes in children’s physical activity and health-related quality of life. Physical Education and Sport Pedagogy, 21(4), 407–424. https://doi.org/10.1080/17408989.2015.1017456

Gustafsson, H., Hill, A. P., Stenling, A., & Wagnsson, S. (2015). Profiles of perfectionism, parental climate, and burnout among competitive junior athletes. Scandinavian Journal of Medicine & Science in Sports, 26(10), 1256–1264. Portico. https://doi.org/10.1111/sms.12553

Gutiérrez-García, P., Alejandra Herrera-Nevárez, M., Moueth-Cabrera, M. T., Andrés González-Fimbres, R., Ramírez-Siqueiros, M. G., & Castillo-Jiménez, N. (2023). Empoderamiento y desempoderamiento, satisfacción, frustración, motivación e intención futura de práctica en paralímpicos. Diferencias demográficas. / Empowering and disempowering, sastisfaction, frustration, motivation and future intention to practice in Paralympians. Demographic differences. Retos: Nuevas Perspectivas de Educación Física, Deporte y Recreación, 49, 532–541.

Gutiérrez-García, P., López-Walle, J. M., Tomás, I., Tristán, J., & Balaguer, I. (2019). Relación entre clima empowering y diversión en pitchers de béisbol: el papel moderador de la motivación autónoma. Cuadernos de Psicología Del Deporte, 19(1), 166–177. https://doi.org/10.6018/cpd.353081

Gutiérrez-García, P., López-Walle, J., Tomás, I., Tristán, J., & Balaguer, I. (2019). Relación entre clima empowering y diversión en pitchers de béisbol: el papel moderador de la motivación autónoma. / Relationship between empowering climate and enjoyment in baseball pitchers: the moderator role of autonomous motivation. Cuadernos de Psicología Del Deporte, 19(1), 166–177.

Guzmán, J. F., & García, C. G. (2014). Psychological Well-Being in Dancers: A Social Cognitive Analysis. / Bienestar Psicológico en Bailarines: Un Análisis Social Cognitivo. International Journal of Medicine & Science of Physical Activity & Sport / Revista Internacional de Medicina y Ciencias de La Actividad Física y Del Deporte, 14(56), 687–704.

Habeeb, C. M., Barbee, J., & Raedeke, T. D. (2023). Association of parent, coach, and peer motivational climate with high school athlete burnout and engagement: Comparing mediation and moderation models. Psychology of Sport and Exercise, 68, 102471. https://doi.org/10.1016/j.psychsport.2023.102471

Halliburton, A. L., & Weiss, M. R. (2002). Sources of Competence Information and Perceived Motivational Climate among Adolescent Female Gymnasts Varying in Skill Level. Journal of Sport and Exercise Psychology, 24(4), 396–419. https://doi.org/10.1123/jsep.24.4.396

Hancox, J. E., Quested, E., Ntoumanis, N., & Duda, J. L. (2017). Teacher-created social environment, basic psychological needs, and dancers' affective states during class: A diary study. Personality and Individual Differences, 115, 137–143. https://doi-org.lib-e2.lib.ttu.edu/10.1016/j.paid.2016.03.033

Hancox, J. E., Quested, E., Ntoumanis, N., & Duda, J. L. (2017). Teacher-created social environment, basic psychological needs, and dancers' affective states during class: A diary study. Personality and Individual Differences, 115, 137–143. https://doi-org.lib-e2.lib.ttu.edu/10.1016/j.paid.2016.03.033

Haraldsen, H. M., Nordin-Bates, S. M., Abrahamsen, F. E., & Halvari, H. (2020). Thriving, Striving, or Just Surviving? TD Learning Conditions, Motivational Processes and Well-Being Among Norwegian Elite Performers in Music, Ballet, and Sport. Roeper Review, 42(2), 109–125. https://doi.org/10.1080/02783193.2020.1728796

Harris, B. S., Blom, L. C., & Visek, A. J. (2013). Assessment in youth sport: Practical issues and best practice guidelines. The Sport Psychologist, 27(2), 201–211.

Harris, B. S., Blom, L. C., & Visek, A. J. (2013). Assessment in youth sport: Practical issues and best practice guidelines. The Sport Psychologist, 27(2), 201–211. https://doi-org.lib-e2.lib.ttu.edu/10.1123/tsp.27.2.201

Harwood, C. G., Caglar, E., Thrower, S. N., & Smith, J. M. J. (2019). Development and Validation of the Parent-Initiated Motivational Climate in Individual Sport Competition Questionnaire. Frontiers in Psychology, 10. https://doi.org/10.3389/fpsyg.2019.00128

Harwood, C. G., Keegan, R. J., Smith, J. M. J., & Raine, A. S. (2015). A systematic review of the intrapersonal correlates of motivational climate perceptions in sport and physical activity. Psychology of Sport & Exercise, 18, 9–25.

Haugen, T., Riesen, J. F., Østrem, K., Høigaard, R., & Erikstad, M. K. (2020). The Relationship between Motivational Climate and Personal Treatment Satisfaction among Young Soccer Players in Norway: The Moderating Role of Supportive Coach-Behaviour. Sports, 8(12), 162. https://doi.org/10.3390/sports8120162

Hill, D. M., & Shaw, G. (2013). A qualitative examination of choking under pressure in team sport. Psychology of Sport & Exercise, 14(1), 103–110.

Hodge, K., & Gucciardi, D. F. (2015). Antisocial and Prosocial Behavior in Sport: The Role of Motivational Climate, Basic Psychological Needs, and Moral Disengagement. Journal of Sport and Exercise Psychology, 37(3), 257–273. https://doi.org/10.1123/jsep.2014-0225

Hogue, C. M. (2020). Achievement goal theory-based psychological skills training session buffers youth athletes' psychophysiological responses to performance stress. Psychology of Sport and Exercise, 51, 101792. https://doi.org/10.1016/j.psychsport.2020.101792

Hogue, C. M. (2020). Achievement goal theory-based psychological skills training session buffers youth athletes' psychophysiological responses to performance stress. Psychology of Sport and Exercise, 51. https://doi-org.lib-e2.lib.ttu.edu/10.1016/j.psychsport.2020.101792

Hogue, C. M., Fry, M. D., & Fry, A. C. (2017). The differential impact of motivational climate on adolescents' psychological and physiological stress responses. Psychology of Sport and Exercise, 30, 118–127. https://doi.org/10.1016/j.psychsport.2017.02.004

Hogue, C. M., Fry, M. D., & Fry, A. C. (2021). The protective impact of learning to juggle in a caring, task-involving climate versus an ego-involving climate on participants' inflammation, cortisol, and psychological responses. International Journal of Sport and Exercise Psychology, 19(4), 650–667. https://doi.org/10.1080/1612197x.2019.1696868

Hogue, C. M., Fry, M. D., & Iwasaki, S. (2019). The impact of the perceived motivational climate in physical education classes on adolescent greater life stress, coping appraisals, and experience of shame. Sport, Exercise, and Performance Psychology, 8(3), 273–289. https://doi.org/10.1037/spy0000153

Hogue, C. M., Fry, M. D., Fry, A. C., & Pressman, S. D. (2013). The Influence of a Motivational Climate Intervention on Participants' Salivary Cortisol and Psychological Responses. Journal of Sport & Exercise Psychology, 35(1), 85–97.

Hogue, C. M., Fry, M. D., Fry, A. C., & Pressman, S. D. (2013). The Influence of a Motivational Climate Intervention on Participants' Salivary Cortisol and Psychological Responses. Journal of Sport & Exercise Psychology, 35(1), 85–97.

Holgado, F. P., Navas, L., & López-Núñez, M. (2010). Orientaciones de Meta en el deporte: un modelo causal. European Journal of Education and Psychology, 3(1), 19. https://doi.org/10.30552/ejep.v3i1.50

Horn, T. S. (2015). Social Psychological and Developmental Perspectives on Early Sport Specialization. Kinesiology Review, 4(3), 248–266.

Hortiguela, D., Gutierrez-Garcia, C., & Hernando-Garijo, A. (2017). Combat versus team sports: the effects of gender in a climate of peer-motivation, and levels of fun and violence in physical education students. / Sporty walki kontra sporty zespołowe: wpływ płci na atmosferę motywacji, zabawy i przemocy wśród uczniów-rówieśników w trakcie zajęć wychowania fizycznego. Ido Movement for Culture. Journal of Martial Arts Anthropology, 17(3), 11–20.

Howell, M., Hwang, Y., Deng, Y., Kim, T., Walker, B., & Yli-Piipari, S. (2021). Associations Of Coach-created Motivational Climate And The Prevalence Of Anxiety In Student-athletes. Medicine & Science in Sports & Exercise, 53(8S), 310–310. https://doi.org/10.1249/01.mss.0000762752.02687.02

Huddleston, H., Fry, M. D., & Brown, T. C. (2012). Corporate fitness members' perceptions of the environment and their intrinsic motivation. Revista de Psicología Del Deporte, 21(1), 15–23.

Into, S., Perttula, V.-M., Aunola, K., Sorkkila, M., & Ryba, T. V. (2020). Relationship between coaching climates and student-athletes' symptoms of burnout in school and sports. Sport, Exercise, and Performance Psychology, 9(3), 341–356. https://doi.org/10.1037/spy0000180

Isoard-Gautheur, S., Ginoux, C., & Trouilloud, D. (2022). Associations between peer motivational climate and athletes' sport-related well-being: Examining the mediating role of motivation using a multi-level approach. Journal of Sports Sciences, 40(5), 550–560. https://doi.org/10.1080/02640414.2021.2004680

Isoard-Gautheur, S., Guillet-Descas, E., & Duda, J. L. (2013). How to achieve in elite training centers without burning out? An achievement goal theory perspective. Psychology of Sport and Exercise, 14(1), 72–83. https://doi.org/10.1016/j.psychsport.2012.08.001

JAAKKOLA, T., BARKOUKIS, V., HUHTINIEMI, M., SALIN, K., SEPPÄLÄ, S., LAHTI, J., & WATT, A. (2019). Enjoyment and anxiety in Finnish physical education - achievement goals and self-determination perspectives. Journal of Physical Education & Sport, 19(3), 1619–1629.

Jaakkola, T., Ntoumanis, N., & Liukkonen, J. (2015). Motivational climate, goal orientation, perceived sport ability, and enjoyment within <scp>F</scp>innish junior ice hockey players. Scandinavian Journal of Medicine & Science in Sports, 26(1), 109–115. Portico. https://doi.org/10.1111/sms.12410

Jaakkola, T., Wang, C. K. J., Soini, M., & Liukkonen, J. (2015). Students' Perceptions of Motivational Climate and Enjoyment in Finnish Physical Education: A Latent Profile Analysis. Journal of Sports Science & Medicine, 14(3), 477–483.

Jaakkola, T., Yli-Piipari, S., Barkoukis, V., & Liukkonen, J. (2015). Relationships among perceived motivational climate, motivational regulations, enjoyment, and PA participation among Finnish physical education students. International Journal of Sport and Exercise Psychology, 15(3), 273–290. https://doi.org/10.1080/1612197x.2015.1100209

Jaakkola, T., Yli-Piipari, S., Barkoukis, V., & Liukkonen, J. (2017). Relationships among perceived motivational climate, motivational regulations, enjoyment, and PA participation among Finnish physical education students. International Journal of Sport & Exercise Psychology, 15(3), 273–290.

Jakobek, V., & Ljubotina, D. (2022). Povezanost ponašanja trenera, motivacijske klime i intrinzične motivacije kod mladih nogometaša. Psihologijske Teme, 31(2), 235–258. https://doi.org/10.31820/pt.31.2.2

Jerez, P., & Cabrera-Fernández, A. (2021). Clima Motivacional Percibido en El Deporte Y Su Asociación Con Los Niveles De Resiliencia en Estudiantes Universitarios. / Perceived Motivational Climate in Sport and Its Association with Resilience Levels in Undergraduate Students. Journal of Sport & Health Research, 13(3), 505–514.

Jõesaar, H., & Hein, V. (2011). Psychosocial Determinants of Young Athletes' Continued Participation over Time. Perceptual and Motor Skills, 113(1), 51–66. https://doi.org/10.2466/05.06.13.pms.113.4.51-66

Jõesaar, H., Hein, V., & Hagger, M. S. (2011). Peer influence on young athletes' need satisfaction, intrinsic motivation and persistence in sport: A 12-month prospective study. Psychology of Sport and Exercise, 12(5), 500–508. https://doi.org/10.1016/j.psychsport.2011.04.005

Johnson, C. E., Erwin, H. E., Kipp, L., & Beighle, A. (2017). Student Perceived Motivational Climate, Enjoyment, and Physical Activity in Middle School Physical Education. Journal of Teaching in Physical Education, 36(4), 398–408.

Kaprinis, S., Digelidis, N., & Papaioannou, A. (2009). Physical Education and Math: An Interdisciplinary Teaching Approach. Inquiries in Sport & Physical Education, 7(2), 90–102.

Karagiannidis, Y., Barkoukis, V., Gourgoulis, V., Kosta, G., & Antoniou, P. (2015). The role of motivation and metacognition on the development of cognitive and affective responses in physical education lessons: A self-determination approach. / O papel da motivação e metacognição no desenvolvimento das respostas afetiva e cognitiva em aulas de educação física: uma abordagem centrada na teoria da autodeterminação. Motricidade, 11(1), 135–150.

Kavussanu, M., & Roberts, G. C. (1996). Motivation in physical activity contexts: The relationship of perceived motivational climate to intrinsic motivation and self-efficacy. Journal of Sport & Exercise Psychology, 18(3), 264–280.

Kavussanu, M., White, S. A., Jowett, S., & England, S. (2011). Elite and non-elite male footballers differ in goal orientation and perceptions of parental climate. International Journal of Sport & Exercise Psychology, 9(3), 284–290.

Kelso, A., Linder, S., Reimers, A. K., Klug, S. J., Alesi, M., Scifo, L., Borrego, C. C., Monteiro, D., & Demetriou, Y. (2020). Effects of school-based interventions on motivation towards physical activity in children and adolescents: A systematic review and meta-analysis. Psychology of Sport and Exercise, 51.

Kingston, K., Wixey, D. J., & Morgan, K. (2020). Monitoring the Climate: Exploring the Psychological Environment in an Elite Soccer Academy. Journal of Applied Sport Psychology, 32(3), 297–314. https://doi.org/10.1080/10413200.2018.1481466

Kipp, L. E., & Bolter, N. D. (2020). Motivational climate, psychological needs, and personal and social responsibility in youth soccer: Comparisons by age group and competitive level. Psychology of Sport and Exercise, 51, 101756. https://doi.org/10.1016/j.psychsport.2020.101756

Kipp, L. E., & Weiss, M. R. (2013). Social influences, psychological need satisfaction, and well-being among female adolescent gymnasts. Sport, Exercise, and Performance Psychology, 2(1), 62–75. https://doi.org/10.1037/a0030236

Kipp, L. E., & Weiss, M. R. (2015). Social predictors of psychological need satisfaction and well-being among female adolescent gymnasts: A longitudinal analysis. Sport, Exercise, and Performance Psychology, 4(3), 153–169. https://doi.org/10.1037/spy0000033

Kipp, L. E., Bolter, N. D., & Phillips Reichter, A. (2019). Motivational Climate Profiles, Pubertal Status, and Well-Being Among Female Adolescent Aesthetic Sport Athletes. Pediatric Exercise Science, 31(4), 458–464. https://doi.org/10.1123/pes.2018-0182

Kipp, L., & Amorose, A. J. (2008). Perceived Motivational Climate and Self-Determined Motivation in Female High School Athletes. Journal of Sport Behavior, 31(2), 108–129.

KOLAYİŞ, H., ÇELİK, N., & NARİN, M. (2019). GENÇ YÜZÜCÜLERİN EBEVEYN GÜDÜSEL İKLİMİ İLE SPORA KATILIM MOTİVASYONLARI ARASINDAKİ İLİŞKİNİN İNCELENMESİ. / Examining the Relationship between Parent-Initiated Motivational Climate and Sport Participation Motivation of Young Swimmers. Congress Papers of The Association of Sports Sciences, 1321–1322.

Kolayi̇ş, H., Çeli̇k, N., & Nari̇n, M. (2019). GENÇ YÜZÜCÜLERİN EBEVEYN GÜDÜSEL İKLİMİ İLE SPORA KATILIM MOTİVASYONLARI ARASINDAKİ İLİŞKİNİN İNCELENMESİ. / Examining the Relationship between Parent-Initiated Motivational Climate and Sport Participation Motivation of Young Swimmers. Congress Papers of The Association of Sports Sciences, 1321–1322.

Kolayiş, H., Sarı, İ., & Çelik, N. (2017). Parent-Initiated Motivational Climate and Selfdetermined Motivation in Youth Sport: How Should Parents Behave to Keep Their Child in Sport? Kinesiology, 49(2), 217–224.

Kristiansen, E., & Roberts, G. C. (2011). Media exposure and adaptive coping in elite football. International Journal of Sport Psychology, 42(4), 339–367.

Krommidas, C., Papaioannou, A. G., Comoutos, N., Kouali, D., Galanis, E., & Chroni, S. “Ani.” (2022). Effects of parental support and coach-initiated motivational climate on young athletes’ psychosocial behaviors and well-being. Asian Journal of Sport and Exercise Psychology, 2(3), 140–150. https://doi.org/10.1016/j.ajsep.2022.06.002

Kroshus, E., & DeFreese, J. D. (2017). Athlete Burnout Prevention Strategies Used by U.S. Collegiate Soccer Coaches. The Sport Psychologist, 31(4), 332–343. https://doi.org/10.1123/tsp.2016-0067

Kuczek, P. (2013). On the Possibility of Applying Achievement Goal Theory in Competitive Sports. Human Movement, 14(2). https://doi.org/10.2478/humo-2013-0015

Langan, E., Blake, C., & Lonsdale, C. (2013). Systematic review of the effectiveness of interpersonal coach education interventions on athlete outcomes. Psychology of Sport and Exercise, 14(1), 37–49. https://doi-org.lib-e2.lib.ttu.edu/10.1016/j.psychsport.2012.06.007

Legg, E., Newland, A., & Bigelow, R. (2018). Somebody’s Eyes are Watching: The Impact of Coaching Observations on Empowering Motivational Climates and Positive Youth Development. Journal of Park and Recreation Administration, 36(4), 90–106. https://doi.org/10.18666/jpra-2018-v36-i4-8885

Lemyre, P. -N., Hall, H. K., & Roberts, G. C. (2008). A social cognitive approach to burnout in elite athletes. Scandinavian Journal of Medicine & Science in Sports, 18(2), 221–234. Portico. https://doi.org/10.1111/j.1600-0838.2007.00671.x

Lirgg, C. D., Gorman, D. R., Merrie, M. D., & Hadadi, A. A. (2022). Motivational Climate, Basic Psychological Needs, and Students’ Self-Talk in Physical Education. Physical Educator, 79(3), 280–304.

Liukkonen, J., Barkoukis, V., Watt, A., & Jaakkola, T. (2010). Motivational Climate and Students’ Emotional Experiences and Effort in Physical Education. Journal of Educational Research, 103(5), 295–308. https://doi-org.lib-e2.lib.ttu.edu/10.1080/00220670903383044

López, J. C., Álvarez, E. F., Pérez-Tejero, J., & Molinuevo, J. S. (2013). Clima motivacional, competencia percibida, compromiso y ansiedad en Educación Física Diferencias en función de la obligatoriedad de la enseñanza = Motivational climate, perceived competence, commitment and anxiety in physical education Differences by compulsory nature of education. Revista de Psicología Del Deporte, 22(1), 151–157.

Luckwu, R. M., & Guzmán, J. F. (2011). Deportividad en balonmano: Un análisis desde la Teoría de la Autodeterminación = Sportsmanship in handball: An analysis based on the self-determination theory. Revista de Psicología Del Deporte, 20(2), 305–320.

Lukwu, R. M., & Guzmán Luján, J. F. (2011). Sport commitment and adherence: A social-cognitive analysis. RICYDE. Revista Internacional de Ciencias Del Deporte / The International Journal of Sport Science, 7(25), 277–286. https://doi-org.lib-e2.lib.ttu.edu/10.5232/ricyde2011.02503

Lundqvist, C., & Raglin, J. S. (2015). The relationship of basic need satisfaction, motivational climate and personality to well-being and stress patterns among elite athletes: An explorative study. Motivation and Emotion, 39(2), 237–246. https://doi.org/10.1007/s11031-014-9444-z

Lung Hung Chen, Ying-Mei Tsai, & Ying Hwa Kee. (2007). The interaction of goal orientation and motivational climate in predicting athlete burnout. Journal of Sport & Exercise Psychology, 29, S151–S152.

MacDonald, D. J., Côté, J., Eys, M., & Deakin, J. (2011). The Role of Enjoyment and Motivational Climate in Relation to the Personal Development of Team Sport Athletes. The Sport Psychologist, 25(1), 32–46. https://doi.org/10.1123/tsp.25.1.32

Mastagli, M., Van Hoye, A., Hainaut, J.-P., & Bolmont, B. (2022). The role of an empowering motivational climate on pupils' concentration and distraction in physical education. Journal of Teaching in Physical Education, 41(2), 311–321. https://doi.org/10.1123/jtpe.2020-0252

Mavropoulou, A., Barkoukis, V., Douka, S., Alexandris, K., & Hatzimanouil, D. (2019). The role of autonomy supportive activities on students' motivation and beliefs toward out-of-school activities. Journal of Educational Research, 112(2), 223–233.

McCleery, J., Tereschenko, I., Li, L., & Copeland, N. (2023). Gender Differences in Coaching Behaviors Supportive of Positive Youth Sports Experience. Women in Sport and Physical Activity Journal, 31(2), 63–72. https://doi.org/10.1123/wspaj.2022-0024

Meaney, K. S., & Kopf, K. (2010). CPR: Promoting Cooperation, Participation and Respect in Physical Education. Strategies (08924562), 24(2), 29–32.

Melguizo-Ibáñez, E., González-Valero, G., Badicu, G., Saemi, E., & Puertas-Molero, P. (2023). Motivational Climate Effect on the Development of Anxiety and Body Image in Education Students: A Structural Equation Model. Perspectives in Psychiatric Care, 1–9. https://doi-org.lib-e2.lib.ttu.edu/10.1155/2023/2613717

Melguizo-Ibáñez, E., Zurita-Ortega, F., Ubago-Jiménez, J. L., López-Gutiérrez, C. J., & González-Valero, G. (2022). An explanatory model of the relationships between sport motivation, anxiety and physical and social self-concept in educational sciences students. Current Psychology: A Journal for Diverse Perspectives on Diverse Psychological Issues. https://doi-org.lib-e2.lib.ttu.edu/10.1007/s12144-022-02778-9

Miller, S., & Fry, M. (2018). Relationship Between Motivational Climate to Body Esteem and Social Physique Anxiety Within College Physical Activity Classes. Journal of Clinical Sport Psychology, 12(4), 525–543.

MONTEIRO, D., PELLETIER, L. G., MOUTÃO, J., & CID, L. (2018). Examining the motivational determinants of enjoyment and the intention to continue of persistent competitive swimmers. International Journal of Sport Psychology, 49(6), 484–504.

MONTEIRO, D., PELLETIER, L. G., MOUTÃO, J., & CID, L. (2018). Examining the motivational determinants of enjoyment and the intention to continue of persistent competitive swimmers. International Journal of Sport Psychology, 49(6), 484–504.

Monteiro, D., Teixeira, D. S., Travassos, B., Duarte-Mendes, P., Moutão, J., Machado, S., & Cid, L. (2018). Perceived Effort in Football Athletes: The Role of Achievement Goal Theory and Self-Determination Theory. Frontiers in Psychology, 9. https://doi.org/10.3389/fpsyg.2018.01575

Moore, E. W. G., & Fry, M. D. (2017). National franchise members' perceptions of the exercise psychosocial environment, ownership, and satisfaction. Sport, Exercise, and Performance Psychology, 6(2), 188–198.

Moore, E. W. G., & Weiller-Abels, K. (2020). Psychosocial Climates Differentially Predict 12- to 14-Year-Old Competitive Soccer Players' Goal Orientations. Women in Sport & Physical Activity Journal, 28(2), 111–118.

Mora, À., Sousa, C., & Cruz, J. (2014). El clima motivacional, la autoestima y la ansiedad en jugadores jóvenes de un club de baloncesto. Apunts Educació Física i Esports, 117, 43–50. https://doi.org/10.5672/apunts.2014-0983.es.(2014/3).117.04

Morales-Belando, M. T., Côté, J., & Arias-Estero, J. L. (2021). A longitudinal examination of the influence of winning or losing with motivational climate as a mediator on enjoyment, perceived competence, and intention to be physically active in youth basketball. Physical Education and Sport Pedagogy, 28(5), 568–581. https://doi.org/10.1080/17408989.2021.2006620

Morela, E., Hatzigeorgiadis, A., Sanchez, X., Papaioannou, A., & Elbe, A.-M. (2017). Empowering youth sport and acculturation: Examining the hosts' perspective in Greek adolescents. Psychology of Sport and Exercise, 30, 226–235. https://doi.org/10.1016/j.psychsport.2017.03.007

Morela, E., Hatzigeorgiadis, A., Theodorakis, Y., Goudas, M., & Elbe, A. (2021). Youth sport motivational climate and attitudes toward migrants' acculturation: The role of empathy and altruism. Journal of Applied Social Psychology, 51(1), 32–41. https://doi.org/10.1111/jasp.12713

Moreno Luque, M., Reigal Garrido, R. E., Morillo Baro, J. P., Morales Sánchez, V., & Hernández Mendo, A. (2019). Estilo de interacción del entrenador, clima motivacional percibido y satisfacción de las necesidades psicológicas básicas en futbolistas jóvenes. Cuadernos de Psicología Del Deporte, 19(3), 79–89. https://doi.org/10.6018/cpd.372381

Moreno Luque, M., Reigal Garrido, R. E., Morillo Baro, J. P., Morales Sánchez, V., & Hernández Mendo, A. (2019). Estilo de interacción del entrenador, clima motivacional percibido y satisfacción de las necesidades psicológicas básicas en futbolistas jóvenes. Cuadernos de Psicología Del Deporte, 19(3), 79–89. https://doi.org/10.6018/cpd.372381

Moreno Murcia, J. A. (2005). Goal Orientations, Motivational Climate, Discipline and Physical Self-Perception Related to the Teacher's Gender, Satisfaction and Sport Activity of a Sample of Spanish Adolescent Physical Education Students. International Journal of Applied Sports Sciences, 17(2), 44–58.

Moreno-Murcia, J. A., & Hernández, E. H. (2019). Effect of a teaching intervention on motivation, enjoyment, and importance given to physical education. Motricidade, 15(2–3), 21–31.

Morgan, K., & Carpenter, P. (2002). Effects of manipulating the motivational climate in physical education lessons. / Die Auswirkung der Manipulation des motivationalen Klimas in Sportunterrichtsstunden. European Physical Education Review, 8(3), 207–229.

Morris, R., & Kavussanu, M. (2008). Antecedents of approach-avoidance goals in sport. Journal of Sports Sciences, 26(5), 465–476.

Mosqueda, S., López-Walle, J. M., Gutiérrez-García, P., García-Verazaluce, J., & Tristán, J. (2019). Autonomous Motivation as a Mediator Between an Empowering Climate and Enjoyment in Male Volleyball Players. Sports, 7(6), 153. https://doi.org/10.3390/sports7060153

Mosqueda, S., Ramírez, J. J., Tomás, I., Reynaga-Estrada, P., Vanegas-Farfano, M., & López-Walle, J. M. (2022). Climas empowering y disempowering, necesidades psicológicas e intención de práctica deportiva futura en jóvenes deportistas. Revista Latinoamericana de Psicología, 54. https://doi.org/10.14349/rlp.2022.v54.7

Mosqueda, S., Ródenas-Cuenca, L. T., Balaguer, I., Salcido Otañez, Y. E., & López-Walle, J. M. (2021). Diferencias demográficas de climas motivacionales, necesidades psicológicas básicas y cohesión en jóvenes (Demographic differences in motivational climates, basic psychological needs and cohesion in young people). Retos, 43, 613–622. https://doi.org/10.47197/retos.v43i0.88608

Mudrak, J., Slepicka, P., Slepickova, I., Zabrodska, K., & Knoblochova, M. (2021). Motivational beliefs and subjective effort in adolescent athletes. International Journal of Sport Psychology, 52(4), 335–354.

Murcia, J. A. M., de San Román, M. L., Galindo, C. M., Alonso, N., & González-Cutre, D. (2008). Peers' influence on exercise enjoyment: A self-determination theory approach. Journal of Sports Science & Medicine, 7(1), 23–31.

Nerstad, C. G. L., Caniëls, M. C. J., Roberts, G. C., & Richardsen, A. M. (2020). Perceived motivational climates and employee energy: The mediating role of basic psychological needs. Frontiers in Psychology, 11. https://doi.org/10.3389/fpsyg.2020.01509

Nerstad, C. G. L., Richardsen, A. M., & Roberts, G. C. (2018). Who are the high achievers at work? Perceived motivational climate, goal orientation profiles, and work performance. Scandinavian Journal of Psychology, 59(6), 661–677.

Nerstad, C. G. L., Richardsen, A. M., & Roberts, G. C. (2018). Who are the high achievers at work? Perceived motivational climate, goal orientation profiles, and work performance. Scandinavian Journal of Psychology, 59(6), 661–677. Portico. https://doi.org/10.1111/sjop.12490

Newton, M., & Duda, J. L. (1999). The interaction of motivational climate, dispositional goal orientations, and perceived ability in predicting indices of motivation. / Interaction du climat de motivation, des buts poursuivis et des capacites percues dans la prediction des indices de motivation. International Journal of Sport Psychology, 30(1), 63–82.

Newton, M., Duda, J. L., & Yin, Z. (2000). Examination of the psychometric properties of the Perceived Motivational Climate in Sport Questionnaire - 2 in a sample of female athletes. Journal of Sports Sciences, 18(4), 275–290. https://doi.org/10.1080/026404100365018

Newton, M., Watson, D. L., Gano-Overway, L., Fry, M., Kim, M.-S., & Magyar, M. (2007). The Role of a Caring-Based Intervention in a Physical Activity Setting. The Urban Review, 39(3), 281–299. https://doi.org/10.1007/s11256-007-0065-7

Nicholls, A. R., Morley, D., & Perry, J. L. (2016). The Model of Motivational Dynamics in Sport: Resistance to Peer Influence, Behavioral Engagement and Disaffection, Dispositional Coping, and Resilience. Frontiers in Psychology, 6. https://doi.org/10.3389/fpsyg.2015.02010

Nordin-Bates, S. M., Quested, E., Walker, I. J., & Redding, E. (2012). Climate change in the dance studio: Findings from the UK centres for advanced training. Sport, Exercise, and Performance Psychology, 1(1), 3–16. https://doi.org/10.1037/a0025316

Ntoumanis, N. (2005). A Prospective Study of Participation in Optional School Physical Education Using a Self-Determination Theory Framework. Journal of Educational Psychology, 97(3), 444–453. https://doi.org/10.1037/0022-0663.97.3.444

Ntoumanis, N., & Biddle, S. (1998). The relationship between competitive anxiety, achievement goals, and motivational climates. / La relation entre l ' anxiete de la competition, la fixation des objectifs et le climat motivationnel. Research Quarterly for Exercise & Sport, 69(2), 176–187.

Ntoumanis, N., Pensgaard, A.-M., Martin, C., & Pipe, K. (2004). An Idiographic Analysis of Amotivation in Compulsory School Physical Education. Journal of Sport & Exercise Psychology, 26(2), 197–214.

Ntoumanis, N., Taylor, I. M., & Thøgersen-Ntoumani, C. (2012). A longitudinal examination of coach and peer motivational climates in youth sport: Implications for moral attitudes, well-being, and behavioral investment. Developmental Psychology, 48(1), 213–223. https://doi.org/10.1037/a0024934

Núñez, J. L., León, J., González, V., & Martín-Albo, J. (2011). Propuesta de un modelo explicativo del bienestar psicológico en el contexto deportivo = A proposal for an explanatory model of psychological well-being within the context of sport. Revista de Psicología Del Deporte, 20(1), 223–242.

O'Rourke, D. J., Smith, R. E., Punt, S., Coppel, D. B., & Breiger, D. (2017). Psychosocial correlates of young athletes' self-reported concussion symptoms during the course of recovery. Sport, Exercise, and Performance Psychology, 6(3), 262–276. https://doi.org/10.1037/spy0000097

O'Rourke, D. J., Smith, R. E., Smoll, F. L., & Cumming, S. P. (2011). Trait Anxiety in Young Athletes as a Function of Parental Pressure and Motivational Climate: Is Parental Pressure Always Harmful? Journal of Applied Sport Psychology, 23(4), 398–412. https://doi.org/10.1080/10413200.2011.552089

O'Rourke, D. J., Smith, R. E., Smoll, F. L., & Cumming, S. P. (2014). Relations of Parent- and Coach-Initiated Motivational Climates to Young Athletes' Self-Esteem, Performance Anxiety, and Autonomous Motivation: Who Is More Influential? Journal of Applied Sport Psychology, 26(4), 395–408. https://doi.org/10.1080/10413200.2014.907838

Ohlert, J., Schmitz, H., Schäfer-Pels, A., & Allroggen, M. (2022). An Empowering Climate as a Protective Factor against Sexual Violence in Sport? Social Sciences, 11(8), 330. https://doi.org/10.3390/socsci11080330

Ommundsen, Y., & Kvalø, S. E. (2007). Autonomy-Mastery, Supportive or Performance Focused? Different teacher behaviours and pupils' outcomes in physical education. Scandinavian Journal of Educational Research, 51(4), 385–413.

Ommundsen, Y., & Roberts, G. C. (1999). Effect of motivational climate profiles on motivational indices in team sport. Scandinavian Journal of Medicine & Science in Sports, 9(6), 389.

OMMUNDSEN, Y., LEMYRE, P.-N., ABRAHAMSEN, F., & ROBERTS, G. C. (2010). Motivational climate, need satisfaction, regulation of motivation and subjective vitality A study of young soccer players. International Journal of Sport Psychology, 41(3), 216–242.

Ommundsen, Y., Roberts, G. C., & Kavussanu, M. (1998). Perceived motivational climate and cognitive and affective correlates among Norwegian athletes. Journal of Sports Sciences, 16(2), 153–164. https://doi.org/10.1080/026404198366867

Omrčen, D. (2013). Knowledge production in kinesiology as viewed through the titles of papers published in an academic journal - A linguistic approach. Kinesiology, 45(1), 107–120.

Ortega, F. Z., Zafra Santos, E. O., Moral, P. V., Fernández, S. R., Sánchez, M. C., & Muros Molina, J. J. (2017). Análisis de la resiliencia, autoconcepto y motivación en judo según el género = Analysis of resilience, self-concept and motivation in judo as gender. Revista de Psicología Del Deporte, 26(1), 71–81.

Pacewicz, C. E., Smith, A. L., & Raedeke, T. D. (2020). Group cohesion and relatedness as predictors of self-determined motivation and burnout in adolescent female athletes. Psychology of Sport and Exercise, 50, 101709. https://doi.org/10.1016/j.psychsport.2020.101709

Papaioannou, A. G., Ampatzoglou, G., Kalogiannis, P., & Sagovits, A. (2008). Social agents, achievement goals, satisfaction and academic achievement in youth sport. Psychology of Sport and Exercise, 9(2), 122–141. https://doi.org/10.1016/j.psychsport.2007.02.003

Papaioannou, A. G., Tsigilis, N., Kosmidou, E., & Milosis, D. (2007). Measuring perceived motivational climate in physical education. Journal of Teaching in Physical Education, 26(3), 236–259. https://doi.org./10.1123/jtpe.26.3.236

Papaioannou, A., & Kouli, O. (1999). The effect of task structure, perceived motivational climate and goal orientations on students' task involvement and anxiety. Journal of Applied Sport Psychology, 11(1), 51–71. https://doi-org.lib-e2.lib.ttu.edu/10.1080/10413209908402950

Papioannou, A. (1995). Differential perceptual and motivational patterns when different goals are adopted. Journal of Sport & Exercise Psychology, 17(1), 18–34.

Parental climate in sports. (1999). Journal of Sport & Exercise Psychology, 21(2), 184.

Pensgaard, A. M. (1999). The dynamics of motivation and perceptions of control when competing in the Olympic Games. Perceptual and Motor Skills, 89(1), 116–125. https://doi.org.lib.ttu.edu/10.2466/PMS.89.5.116-125

Pensgaard, A. M., & Roberts, G. C. (2000). The relationship between motivational climate, perceived ability and sources of distress among elite athletes. Journal of Sports Sciences, 18(3), 191–200.

Pensgaard, A. M., Roberts, G. C., & Ursin, H. (1999). Motivational Factors and Coping Strategies of Norwegian Paralympic and Olympic Winter Sport Athletes. Adapted Physical Activity Quarterly, 16(3), 238–250. https://doi.org/10.1123/apaq.16.3.238

Pfeffer, I., & Gallitschke, M. (2008). Trainerinnen und Trainer im Frauenfußball aus Sicht der Athletinnen. Zeitschrift Für Sportpsychologie, 15(3), 88–95. https://doi.org/10.1026/1612-5010.15.3.88

Pfeffer, I., Wuerth, S., & Alfermann, D. (2004). Die subjektive Wahrnehmung der Trainer-Athlet-Interaktion in Individualsportarten und Mannschaftsspielen. / Subjective perception of the coach-athlete interaction in individual and team sports. German Journal of Sport Psychology / Zeitschrift Für Sportpsychologie, 11(1), 24–32.

Pineda-Espejel, A., López-Walle, J., & Tomás, I. (2015). Factores situacionales y disposicionales como predictores de la ansiedad y autoconfianza precompetitiva en deportistas universitarios. Cuadernos de Psicología Del Deporte, 15(2), 55–70. https://doi.org/10.4321/s1578-84232015000200007

Pineda-Espejel, H. A., Alarcón, E., Morquecho-Sánchez, R., Morales-Sánchez, V., & Gadea-Cavazos, E. (2021). Adaptive social factors and precompetitive anxiety in elite sport. Frontiers in Psychology, 12. https://doi.org/10.3389/fpsyg.2021.651169

Pineda-Espejel, H. A., Alarcón, E., Morquecho-Sánchez, R., Morales-Sánchez, V., & Gadea-Cavazos, E. (2021). Adaptive Social Factors and Precompetitive Anxiety in Elite Sport. Frontiers in Psychology, 12. https://doi.org/10.3389/fpsyg.2021.651169

Pitsi, A., Digelidis, N., & Papaioannou, A. (2015). The effects of reciprocal and self-check teaching styles in students' intrinsic-extrinsic motivation, enjoyment and autonomy in teaching traditional Greek dances. Journal of Physical Education & Sport, 15(2), 352–361.

Quested, E., & Duda, J. L. (2009). Perceptions of the Motivational Climate, Need Satisfaction, and Indices of Well- and Ill-Being among Hip Hop Dancers. Journal of Dance Medicine & Science, 13(1), 10–19. https://doi.org/10.1177/1089313x0901300102

Quested, E., & Duda, J. L. (2010). Exploring the Social-Environmental Determinants of Well- and Ill-Being in Dancers: A Test of Basic Needs Theory. Journal of Sport and Exercise Psychology, 32(1), 39–60. https://doi.org/10.1123/jsep.32.1.39

Quested, E., & Duda, J. L. (2010). Exploring the Social-Environmental Determinants of Well- and Ill-Being in Dancers: A Test of Basic Needs Theory. Journal of Sport and Exercise Psychology, 32(1), 39–60. https://doi.org/10.1123/jsep.32.1.39

Quintas, A., & Bustamante, J.-C. (2021). Effects of gamified didactic with exergames on the psychological variables associated with promoting physical exercise: results of a natural experiment run in primary schools. Physical Education and Sport Pedagogy, 28(5), 467–481. https://doi.org/10.1080/17408989.2021.1991905

Raimundi, M. J., Corti, J. F., Pérez-Gaido, M., Alvarez, O., & Castillo, I. (2023). Which Assessment of Coach-Created Motivational Climate Better Predicts Young Athletes' Engagement over a Season? Athletes' Perceptions and Match Observations Do. Sustainability, 15(6), 5179. https://doi.org/10.3390/su15065179

Raimundi, M. J., Pérez-Gaido, M., Celsi, I., & Castillo, I. (2022). Evaluación de los Estilos Interpersonales y Climas Motivacionales Creados por Entrenadores en Deporte Adolescente Argentino = Evaluation of interpersonal styles and motivational climates created by coaches in Argentinean adolescent sport. Revista Iberoamericana de Diagnóstico y Evaluación Psicologica, 65(4), 163–178. https://doi-org.lib-e2.lib.ttu.edu/10.21865/RIDEP65.4.12

Reinboth, M., & Duda, J. L. (2004). The Motivational Climate, Perceived Ability, and Athletes' Psychological and Physical Well-Being. The Sport Psychologist, 18(3), 237–251. https://doi.org/10.1123/tsp.18.3.237

Reinboth, M., & Duda, J. L. (2006). Perceived motivational climate, need satisfaction and indices of well-being in team sports: A longitudinal perspective. Psychology of Sport and Exercise, 7(3), 269–286. https://doi.org/10.1016/j.psychsport.2005.06.002

Reinboth, M., & Duda, J. L. (2016). Effects of competitive environment and outcome on achievement behaviors and well-being while engaged in a physical task. Sport, Exercise, and Performance Psychology, 5(4), 324–336. https://doi.org/10.1037/spy0000075

Reinboth, M., Duda, J. L., & Ntoumanis, N. (2004). Dimensions of Coaching Behavior, Need Satisfaction, and the Psychological and Physical Welfare of Young Athletes. Motivation and Emotion, 28(3), 297–313. https://doi.org/10.1023/b:moem.0000040156.81924.b8

Reverberi, E., D'Angelo, C., Littlewood, M. A., & Gozzoli, C. F. (2020). Youth Football Players' Psychological Well-Being: The Key Role of Relationships. Frontiers in Psychology, 11. https://doi.org/10.3389/fpsyg.2020.567776

Robazza, C., Morano, M., Bortoli, L., & Ruiz, M. C. (2022). Perceived motivational climate influences athletes' emotion regulation strategies, emotions, and psychobiosocial experiences. Psychology of Sport and Exercise, 59, 102110. https://doi.org/10.1016/j.psychsport.2021.102110

Robazza, C., Ruiz, M. C., & Bortoli, L. (2021). Psychobiosocial experiences in sport: Development and initial validation of a semantic differential scale. Psychology of Sport and Exercise, 55, 101963. https://doi.org/10.1016/j.psychsport.2021.101963

Robinson, L. E., & Goodway, J. D. (2009). Instructional Climates in Preschool Children Who Are At-Risk. Part I. Research Quarterly for Exercise and Sport, 80(3), 533–542. https://doi.org/10.1080/02701367.2009.10599591
Rodrigues Alves, M. A., Oliveira, A. dos S., Paes, M. J., & Facco Stefanello, J. M. (2022). Psychological aspects of soccer and futsal players: A systematic review. Suma Psicológica, 29(1), 30–47.
Rodrigues, F., Macedo, R., Cid, L., Teixeira, D. S., Marinho, D. A., & Monteiro, D. (2020). Sex differences in relationships between perceived coach-induced motivational climates, basic psychological needs, and behavior regulation among young swimmers. Perceptual and Motor Skills, 127(5), 891–911. https://doi.org/10.1177/0031512520926805
Rudisill, M. E. (2016). Mastery Motivational Climates: Motivating Children to Move and Learn in Physical Education Contexts. Kinesiology Review, 5(3), 157–169.
Ruiz-Juan, F., Baños, R., Fuentesal-Garcia, J., García-Montes, E., & Baena-Extremera, A. (2019). ANÁLISIS TRANSCULTURAL DEL CLIMA MOTIVACIONAL EN ALUMNADO DE COSTA RICA, MÉXICO Y ESPAÑA. Revista Internacional de Medicina y Ciencias de La Actividad Física y Del Deporte, 19(74). https://doi.org/10.15366/rimcafd2019.74.011
Ruiz, M. C., Appleton, P. R., Duda, J. L., Bortoli, L., & Robazza, C. (2021). Social Environmental Antecedents of Athletes' Emotions. International Journal of Environmental Research and Public Health, 18(9), 4997. https://doi.org/10.3390/ijerph18094997
Ruiz, M. C., Haapanen, S., Tolvanen, A., Robazza, C., & Duda, J. L. (2017). Predicting athletes' functional and dysfunctional emotions: The role of the motivational climate and motivation regulations. Journal of Sports Sciences, 1–9. https://doi.org/10.1080/02640414.2016.1225975
Ruiz, M. C., Robazza, C., Tolvanen, A., Haapanen, S., & Duda, J. L. (2019). Coach-Created Motivational Climate and Athletes' Adaptation to Psychological Stress: Temporal Motivation-Emotion Interplay. Frontiers in Psychology, 10. https://doi.org/10.3389/fpsyg.2019.00617
Russell, W. D. (2014). The Relationship between Youth Sport Specialization, Reasons for Participation, and Youth Sport Participation Motivations: A Retrospective Study. Journal of Sport Behavior, 37(3), 286–305.
Salselas, V., & Márquez, S. (2009). Perceptions of the Motivational Climate Created by Parents of Young Portuguese Swimmers. Perceptual and Motor Skills, 108(3), 851–861. https://doi.org/10.2466/pms.108.3.851-861

Sánchez-Miguel, P. A., Leo, F. M., Sánchez-Oliva, D., Amado, D., & García-Calvo, T. (2013). The Importance of Parents' Behavior in their Children's Enjoyment and Amotivation in Sports. Journal of Human Kinetics, 36(1), 169–177. https://doi.org/10.2478/hukin-2013-0017

Sánchez-Oliva, D., Leo-Marcos, F. M., Sánchez-Miguel, P. A., Amado-Alonso, D., & García-Calvo, T. (2010). Relación del clima motivacional creado por el entrenador con la motivación autodeterminada y la implicación hacia la práctica deportiva. (Relationship between motivational climate created by coach regarding self-determined motivation and the involvement through the practice). RICYDE. Revista Internacional de Ciencias Del Deporte, 6(20), 177–195. https://doi.org/10.5232/ricyde2010.02001

Sánchez-Oliva, D., Leo, F. M., González-Ponce, I., Chamorro, J. M., & García-Calvo, T. (2012). Analizando la implicación deportiva en jóvenes jugadores de baloncesto: Un análisis desde la Teoría de la Autodeterminación. / Examining sport involvement in youth basketball players: an analysis from self-determination theory. Cuadernos de Psicología Del Deporte, 12(S1), 57–61.

Sánchez, M., Sánchez-Sánchez, J., Carcedo, R. J., & García, J. A. (2020). Climate, Orientation and Fun in Under-12 Soccer Players. / Clima, Orientación Motivacional Y Diversión en Futbolistas Alevines. International Journal of Medicine & Science of Physical Activity & Sport / Revista Internacional de Medicina y Ciencias de La Actividad Física y Del Deporte, 20(79), 507–520.

Santos-Rosa, F. J., Montero-Carretero, C., Gómez-Landero, L. A., Torregrossa, M., & Cervelló, E. (2022). Positive and negative spontaneous self-talk and performance in gymnastics: The role of contextual, personal and situational factors. PLOS ONE, 17(3), e0265809. https://doi.org/10.1371/journal.pone.0265809

Sarı, İ., & Bizan, İ. (2022). The role of parent initiated motivational climate in athletes' engagement and dispositional flow. Kinesiology, 54(1), 3–4. https://doi.org/10.26582/k.54.1.1

Sari, İ., & Deryahanoğlu, G. (2019). Sporcularda Ahlaktan Uzaklaşmanin Güdüsel İkli̇m Ve Sporcularin Önem Verdi̇kleri̇ Özelli̇kler İle İli̇şki̇si̇. / the Relationship of Athletes' Moral Disengagement to Perceived Motivational Climate and Valued Goals. SPORMETRE: The Journal of Physical Education & Sport Sciences / Beden Eğitimi ve Spor Bilimleri Dergisi, 17(4), 91–104.

Scott, C. E., Fry, M. D., Weingartner, H., & Wineinger, T. O. (2021). Collegiate Sport Club Athletes' Psychological Well-Being and Perceptions of Their Team Climate. Recreational Sports Journal, 45(1), 17–26. https://doi.org/10.1177/1558866121995169

Scott, C. E., Wineinger, T. O., Iwasaki, S., & Fry, M. D. (2021b). Creating an Optimal Motivational Team Climate to Help Collegiate Athletes Thrive during the COVID-19 Pandemic. Journal of Sport Psychology in Action, 12(2), 127–141. https://doi.org/10.1080/21520704.2021.1876194

Selfriz, J. J., Duda, J. L., & Chi, L. (1992). The Relationship of Perceived Motivational Climate to Intrinsic Motivation and Beliefs about Success in Basketball. Journal of Sport and Exercise Psychology, 14(4), 375–391. https://doi.org/10.1123/jsep.14.4.375

Sevil, J., Abós, Á., Aibar, A., Julián, J. A., & García-González, L. (2016). Gender and corporal expression activity in physical education. European Physical Education Review, 22(3), 372–389.

SGRÒ, F., BARCA, M., SCHEMBRI, R., & LIPOMA, M. (2020). Assessing the effect of different teaching strategies on students' affective learning outcomes during volleyball lessons. Journal of Physical Education & Sport, 20, 2136–2142.

Sgrò, F., Barca, M., Schembri, R., & Lipoma, M. (2020). Assessing the effect of different teaching strategies on students' affective learning outcomes during volleyball lessons. Journal of Physical Education & Sport, 20, 2136–2142.

Sheehan, R. B., Herring, M. P., & Campbell, M. J. (2018). Longitudinal relations of mental health and motivation among elite student-athletes across a condensed season: Plausible influence of academic and athletic schedule. Psychology of Sport and Exercise, 37, 146–152. https://doi.org/10.1016/j.psychsport.2018.03.005

Sheehan, R. B., Herring, M. P., & Campbell, M. J. (2018a). Longitudinal relations of mental health and motivation among elite student-athletes across a condensed season: Plausible influence of academic and athletic schedule. Psychology of Sport and Exercise, 37, 146–152. https://doi.org/10.1016/j.psychsport.2018.03.005

Sheehan, R. B., Herring, M. P., & Campbell, M. J. (2018b). Associations Between Motivation and Mental Health in Sport: A Test of the Hierarchical Model of Intrinsic and Extrinsic Motivation. Frontiers in Psychology, 9. https://doi.org/10.3389/fpsyg.2018.00707

Shipherd, A. M., Wakefield, J. C., Stokowski, S., & Filho, E. (2019). The influence of coach turnover on student-athletes' affective states and team dynamics: An exploratory study in collegiate sports. International Journal of Sports Science & Coaching, 14(1), 97–106.

Smith, A. L., Balaguer, I., & Duda, J. L. (2006). Goal orientation profile differences on perceived motivational climate, perceived peer relationships, and motivation-related responses of youth athletes. Journal of Sports Sciences, 24(12), 1315–1327. https://doi.org/10.1080/02640410500520427

Smith, A. L., Gustafsson, H., & Hassmén, P. (2010). Peer motivational climate and burnout perceptions of adolescent athletes. Psychology of Sport and Exercise, 11(6), 453–460. https://doi.org/10.1016/j.psychsport.2010.05.007

Smith, N., Tessier, D., Tzioumakis, Y., Quested, E., Appleton, P., Sarrazin, P., Papaioannou, A., & Duda, J. L. (2015). Development and Validation of the Multidimensional Motivational Climate Observation System. Journal of Sport and Exercise Psychology, 37(1), 4–22. https://doi.org/10.1123/jsep.2014-0059

Smith, R. E., Smoll, F. L., & Cumming, S. P. (2007). Effects of a motivational climate inntervention for coaches on young athletes' sport performance anxiety. Journal of Sport & Exercise Psychology, 29(1), 39–59.

Smith, R., Cumming, S., & Smoll, F. (2008). Development and Validation of the Motivational Climate Scale for Youth Sports. Journal of Applied Sport Psychology, 20(1), 116–136.

Smith, Ronald E., Frank L. Smoll, Sean P. Cumming, and Joel R. Grossbard. 2006. "Measurement of Multidimensional Sport Performance Anxiety in Children and Adults: The Sport Anxiety Scale-2." Journal of Sport & Exercise Psychology 28 (4): 479–501. doi:10.1123/jsep.28.4.479.

Sokoloff, D. M., Petrie, T. A., & Chu, T. L. (Alan). (2023). The Relationship of Coach-Created Motivational Climate to Teamwork Behaviors in Female Collegiate Athletes. The Sport Psychologist, 37(1), 40–47. https://doi.org/10.1123/tsp.2022-0070

Solmon, M. A. (2015). Optimizing the Role of Physical Education in Promoting Physical Activity: A Social-Ecological Approach. Research Quarterly for Exercise and Sport, 86(4), 329–337. https://doi.org/10.1080/02701367.2015.1091712

Solstad, B. E., & Lemyre, P.-N. (2014). Why Coaches should Encourage Swimmers' Efforts to Succeed. Journal of Swimming Research, 22(1), 1–9.

Solstad, B. E., Ivarsson, A., Haug, E. M., & Ommundsen, Y. (2018). Youth Sport Coaches' Well-Being Across the Season: The Psychological Costs and Benefits of Giving Empowering and Disempowering Sports Coaching to Athletes. International Sport Coaching Journal, 5(2), 124–135. https://doi.org/10.1123/iscj.2017-0026

Spittle, M., & Byrne, K. (2009). The influence of Sport Education on student motivation in physical education. Physical Education & Sport Pedagogy, 14(3), 253–266.

Spray, C. M. (2000). Predicting participation in noncompulsory physical education: Do goal perspectives matter. Perceptual and Motor Skills, 90(3,Pt2), 1207–1215. https://doi-org.lib-e2.lib.ttu.edu/10.2466/PMS.90.3.1207-1215

Stark, A., & Newton, M. (2014). A dancer's well-being: The influence of the social psychological climate during adolescence. Psychology of Sport and Exercise, 15(4), 356–363. https://doi.org/10.1016/j.psychsport.2014.03.003

Steffen, K., Pensgaard, A. M., & Bahr, R. (2009). Self-reported psychological characteristics as risk factors for injuries in female youth football. Scandinavian Journal of Medicine & Science in Sports, 19(3), 442–451.
Sukys, S., Kromerova-Dubinskiene, E., & Appleton, P. R. (2020). Validation of the Lithuanian Version of the Coach-Created Empowering and Disempowering Motivational Climate Questionnaire (EDMCQ-C). International Journal of Environmental Research and Public Health, 17(10), 3487. https://doi.org/10.3390/ijerph17103487
Sullivan, G. S., & Strode, J. P. (2010). Motivation through Goal Setting: A Self-Determined Perspective. Strategies: A Journal for Physical and Sport Educators, 23(6), 19–23.
Tamminen, K. A., Gaudreau, P., McEwen, C. E., & Crocker, P. R. E. (2016). Interpersonal Emotion Regulation Among Adolescent Athletes: A Bayesian Multilevel Model Predicting Sport Enjoyment and Commitment. Journal of Sport and Exercise Psychology, 38(6), 541–555. https://doi.org/10.1123/jsep.2015-0189
Task-involving climate is tops. (1999). Journal of Sport & Exercise Psychology, 21(2), 182.
Taylor, I. M., & Ntoumanis, N. (2007). Teacher motivational strategies and student self-determination in physical education. Journal of Educational Psychology, 99(4), 747–760. https://doi-org.lib-e2.lib.ttu.edu/10.1037/0022-0663.99.4.747
Taylor, I. M., Spray, C. M., & Pearson, N. (2014). The Influence of the Physical Education Environment on Children's Well-Being and Physical Activity Across the Transition from Primary to Secondary School. Journal of Sport and Exercise Psychology, 36(6), 574–583. https://doi.org/10.1123/jsep.2014-0038
The Digest for October 2012. (2012). Journal of Sport & Exercise Psychology, 34(5), 683–691.
The Digest. (2014). Journal of Sport & Exercise Psychology, 36(3), 321–326.
Theeboom, M., & De Knop, P. (1995). Motivational Climate, Psychological Responses, and Motor Skill Development in Children's Sport: A Field-Based Intervention Study. Journal of Sport & Exercise Psychology, 17(3), 294–311.
Theodosiou, A., & Papaioannou, A. (2006). Motivational climate, achievement goals and metacognitive activity in physical education and exercise involvement in out-of-school settings. Psychology of Sport and Exercise, 7(4), 361–379.

THEODOSIOU, A., GERANI, C., BARKOUKIS, V., TSIGILIS, N., DRAKOU, A., WILLIAMS, S., KERSHAW, A., & ROSE, M. (2021). Motivation and well-being in school physical education A cross-national study of self determination theory. International Journal of Sport Psychology, 52(3), 213–232.

Thrower, S. N., Spray, C. M., & Harwood, C. G. (2023). Evaluating the “Optimal Competition Parenting Workshop” Using the RE-AIM Framework: A 4-Year Organizational-Level Intervention in British Junior Tennis. Journal of Sport & Exercise Psychology, 45(1), 1–14. https://doi.org/10.1123/jsep.2022-0080

Torregrosa, M., Sousa, C., Viladrich, C., Villamarín, F., & Cruz, J. (2008). El clima motivational y el estilo de comunicación del entrenador como predictores del compromiso en futbolistas jóvenes = Motivational climate and coaches’ communication style predict young soccer players’ commitment. Psicothema, 20(2), 254–259.

Torregrosa, M., Viladrich, C., Ramis, Y., Azócar, F., Latinjak, A. T., & Cruz, J. (2011). Efectos en la percepción del clima motivacional generado por los entrenadores y compañeros sobre la diversión y el compromiso Diferencias en función de género = Effects on the perception of the motivational climate created by coaches and teammates on enjoyment and commitment Gender differences. Revista de Psicología Del Deporte, 20(1), 243–255.

Travers, L. V., Bohnert, A. M., & Randall, E. T. (2013). Brief report: Adolescent adjustment in affluent communities: The role of motivational climate and goal orientation. Journal of Adolescence, 36(2), 423–428. https://doi.org./10.1016/j.adolescence.2012.11.009

Trbojević Jocić, J., & Petrović, J. (2021). Understanding of dropping out of sports in adolescence. Kinesiology, 53(2), 245–256. https://doi.org/10.26582/k.53.2.7

Trbojević Jocić, J., & Petrović, J. (2021). Understanding of dropping out of sports in adolescence. Kinesiology, 53(2), 245–256. https://doi.org/10.26582/k.53.2.7

Trbojević, J., Mandarić, S., & Petrović, J. (2020). Perceived motivational climate created by coach and physical self-efficacy as predictors of the young Serbian female athletes satisfaction. Fizicka Kultura, 74(2), 173–183. https://doi.org/10.5937/fizkul74-28098

Treasure, D. C. (1997). Perceptions of the Motivational Climate and Elementary School Children’s Cognitive and Affective Response. Journal of Sport and Exercise Psychology, 19(3), 278–290. https://doi.org/10.1123/jsep.19.3.278

Treasure, D. C., & Robert, G. C. (2001). Students’ Perceptions of the Motivational Climate, Achievement Beliefs, and Satisfaction in Physical Education. Research Quarterly for Exercise and Sport, 72(2), 165–175. https://doi.org/10.1080/02701367.2001.10608946

Treasure, D. C., & Roberts, G. C. (1998). Relationship between female adolescents’ achievement goal orientations, perceptions of the motivational climate, belief about success and sources of satisfaction in basketball. / Relation entre les buts de reussite, les perceptions du climat motivationnel, les espoirs de reussite et les sources de satisfaction chez des adolescentes pratiquant le basketball. International Journal of Sport Psychology, 293, 211–230.

Trenz, R. C., & Zusho, A. (2011). Competitive Swimmers’ Perception of Motivational Climate and Their Personal Achievement Goals. International Journal of Sports Science & Coaching, 6(3), 433–443. https://doi.org/10.1260/1747-9541.6.3.433

Troncoso Avalos, S. M., Burgos Dávila, C. J., & López-Walle, J. M. (2015). Climas motivacionales, liderazgo y cohesión grupal en contexto deportivo universitario. / Motivational atmosphere, leadership and group cohesion in university sports context. Educación Física y Ciencia, 17(1), 1–12.

Tudor, M., & Ridpath, B. D. (2019). Does Gender Significantly Predict Academic, Athletic Career Motivation among NCAA Division I College Athletes. Journal of Higher Education Athletics & Innovation, 5, 122–147. https://doi.org/10.15763/issn.2376-5267.2018.1.5.122-147

Ullrich-French, S., & Cox, A. E. (2014). Normative and intraindividual changes in physical education motivation across the transition to middle school: A multilevel growth analysis. Sport, Exercise, and Performance Psychology, 3(2), 132–147. https://doi-org.lib-e2.lib.ttu.edu/10.1037/spy0000005

Valero, A., Delgado, M., & Conde, J. L. (2009). Motivación hacia la práctica del atletismo en la educación primaria en función de dos propuestas de enseñanza/aprendizaje = Motivation towards athletics practice in primary education depending on two different teaching/learning proposals. Revista de Psicología Del Deporte, 18(2), 123–136.

van de Pol, P. K. C., Kavussanu, M., & Ring, C. (2012). Goal orientations, perceived motivational climate, and motivational outcomes in football: A comparison between training and competition contexts. Psychology of Sport & Exercise, 13(4), 491–499.

Vaquero-Cristóbal, R., Abenza-Cano, L., Albaladejo-Saura, M., Meroño, L., Marcos-Pardo, P. J., Esparza-Ros, F., & González-Gálvez, N. (2021). Influence of an educational innovation program and digitally supported tasks on psychological aspects, motivational climate, and academic performance. Education Sciences, 11(12), 1–12. https://doi-org.lib-e2.lib.ttu.edu/10.3390/educsci11120821

Vasconcelos-Raposo, J., Carvalho, R., & Teixeira, C. (2012). Perceção de competência atlética em jovens praticantes de Futebol: Efeitos da posição ocupada no campo, da participação em competição, do tempo e frequência de prática e do clima motivacional... / Perceived athletic competence in young football players: Effects of the position occupied in the field, participation in competition, time and frequency of practice and motivational climate induced by parents. Revista Portuguesa de Ciências Do Desporto, 12(2), 58–70.

Vazou, S., Mischo, A., Ladwig, M. A., Ekkekakis, P., & Welk, G. (2019). Psychologically informed physical fitness practice in schools: A field experiment. Psychology of Sport and Exercise, 40, 143–151. https://doi.org/10.1016/j.psychsport.2018.10.008

Vazou, S., Ntoumanis, N., & Duda, J. L. (2006). Predicting young athletes' motivational indices as a function of their perceptions of the coach- and peer-created climate. Psychology of Sport and Exercise, 7(2), 215–233. https://doi-org.lib-e2.lib.ttu.edu/10.1016/j.psychsport.2005.08.007

Vitali, F., Bortoli, L., Bertinato, L., Robazza, C., & Schena, F. (2015). Motivational climate, resilience, and burnout in youth sport. Sport Sciences for Health, 11(1), 103–108. https://doi.org/10.1007/s11332-014-0214-9

Vosloo, J., Ostrow, A., & Watson, J. C. (2009). The Relationships Between Motivational Climate, Goal Orientations, Anxiety, and Self-Confidence Among Swimmers. Journal of Sport Behavior, 32(3), 376–393.

Wagnsson, S., Stenling, A., Gustafsson, H., & Augustsson, C. (2016). Swedish youth football players' attitudes towards moral decision in sport as predicted by the parent-initiated motivational climate. Psychology of Sport & Exercise, 25, 110–114.

Walker, I. J., Nordin-Bates, S. M., & Redding, E. (2011). Characteristics of talented dancers and age group differences: findings from the UK Centres for Advanced Training. High Ability Studies, 22(1), 43–60. https://doi.org/10.1080/13598139.2011.597587

Wallhead, T. L., & Ntoumanis, N. (2004). Effects of a Sport Education Intervention on Students' Motivational Responses in Physical Education. Journal of Teaching in Physical Education, 23(1), 4–18.

Walling, M. D., Duda, J. L., & Chi, L. (1993). The Perceived Motivational Climate in Sport Questionnaire: Construct and Predictive Validity. Journal of Sport and Exercise Psychology, 15(2), 172–183. https://doi.org/10.1123/jsep.15.2.172

Wang, J. C. K., Woon Chia Liu, Chatzisarantis, N. L. D., & Lim, C. B. S. (2010). Influence of Perceived Motivational Climate on Achievement Goals in Physical Education: A Structural Equation Mixture Modeling Analysis. Journal of Sport & Exercise Psychology, 32(3), 324–338.

Webb, E., & Forrester, S. (2016). Peer Motivational Climate and Its Relationship with Positive and Negative Affect in Collegiate Intramural Sports. International Journal of Sport Management, 17(4), 622–645.

Weeldenburg, G., Borghouts, L. B., Slingerland, M., & Vos, S. (2020). Similar but different: Profiling secondary school students based on their perceived motivational climate and psychological need-based experiences in physical education. PLoS ONE, 15(2). https://doi-org.lib-e2.lib.ttu.edu/10.1371/journal.pone.0228859

Weigand, D. A. (2001). Introduction to the Special Issue on Motivational Climate in Sport and Physical Education. European Journal of Sport Science, 1(4), 1.

Weigand, D. A. (2001). Introduction to the Special Issue on Motivational Climate in Sport and Physical Education. European Journal of Sport Science, 1(4), 1.

Weigand, D., & Burton, S. (2002). Manipulating achievement motivation in physical education by manipulating the motivational climate. European Journal of Sport Science, 2(1), 1–14. https://doi.org/10.1080/17461390200072102

Weiss, M. R., Amorose, A. J., & Wilko, A. M. (2009). Coaching Behaviors, Motivational Climate, and Psychosocial Outcomes among Female Adolescent Athletes. Pediatric Exercise Science, 21(4), 475–492. https://doi.org/10.1123/pes.21.4.475

Weiss, W. M. (2014). Competitive-level differences on sport commitment among high school- and collegiate-level athletes. International Journal of Sport and Exercise Psychology, 13(3), 286–303. https://doi.org/10.1080/1612197x.2014.958517

White, S. A. (1998). Adolescent goal profiles, perceptions of the parent-initiated motivational climate, and competitive trait anxiety. The Sport Psychologist, 12(1), 16–28.

Whitehead, J., Andrée, K. V., & Lee, M. J. (2004). Achievement perspectives and perceived ability: how far do interactions generalize in youth sport? Psychology of Sport and Exercise, 5(3), 291–317. https://doi.org/10.1016/s1469-0292(03)00016-5

Wilhelmsen, T., Sørensen, M., & Seippel, Ø. N. (2019). Motivational Pathways to Social and Pedagogical Inclusion in Physical Education. Adapted Physical Activity Quarterly, 36(1), 19–41. https://doi.org/10.1123/apaq.2018-0019

Williams, L. (1998). Contextual influences and goal perspectives among female youth sport participants. / Influences contextuelles et perspectives d ′ objectifs chez des jeunes femmes sportives. Research Quarterly for Exercise & Sport, 69(1), 47–57.

Williams, R. E., Habeeb, C. M., Raedeke, T. D., Dlugonski, D., & Dubose, K. D. (2022). Parent Motivational Climate, Sport Enrollment Motives, and Young Athlete Commitment and Enjoyment in Year-Round Swimming. International Journal of Exercise Science, 15(5), 358–372.

Woods, S., Dunne, S., & Gallagher, P. (2023). Examining the Utility of Stress-, Motivation-, and Commitment-Based Perspectives of Athlete Burnout. Journal of Sport & Exercise Psychology, 45(5), 257–268. https://doi.org/10.1123/jsep.2022-0127

Wu, X., Zainal Abidin, N. E., & Aga Mohd Jaladin, R. (2021). Motivational Processes Influencing Mental Health Among Winter Sports Athletes in China. Frontiers in Psychology, 12. https://doi.org/10.3389/fpsyg.2021.726072

Wuerth, S., Saborowski, C., & Alfermann, D. (1999). Trainingsklima und Fuehrungsverhalten aus der Sicht jugendlicher Athleten und deren Trainer. / Motivational climate and leadership behavior as perceived by young athletes and their coaches. Psychologie Und Sport, 6(4), 146–157.

Zach, S., Raviv, T., & Meckel, Y. (2016). Using information communication technologies (ICTs) for motivating female adolescents to exercise/run in their leisure time. Computers in Human Behavior, 60, 593–601. https://doi.org/10.1016/j.chb.2016.02.096

Zanatta, T., Rottensteiner, C., Konttinen, N., & Lochbaum, M. (2018). Individual Motivations, Motivational Climate, Enjoyment, and Physical Competence Perceptions in Finnish Team Sport Athletes: A Prospective and Retrospective Study. Sports, 6(4), 165. https://doi.org/10.3390/sports6040165

ZHANG Haijun, GUO Xiaotao, & CHEN Bo. (2015). Effects of Coaches' Paternalistic Leadership Behaviors on Psychological Well-Being in College Athletes: From Self-Determination Theory Perspective. Journal of Wuhan Institute of Physical Education, 49(6), 82–88.

Zoltán, K., Barbara, F., István, C., & József, B. (2015). Bentlakó labdarúgó-korosztályok pszichés-mentális jellemzői = Psychological and mental characteristics of young football players living in dorms. Mentálhigiéné És Pszichoszomatika, 16(4), 331–347. https://doi.org/10.1556/0406.16.2015.4.2

Supplement Table 3. Correlates entered for each study.

Study	Climate	r	N	Correlate description
Abrahamsen & Kristiansen 2015	Mastery climate 1	0.64	27	Anxiety somatic
Abrahamsen et al. 2008a	Mastery climate 1	0.13	101	Anxiety somatic
Abrahamsen et al. 2008a	Mastery climate 2	-0.14	101	Anxiety cognitive
Abrahamsen et al. 2008a	Mastery climate 3	-0.04	101	Anxiety somatic
Abrahamsen et al. 2008a	Mastery climate 4	-0.14	101	Anxiety cognitive
Abrahamsen et al. 2008a	Mastery climate 5	-0.16	101	Anxiety concentration disruption
Abrahamsen et al. 2008a	Mastery climate 6	-0.03	101	Anxiety concentration disruption
Abrahamsen et al. 2008a	Performance climate 1	0.15	89	Anxiety somatic
Abrahamsen et al. 2008a	Performance climate 2	0.31	89	Anxiety cognitive
Abrahamsen et al. 2008a	Performance climate 3	0.03	89	Anxiety somatic
Abrahamsen et al. 2008a	Performance climate 4	0.39	89	Anxiety cognitive
Abrahamsen et al. 2008a	Performance climate 5	0.25	89	Anxiety concentration disruption
Abrahamsen et al. 2008a	Performance climate 6	0.13	89	Anxiety concentration disruption
Abrahamsen et al. 2008b	Mastery climate 1	-0.18	143	Anxiety somatic
Abrahamsen et al. 2008b	Mastery climate 2	-0.01	143	Anxiety worry
Abrahamsen et al. 2008b	Mastery climate 3	-0.05	143	Anxiety concentration disruption
Abrahamsen et al. 2008b	Performance climate 1	0.03	143	Anxiety somatic
Abrahamsen et al. 2008b	Performance climate 2	0.16	143	Anxiety worry
Abrahamsen et al. 2008b	Performance climate 3	0.12	143	Anxiety concentration disruption
Abraldes et al. 2016	Mastery Climate 1	0.44	163	Satisfaction fun
Abraldes et al. 2016	Mastery Climate 2	-0.06	163	Satisfaction boredom
Alferman et al. 2013 s1	Mastery Climate 1	0.08	56	Satisfaction
Alferman et al. 2013 s1	Performance Climate 1	-0.03	56	Satisfaction
Alferman et al. 2013 s2	Mastery Climate 1	0.35	117	Satisfaction
Alferman et al. 2013 s2	Performance Climate 1	0.17	117	Satisfaction
Al-Yaaribi & Kavussanu 2018	Mastery climate 1	0.4	358	Fun
Al-Yaaribi & Kavussanu 2018	Mastery climate 2	-0.23	358	Anger
Al-Yaaribi & Kavussanu 2018	Performance climate 1	-0.14	358	Fun

Al-Yaaribi & Kavussanu 2018	Performance climate 2	0.17	358	Anger
Amaro et al. 2023	Task-involving climate 1	0.6	109	Enjoyment
Atkins et al. 2013	Task Climate 2	0.13	227	Enjoyment
Atkins et al. 2013	Task Climate 3	0.23	227	Happy
Atkins et al. 2013	Task Climate 4	0.15	227	Fun
Atkins et al. 2013	Task Climate 6	0.21	227	Enjoyment
Atkins et al. 2013	Task Climate 7	0.22	227	Happy
Atkins et al. 2013	Task Climate 8	0.22	227	Fun
Atkins et al. 2015 s1	Task Climate 10	0.33	205	Enjoyment
Atkins et al. 2015 s1	Task Climate 11	0.28	205	Happy
Atkins et al. 2015 s1	Task Climate 12	0.28	205	Fun
Atkins et al. 2015 s1	Task Climate 16	0.29	205	Enjoyment
Atkins et al. 2015 s1	Task Climate 17	0.27	205	Happy
Atkins et al. 2015 s1	Task Climate 18	0.26	205	Fun
Atkins et al. 2015 s1	Task Climate 22	0.09	205	Enjoyment
Atkins et al. 2015 s1	Task Climate 23	0.16	205	Happy
Atkins et al. 2015 s1	Task Climate 24	0.11	205	Fun
Atkins et al. 2015 s1	Task Climate 28	0.09	205	Enjoyment
Atkins et al. 2015 s1	Task Climate 29	0.14	205	Happy
Atkins et al. 2015 s1	Task Climate 30	0.13	205	Fun
Atkins et al. 2015 s1	Task Climate 34	0.28	205	Enjoyment
Atkins et al. 2015 s1	Task Climate 35	0.3	205	Happy
Atkins et al. 2015 s1	Task Climate 36	0.3	205	Fun
Atkins et al. 2015 s1	Task Climate 4	0.3	205	Enjoyment
Atkins et al. 2015 s1	Task Climate 5	0.3	205	Happy
Atkins et al. 2015 s1	Task Climate 6	0.3	205	Fun
Atkins et al. 2015 s2	Task Climate 10	0.45	205	Enjoyment
Atkins et al. 2015 s2	Task Climate 11	0.44	205	Happy
Atkins et al. 2015 s2	Task Climate 12	0.4	205	Fun
Atkins et al. 2015 s2	Task Climate 16	0.38	200	Enjoyment
Atkins et al. 2015 s2	Task Climate 17	0.34	200	Happy

Atkins et al. 2015 s2	Task Climate 18	0.33	200	Fun
Atkins et al. 2015 s2	Task Climate 22	0.26	200	Enjoyment
Atkins et al. 2015 s2	Task Climate 23	0.24	200	Happy
Atkins et al. 2015 s2	Task Climate 24	0.24	200	Fun
Atkins et al. 2015 s2	Task Climate 28	0.33	200	Enjoyment
Atkins et al. 2015 s2	Task Climate 29	0.31	200	Happy
Atkins et al. 2015 s2	Task Climate 30	0.3	200	Fun
Atkins et al. 2015 s2	Task Climate 34	0.31	200	Enjoyment
Atkins et al. 2015 s2	Task Climate 35	0.34	200	Happy
Atkins et al. 2015 s2	Task Climate 36	0.33	200	Fun
Atkins et al. 2015 s2	Task Climate 4	0.28	205	Enjoyment
Atkins et al. 2015 s2	Task Climate 5	0.31	205	Happy
Atkins et al. 2015 s2	Task Climate 6	0.31	205	Fun
Balaguer et al. 1999	Ego Climate 1	-0.16	219	Satisfaction with results
Balaguer et al. 1999	Ego Climate 2	0.13	219	Satisfaction with level of play
Balaguer et al. 1999	Ego Climate 3	-0.41	219	Satisfaction with coach
Balaguer et al. 1999	Task Climate 1	0.23	219	Satisfaction with results
Balaguer et al. 1999	Task Climate 2	0.23	219	Satisfaction with level of play
Balaguer et al. 1999	Task Climate 3	0.41	219	Satisfaction with coach
Balaguer et al. 2002	Ego Climate 1	0.11	181	Satisfaction with level of play
Balaguer et al. 2002	Task Climate 1	0.21	181	Satisfaction with level of play
Baric 2011	Mastery climate 1	0.157	388	Pressure/tension
Baric 2011	Performance climate 1	-0.129	388	Pressure/tension
Bekiari & Syrmpas 2015	Mastery climate 1	0.835	324	Satisfaction
Bekiari & Syrmpas 2015	Performance climate 1	-0.884	324	Satisfaction
Blecharzet et al. 2014 s1	Task-involving 1	0.62	56	Satisfaction
Blecharzet et al. 2014 s2	Ego-involving 1	-0.08	113	Satisfaction
Blecharzet et al. 2014 s2	Ego-involving 2	-0.09	113	Satisfaction
Blecharzet et al. 2014 s2	Task-involving 1	0.25	113	Satisfaction
Blecharzet et al. 2014 s2	Task-involving 2	0.07	113	Satisfaction
Boixadós et al. 2004	Ego Climate 1	-0.09	472	Satisfaction

Boixadós et al. 2004	Ego Climate 2	0.05	472	Enjoyment
Boixadós et al. 2004	Task Climate 1	0.56	472	Satisfaction
Boixadós et al. 2004	Task Climate 2	0.13	472	Enjoyment
Bono & Livi 2016	Ego Climate 1	-0.123	96	Satisfaction with performance
Bono & Livi 2016	Ego Climate 2	0.174	96	Pressure/tension
Bono & Livi 2016	Task Climate 1	0.283	96	Satisfaction with performance
Bono & Livi 2016	Task Climate 2	-0.209	96	Pressure/tension
Bortoli et al. 2009	Mastery climate 1	0.235	473	Pleasant states
Bortoli et al. 2009	Mastery climate 2	-0.159	473	Unpleasant states
Bortoli et al. 2009	Performance climate 1	-0.095	473	Pleasant states
Bortoli et al. 2009	Performance climate 2	0.218	473	Unpleasant states
Bortoli et al. 2011	Ego Climate 1	-0.083	320	Pleasant psycholosocial states
Bortoli et al. 2011	Mastery climate 1	0.205	320	Pleasant psycholosocial states
Bortoli et al. 2012	Mastery climate 1	0.24	320	Pleasant states
Bortoli et al. 2012	Mastery climate 2	-0.24	320	Unpleasant states
Bortoli et al. 2012	Performance climate 1	-0.02	320	Pleasant states
Bortoli et al. 2012	Performance climate 2	0.2	382	Unpleasant states
Calvo & Topa 2019	Ego-oriented climate 1	-0.05	151	Satisfaction
Calvo & Topa 2019	Task-oriented climate 1	0.3	151	Satisfaction
Carr & Wyon 2003	Mastery climate 1	0.01	181	Anxiety somatic
Carr & Wyon 2003	Mastery climate 2	-0.12	181	Anxiety worry
Carr & Wyon 2003	Mastery climate 3	-0.25	181	Anxiety concentration disruption
Carr & Wyon 2003	Performance climate 1	0.1	181	Anxiety somatic
Carr & Wyon 2003	Performance climate 2	0.32	181	Anxiety worry
Carr & Wyon 2003	Performance climate 3	0.27	181	Anxiety concentration disruption
Cecchini et al. 2005	Performance climate 1	-0.371	82	Fun fair play
Cecchini et al. 2005	Performance climate 2	0.445	82	Anxiety error
Cecchini et al. 2005	Task Climate 1	0.467	82	Fun fair play
Cecchini et al. 2005	Task Climate 2	-0.118	82	Anxiety error
Cumming et al. 2007	Ego Climate 1	-0.23	268	Fun this season
Cumming et al. 2007	Task Climate 1	0.4	268	Fun this season

Curran et al. 2015	Mastery Climate 2	0.54	260	Well-being vigor
Curran et al. 2015	Mastery Climate 4	0.58	260	Well-being enthusiasm
Curran et al. 2015	Performance Climate 2	-0.09	260	Well-being vigor
Curran et al. 2015	Performance Climate 4	-0.16	260	Well-being enthusiasm
Dorsch et al. 2016	Mastery Climate 3	0.19	226	Positive affect
Dorsch et al. 2016	Mastery Climate 4	-0.16	226	Negative affect
Dorsch et al. 2016	Mastery Climate 7	0.28	226	Positive affect
Dorsch et al. 2016	Mastery Climate 8	0.09	226	Negative affect
Dorsch et al. 2016	Performance Climate 3	-0.19	226	Positive affect
Dorsch et al. 2016	Performance Climate 4	0.04	226	Negative affect
Dorsch et al. 2016	Performance Climate 7	0.01	226	Positive affect
Dorsch et al. 2016	Performance Climate 8	0.01	226	Negative affect
Draugelis et al. 2014	Ego Climate 1	0.04	182	Anxiety somatic
Draugelis et al. 2014	Ego Climate 2	0.17	182	Anxiety worry
Draugelis et al. 2014	Ego Climate 3	0.17	182	Anxiety concentration disruption
Draugelis et al. 2014	Ego Climate 5	-0.13	182	Well-being vigor
Draugelis et al. 2014	Ego Climate 7	-0.1	182	Well-being enthusiasm
Draugelis et al. 2014	Task Climate 1	-0.12	182	Anxiety somatic
Draugelis et al. 2014	Task Climate 2	-0.04	182	Anxiety worry
Draugelis et al. 2014	Task Climate 3	-0.07	182	Anxiety concentration disruption
Draugelis et al. 2014	Task Climate 5	0.34	182	Well-being vigor
Draugelis et al. 2014	Task Climate 7	0.25	182	Well-being enthusiasm
Eys et al. 2013	Ego-involving climate 1	-0.11	997	Enjoyment - self-referenced
Eys et al. 2013	Ego-involving climate 2	0.21	997	Enjoyment - other-referenced
Eys et al. 2013	Task-involving climate 1	0.35	997	Enjoyment - self-referenced
Eys et al. 2013	Task-involving climate 2	0.09	997	Enjoyment - other-referenced
García-Calvo et al. 2014	Ego Climate 1	-0.2	303	Satisfaction with participation
García-Calvo et al. 2014	Ego Climate 2	-0.06	303	Satisfaction with participation
García-Calvo et al. 2014	Ego Climate 3	-0.17	303	Satisfaction with participation
García-Calvo et al. 2014	Ego Climate 4	-0.06	303	Satisfaction with participation
García-Calvo et al. 2014	Task Climate 1	0.19	303	Satisfaction with participation

García-Calvo et al. 2014	Task Climate 2	0.05	303	Satisfaction with participation
García-Calvo et al. 2014	Task Climate 3	0.22	303	Satisfaction with participation
García-Calvo et al. 2014	Task Climate 4	0	303	Satisfaction with participation
Garcia-Mas et al. 2011	Ego Climate 1	0.178	54	Anxiety somatic
Garcia-Mas et al. 2011	Ego Climate 2	0.055	54	Anxiety concentration disruption
Garcia-Mas et al. 2011	Ego Climate 3	0.252	54	Anxiety worry
Garcia-Mas et al. 2011	Task Climate 1	-0.13	54	Anxiety worry
Garcia-Mas et al. 2011	Task Climate 2	-0.22	54	Anxiety somatic
Garcia-Mas et al. 2011	Task Climate 3	0.108	54	Anxiety concentration disruption
Gillham et al. 2013	Ego Climate 1	-0.13	396	Enjoyment
Gillham et al. 2013	Ego Climate 2	0.2	396	Boredom
Gillham et al. 2013	Mastery climate 1	0.36	396	Enjoyment
Gillham et al. 2013	Mastery climate 2	-0.16	396	Boredom
Gjesdal et al. 2018	Mastery Climate 1	0.58	1359	Enjoyment
Gjesdal et al. 2018	Mastery Climate 2	-0.21	1359	Anxiety
Gjesdal et al. 2018	Performance Climate 1	-0.43	1359	Enjoyment
Gjesdal et al. 2018	Performance Climate 2	0.38	1359	Anxiety
Gómez-López et al. 2020	Ego-involving 1	0.14	479	Anxiety somatic
Gómez-López et al. 2020	Ego-involving 2	-0.19	479	Anxiety cognitive
Gómez-López et al. 2020	Task-involving 1	-0.07	479	Anxiety somatic
Gómez-López et al. 2020	Task-involving 2	-0.01	479	Anxiety cognitive
Guzmán & García 2014	Mastery Climate 2	0.342	303	Satisfaction/interest
Guzmán & García 2014	Mastery Climate 3	0.149	303	Psychological well-being
Guzmán & García 2014	Performance Climate 2	-0.109	303	Satisfaction/interest
Guzmán & García 2014	Performance Climate 3	-0.202	303	Psychological well-being
Habeeb et al. 2023	Mastery Climate 1	0.23	150	Vigor
Habeeb et al. 2023	Mastery Climate 2	0.17	150	Enthusiasm
Habeeb et al. 2023	Mastery Climate 3	0.27	150	Vigor
Habeeb et al. 2023	Mastery Climate 4	0.34	150	Enthusiasm
Habeeb et al. 2023	Mastery Climate 5	0.36	150	Vigor
Habeeb et al. 2023	Mastery Climate 6	0.34	150	Enthusiasm

Habeeb et al. 2023	Performance Climate 1	-0.13	150	Vigor
Habeeb et al. 2023	Performance Climate 2	-0.11	150	Enthusiasm
Habeeb et al. 2023	Performance Climate 3	0.19	150	Vigor
Habeeb et al. 2023	Performance Climate 4	0.13	150	Enthusiasm
Habeeb et al. 2023	Performance Climate 5	0.12	150	Vigor
Habeeb et al. 2023	Performance Climate 6	-0.01	150	Enthusiasm
Harwood et al. 2019	Ego 1	0.12	92	Vigor
Harwood et al. 2019	Ego 2	-0.006	92	Vigor
Harwood et al. 2019	Ego 3	-0.06	92	Enthusiasm
Harwood et al. 2019	Ego 4	-0.16	92	Enthusiasm
Harwood et al. 2019	Task 1	0.26	92	Vigor
Harwood et al. 2019	Task 2	0.26	92	Vigor
Harwood et al. 2019	Task 3	0.22	92	Vigor
Harwood et al. 2019	Task 4	0.19	92	Enthusiasm
Harwood et al. 2019	Task 5	0.3	92	Enthusiasm
Harwood et al. 2019	Task 6	0.28	92	Enthusiasm
Haugen et al. 2020	Mastery climate 1	0.271	532	Satisfaction personal treatment
Haugen et al. 2020	Performance climate 1	-0.299	532	Satisfaction personal treatment
Holgado et al. 2010	Ego-involving 1	0.203	511	Satisfaction normative success
Holgado et al. 2010	Task-involving 1	0.185	511	Satisfaction normative success
Jaakkola et al. 2015	Ego-involving climate 1	0.17	265	Enjoyment
Jaakkola et al. 2015	Task-involving climate 1	0.54	265	Enjoyment
Kipp & Weiss 2013	Mastery Climate 6	0.23	309	Positive affect
Kipp & Weiss 2013	Performance Climate 6	-0.21	309	Positive affect
Lemyre et al. 2008	Mastery Climate 2	0.17	141	Satisfaction performance
Lemyre et al. 2008	Performance Climate 2	-0.07	141	Satisfaction performance
MacDonald et al. 2011	Ego 1	0.01	510	Self
MacDonald et al. 2011	Ego 2	0.32	510	Other
MacDonald et al. 2011	Ego 3	0.04	510	Effort
MacDonald et al. 2011	Ego 4	0.01	510	Competitive excitement
MacDonald et al. 2011	Ego 5	-0.03	510	Affiliation with peers

MacDonald et al. 2011	Ego 6	-0.1	510	Positive parent involvement
MacDonald et al. 2011	Task 1	0.31	510	Self
MacDonald et al. 2011	Task 2	0.09	510	Other
MacDonald et al. 2011	Task 3	0.29	510	Effort
MacDonald et al. 2011	Task 4	0.33	510	Competitive excitement
MacDonald et al. 2011	Task 5	0.33	510	Affiliation with peers
MacDonald et al. 2011	Task 6	0.34	510	Positive parent involvement
Monteiro et al. 2018	Ego-involving 2	-0.15	799	Enjoyment
Monteiro et al. 2018	Task-involving 2	0.159	799	Enjoyment
Mora et al. 2014 s1	Ego Climate 1	0.15	20	Anxiety worry
Mora et al. 2014 s1	Ego Climate 2	0.577	20	Anxiety somatic
Mora et al. 2014 s1	Ego Climate 3	0.347	20	Lack of concentration
Mora et al. 2014 s1	Ego Climate 4	0.07	20	Anxiety worry
Mora et al. 2014 s1	Ego Climate 5	0.055	20	Anxiety somatic
Mora et al. 2014 s1	Ego Climate 6	-0.018	20	Lack of concentration
Mora et al. 2014 s1	Task Climate 1	-0.034	20	Anxiety worry
Mora et al. 2014 s1	Task Climate 2	-0.31	20	Anxiety somatic
Mora et al. 2014 s1	Task Climate 3	-0.154	20	Lack of concentration
Mora et al. 2014 s1	Task Climate 4	0.107	20	Anxiety worry
Mora et al. 2014 s1	Task Climate 5	-0.249	20	Anxiety somatic
Mora et al. 2014 s1	Task Climate 6	-0.169	20	Lack of concentration
Mora et al. 2014 s2	Ego Climate 1	0.395	20	Anxiety worry
Mora et al. 2014 s2	Ego Climate 2	0.503	20	Anxiety somatic
Mora et al. 2014 s2	Ego Climate 3	0.254	20	Lack of concentration
Mora et al. 2014 s2	Ego Climate 4	0.259	20	Anxiety worry
Mora et al. 2014 s2	Ego Climate 5	0.055	20	Anxiety somatic
Mora et al. 2014 s2	Ego Climate 6	-0.255	20	Lack of concentration
Mora et al. 2014 s2	Task Climate 1	-0.262	20	Anxiety worry
Mora et al. 2014 s2	Task Climate 2	-0.331	20	Anxiety somatic
Mora et al. 2014 s2	Task Climate 3	-0.412	20	Lack of concentration
Mora et al. 2014 s2	Task Climate 4	-0.337	20	Anxiety worry

Mora et al. 2014 s2	Task Climate 5	-0.392	20	Anxiety somatic
Mora et al. 2014 s2	Task Climate 6	-0.335	20	Lack of concentration
Morales-Belando et al. 2021	Ego Climate 1	-0.21	94	Enjoyment
Morales-Belando et al. 2021	Task Climate 1	0.08	94	Enjoyment
Newton et al. 2000	Ego Climate 1	-0.31	385	Enjoyment/interest
Newton et al. 2000	Ego Climate 2	0.4	385	Pressure/tension
Newton et al. 2000	Ego Climate 3	-0.2	385	Satisfaction team
Newton et al. 2000	Task Climate 1	0.52	385	Enjoyment/interest
Newton et al. 2000	Task Climate 2	-0.24	385	Pressure/tension
Newton et al. 2000	Task Climate 3	0.41	385	Satisfaction team
Nordin-Bates et al. 2012	Ego Climate 1	0.13	327	Anxiety total score
Nordin-Bates et al. 2012	Ego Climate 2	0.31	327	Anxiety total score
Nordin-Bates et al. 2012	Task Climate 1	0.01	327	Anxiety total score
Nordin-Bates et al. 2012	Task Climate 2	-0.12	327	Anxiety total score
Ntoumanis & Biddle 1998	Ego Climate 1	-0.03	146	Anxiety cognitive intensity
Ntoumanis & Biddle 1998	Ego Climate 2	-0.05	146	Anxiety somatic intensity
Ntoumanis & Biddle 1998	Mastery Climate 1	-0.09	146	Anxiety cognitive intensity
Ntoumanis & Biddle 1998	Mastery Climate 2	-0.02	146	Anxiety somatic intensity
Núñez et al. 2011	Cooperative learning 4	0.3	399	Positive emotions
Núñez et al. 2011	Effort improvement 4	0.28	399	Positive emotions
Núñez et al. 2011	Important role 4	0.23	399	Positive emotions
O'Rourke et al. 2011	Mastery climate 1	-0.24	307	Anxiety
O'Rourke et al. 2011	Mastery climate 2	-0.18	307	Anxiety
O'Rourke et al. 2011	Mastery climate 3	-0.24	307	Anxiety
O'Rourke et al. 2011	Performance climate 1	0.34	307	Anxiety
O'Rourke et al. 2011	Performance climate 2	0.3	307	Anxiety
O'Rourke et al. 2011	Performance climate 3	0.24	307	Anxiety
O'Rourke et al. 2014	Ego climate 1	0.24	228	Anxiety
O'Rourke et al. 2014	Ego climate 2	0.19	228	Anxiety
O'Rourke et al. 2014	Mastery climate 1	-0.24	228	Anxiety
O'Rourke et al. 2014	Mastery climate 2	-0.16	228	Anxiety

Papaioannou et al. 2008	Mastery climate 1	0.33	863	Satisfaction
Papaioannou et al. 2008	Mastery climate 2	0.31	863	Satisfaction
Papaioannou et al. 2008	Mastery climate 3	0.29	863	Satisfaction
Pensgaard & Roberts 2000	Ego Climate 1	0.0289	69	Distress total score
Pensgaard & Roberts 2000	Mastery climate 1	0	69	Distress total score
Pineda-Espejel et al. 2015	Ego climate 1	0.14	211	Anxiety cognitive
Pineda-Espejel et al. 2015	Ego climate 2	0.13	211	Anxiety somatic
Pineda-Espejel et al. 2015	Task climate 1	-0.07	211	Anxiety cognitive
Pineda-Espejel et al. 2015	Task climate 2	-0.08	211	Anxiety somatic
Pineda-Espejel et al. 2021	Task climate 1	-0.08	217	Anxiety cognitive
Pineda-Espejel et al. 2021	Task climate 2	-0.04	217	Anxiety somatic
Quested & Duda 2009	Ego climate 5	-0.33	59	Positive affect
Quested & Duda 2009	Ego climate 6	0.37	59	Negative affect
Quested & Duda 2009	Task climate 5	0.48	59	Positive affect
Quested & Duda 2009	Task climate 6	-0.47	59	Negative affect
Quested & Duda 2010	Ego-involving 4	-0.25	392	Positive affect
Quested & Duda 2010	Ego-involving 5	0.33	392	Negative affect
Quested & Duda 2010	Task-involving 4	0.41	392	Positive affect
Quested & Duda 2010	Task-involving 5	-0.29	392	Negative affect
Robazza et al. 2021	Ego Climate 1	-0.383	302	Pleasant emotions
Robazza et al. 2021	Ego Climate 2	0.176	302	Anxiety
Robazza et al. 2021	Mastery Climate 1	0.409	302	Pleasant emotions
Robazza et al. 2021	Mastery Climate 2	-0.228	302	Anxiety
Robazza et al. 2022	Ego Climate 1	0.06	459	Anxiety
Robazza et al. 2022	Ego Climate 2	0.29	459	Dejection
Robazza et al. 2022	Ego Climate 3	0.33	459	Anger
Robazza et al. 2022	Ego Climate 4	-0.02	459	Excitement
Robazza et al. 2022	Ego Climate 5	-0.08	459	Happiness
Robazza et al. 2022	Ego Climate 6	-0.27	459	Emotions
Robazza et al. 2022	Ego Climate 7	-0.06	459	Anxiety
Robazza et al. 2022	Mastery Climate 1	-0.01	459	Anxiety

Robazza et al. 2022	Mastery Climate 2	-0.15	459	Dejection
Robazza et al. 2022	Mastery Climate 3	-0.13	459	Anger
Robazza et al. 2022	Mastery Climate 4	0.22	459	Excitement
Robazza et al. 2022	Mastery Climate 5	0.25	459	Happiness
Robazza et al. 2022	Mastery Climate 6	0.33	459	Emotions
Robazza et al. 2022	Mastery Climate 7	0.15	459	Anxiety
Ruiz et al. 2017	Ego-involving climate 1	-0.09	494	Pleasant states intensity
Ruiz et al. 2017	Ego-involving climate 10	-0.09	494	Pleasant states impact
Ruiz et al. 2017	Ego-involving climate 11	0	494	Anxiety impact
Ruiz et al. 2017	Ego-involving climate 12	0.08	494	Anger impact
Ruiz et al. 2017	Ego-involving climate 2	0.13	494	Anxiety intensity
Ruiz et al. 2017	Ego-involving climate 3	0.11	494	Anger intensity
Ruiz et al. 2017	Ego-involving climate 4	0.01	494	Pleasant states intensity
Ruiz et al. 2017	Ego-involving climate 5	0.2	494	Anxiety intensity
Ruiz et al. 2017	Ego-involving climate 6	0.25	494	Anger intensity
Ruiz et al. 2017	Ego-involving climate 7	-0.07	494	Pleasant states impact
Ruiz et al. 2017	Ego-involving climate 8	-0.09	494	Anxiety impact
Ruiz et al. 2017	Ego-involving climate 9	0	494	Anger impact
Ruiz et al. 2017	Task-involving climate 1	0.06	494	Pleasant states intensity
Ruiz et al. 2017	Task-involving climate 10	0.08	494	Pleasant states impact
Ruiz et al. 2017	Task-involving climate 11	0	494	Anxiety impact
Ruiz et al. 2017	Task-involving climate 12	-0.13	494	Anger impact
Ruiz et al. 2017	Task-involving climate 2	-0.15	494	Anxiety intensity
Ruiz et al. 2017	Task-involving climate 3	0.07	494	Anger intensity
Ruiz et al. 2017	Task-involving climate 4	0.03	494	Pleasant states intensity
Ruiz et al. 2017	Task-involving climate 5	-0.19	494	Anxiety intensity
Ruiz et al. 2017	Task-involving climate 6	-0.24	494	Anger intensity
Ruiz et al. 2017	Task-involving climate 7	0.11	494	Pleasant states impact
Ruiz et al. 2017	Task-involving climate 8	0.07	494	Anxiety impact
Ruiz et al. 2017	Task-involving climate 9	0.18	494	Anger impact
Ruiz et al. 2019	Ego climate 1	-0.08	217	Pleasant states intensity

Ruiz et al. 2019	Ego climate 10	0.2	217	Anxiety intensity dysfunctional
Ruiz et al. 2019	Ego climate 11	0.17	217	Anger intensity functional
Ruiz et al. 2019	Ego climate 12	0.32	217	Anger intensity dysfunctional
Ruiz et al. 2019	Ego climate 13	-0.16	217	Pleasant states impact
Ruiz et al. 2019	Ego climate 14	0.12	217	Anxiety intensity dysfunctional
Ruiz et al. 2019	Ego climate 15	-0.05	217	Anger impact functional
Ruiz et al. 2019	Ego climate 16	0.14	217	Anger impact dysfunctional
Ruiz et al. 2019	Ego climate 2	0.19	217	Anxiety intensity dysfunctional
Ruiz et al. 2019	Ego climate 3	0.12	217	Anger intensity functional
Ruiz et al. 2019	Ego climate 4	0.21	217	Anger intensity dysfunctional
Ruiz et al. 2019	Ego climate 5	-0.08	217	Pleasant states impact
Ruiz et al. 2019	Ego climate 6	-0.08	217	Anxiety intensity dysfunctional
Ruiz et al. 2019	Ego climate 7	-0.07	217	Anger impact functional
Ruiz et al. 2019	Ego climate 8	0.14	217	Anger impact dysfunctional
Ruiz et al. 2019	Ego climate 9	-0.08	217	Pleasant states intensity
Ruiz et al. 2019	Task climate 1	0.08	217	Pleasant states intensity
Ruiz et al. 2019	Task climate 10	-0.19	217	Anxiety intensity dysfunctional
Ruiz et al. 2019	Task climate 11	-0.1	217	Anger intensity functional
Ruiz et al. 2019	Task climate 12	-0.19	217	Anger intensity dysfunctional
Ruiz et al. 2019	Task climate 13	0.15	217	Pleasant states impact
Ruiz et al. 2019	Task climate 14	-0.09	217	Anxiety intensity dysfunctional
Ruiz et al. 2019	Task climate 15	0.12	217	Anger impact functional
Ruiz et al. 2019	Task climate 16	-0.18	217	Anger impact dysfunctional
Ruiz et al. 2019	Task climate 2	-0.21	217	Anxiety intensity dysfunctional
Ruiz et al. 2019	Task climate 3	0.07	217	Anger intensity functional
Ruiz et al. 2019	Task climate 4	-0.22	217	Anger intensity dysfunctional
Ruiz et al. 2019	Task climate 5	0.11	217	Pleasant states impact
Ruiz et al. 2019	Task climate 6	-0.06	217	Anxiety intensity dysfunctional
Ruiz et al. 2019	Task climate 7	0.28	217	Anger impact functional
Ruiz et al. 2019	Task climate 8	-0.14	217	Anger impact dysfunctional
Ruiz et al. 2019	Task climate 9	0.1	217	Pleasant states intensity

Santos-Rosa et al. 2022	Ego-involving 1	-0.06	258	Positivity
Santos-Rosa et al. 2022	Ego-involving 2	0.24	258	Anxiety somatic
Santos-Rosa et al. 2022	Ego-involving 3	0.17	258	Anxiety worry
Santos-Rosa et al. 2022	Task-involving 1	0.26	258	Positivity
Santos-Rosa et al. 2022	Task-involving 2	-0.04	258	Anxiety somatic
Santos-Rosa et al. 2022	Task-involving 3	-0.08	258	Anxiety worry
Sari & Bizan 2022	Ego-involving Climate 2	-0.1	180	Enthusiasm
Sari & Bizan 2022	Ego-involving Climate 1	-0.07	180	Vigor
Sari & Bizan 2022	Task-involving Climate 1	0.36	180	Vigor
Sari & Bizan 2022	Task-involving Climate 2	0.42	180	Enthusiasm
Scott et al. 2021	Ego-involving 1	-0.12	109	Hope
Scott et al. 2021	Ego-involving 2	-0.14	109	Happiness
Scott et al. 2021	Task-involving 1	0.26	109	Hope
Scott et al. 2021	Task-involving 2	0.18	109	Happiness
Sheehan et al. 2018a	Ego Climate w1 1	0.407	38	Total Mood Score
Sheehan et al. 2018a	Ego Climate w1 2	0.4	38	Depression symptoms
Sheehan et al. 2018a	Ego Climate w1 4	0.452	38	Anxiety Trait
Sheehan et al. 2018a	Ego Climate w2 1	0.404	38	Total Mood Score
Sheehan et al. 2018a	Ego Climate w2 2	0.218	38	Depression symptoms
Sheehan et al. 2018a	Ego Climate w2 4	0.52	38	Anxiety Trait
Sheehan et al. 2018a	Task Climate w1 1	-0.018	38	Total Mood Score
Sheehan et al. 2018a	Task Climate w1 2	-0.261	38	Depression symptoms
Sheehan et al. 2018a	Task Climate w1 4	-0.48	38	Anxiety Trait
Sheehan et al. 2018a	Task Climate w2 1	-0.242	38	Total Mood Score
Sheehan et al. 2018a	Task Climate w2 2	-0.153	38	Depression symptoms
Sheehan et al. 2018a	Task Climate w2 4	-0.488	38	Anxiety Trait
Sheehan et al. 2018b	Ego Climate 4	0.086	215	Total Mood Score
Sheehan et al. 2018b	Ego Climate 5	0.174	215	Depression symptoms
Sheehan et al. 2018b	Ego Climate 7	0.183	215	Anxiety Trait
Sheehan et al. 2018b	Task Climate 4	-0.131	215	Total Mood Score
Sheehan et al. 2018b	Task Climate 5	-0.103	215	Depression symptoms

Sheehan et al. 2018b	Task Climate 7	-0.242	215	Anxiety Trait
Smith, Balaguer et al. 2006	Ego-involving 1	-0.08	223	Enjoyment soccer
Smith, Balaguer et al. 2006	Ego-involving 2	0	223	Satisfaction performance
Smith, Balaguer et al. 2006	Ego-involving 3	-0.13	223	Satisfaction team
Smith, Balaguer et al. 2006	Task-involving 1	0.36	223	Enjoyment soccer
Smith, Balaguer et al. 2006	Task-involving 2	0.23	223	Satisfaction performance
Smith, Balaguer et al. 2006	Task-involving 3	0.24	223	Satisfaction team
Smith, Smoll et al. 2006	Ego climate 1	0.3	572	Anxiety somatic
Smith, Smoll et al. 2006	Ego climate 2	0.28	572	Anxiety worry
Smith, Smoll et al. 2006	Ego climate 3	0.35	572	Anxiety concentration disruption
Smith, Smoll et al. 2006	Mastery climate 1	-0.28	572	Anxiety somatic
Smith, Smoll et al. 2006	Mastery climate 2	-0.29	572	Anxiety worry
Smith, Smoll et al. 2006	Mastery climate 3	-0.32	572	Anxiety concentration disruption
Solstad & Lemyre 2014	Mastery Climate 1	0.405	202	Well-being subjective
Solstad & Lemyre 2014	Performance Climate 1	-0.18	202	Well-being subjective
Stark & Newton 2014	Ego Climate 1	-0.119	83	Positive affect
Stark & Newton 2014	Ego Climate 2	0.456	83	Negative affect
Stark & Newton 2014	Task Climate 1	0.487	83	Positive affect
Stark & Newton 2014	Task Climate 2	-0.249	83	Negative affect
Steffen et al. 2009	Mastery climate 1	0.07	1430	Anxiety somatic
Steffen et al. 2009	Mastery climate 2	0.02	1430	Anxiety worry
Steffen et al. 2009	Mastery climate 3	-0.05	1430	Anxiety concentration disruption
Steffen et al. 2009	Performance Climate 1	0.21	1430	Anxiety somatic
Steffen et al. 2009	Performance Climate 2	0.29	1430	Anxiety worry
Steffen et al. 2009	Performance Climate 3	0.29	1430	Anxiety concentration disruption
Tamminen et al. 2016	Ego Climate 1	-0.04	451	Enjoyment
Tamminen et al. 2016	Task Climate 1	0.5	451	Enjoyment
Trbojevic Jocic & Petrovic 2021	Task climate 2	0.205	383	Enjoyment
Trbojevic Jocic et al. 2020	Punishment for Mistakes 1	-0.14	117	Satisfaction
Trbojevic Jocic et al. 2020	Task climate 1	0.61	117	Satisfaction
Trbojevic Jocic et al. 2020	Unequal Recognition 1	-0.38	117	Satisfaction

Trenz & Zusho 2011	Mastery climate 1	0.6	119	Satisfaction swim
Trenz & Zusho 2011	Performance climate 1	-0.38	119	Satisfaction swim
Vazou et al. 2006	Mastery climate 2	0.39	493	Enjoyment
Vazou et al. 2006	Mastery climate 3	-0.03	493	Anxiety trait
Vazou et al. 2006	Mastery climate 5	0.31	493	Enjoyment
Vazou et al. 2006	Mastery climate 6	-0.06	493	Anxiety trait
Vazou et al. 2006	Performance climate 2	-0.16	493	Enjoyment
Vazou et al. 2006	Performance climate 3	0.15	493	Anxiety trait
Vazou et al. 2006	Performance climate 5	-0.14	493	Enjoyment
Vazou et al. 2006	Performance climate 6	0.21	493	Anxiety trait
Vosloo et al. 2009	Mastery climate 1	0.269	151	Anxiety cognitive facilitative
Vosloo et al. 2009	Mastery climate 2	0.275	151	Anxiety somatic facilitative
Walling et al. 1993	Mastery Climate 1	-0.18	169	Worry performance
Walling et al. 1993	Mastery Climate 2	0.39	169	Satisfaction team
Walling et al. 1993	Performance Climate 1	0.39	169	Worry performance
Walling et al. 1993	Performance Climate 2	-0.26	169	Satisfaction team
Weiss 2015	Mastery Climate 1	0.38	491	Enjoyment
Weiss 2015	Performance Climate 1	-0.19	491	Enjoyment
Weiss et al. 2009	Mastery Climate 1	0.22	141	Enjoyment
Weiss et al. 2009	Performance Climate 1	-0.2	141	Enjoyment
Wu et al. 2021	Ego climate 1	0.35	685	Depression
Wu et al. 2021	Ego climate 2	0.25	685	Anxiety
Wu et al. 2021	Task climate 1	-0.43	685	Depression
Wu et al. 2021	Task climate 2	-0.32	685	Anxiety
Zanatta et al. 2018	Ego-involving climate 1	-0.03	824	Enjoyment
Zanatta et al. 2018	Task-involving climate 1	0.39	824	Enjoyment

Supplement Figures. Remove-one study figures.

Meta Analysis

Study name	Subgroup within study	Statistics with study removed				
		Point	Lower limit	Upper limit	Z-Value	p-Value
Al-Yaaribi & Kavussanu 2018	Mastery climate 1	0.312	0.273	0.351	14.526	0.000
Amaro et al. 2023	Task-involving climate 1	0.309	0.269	0.347	14.541	0.000
Atkins et al. 2013	Combined	0.318	0.278	0.357	14.759	0.000
Atkins et al. 2015 s1	Combined	0.317	0.275	0.357	14.144	0.000
Atkins et al. 2015 s2	Combined	0.314	0.273	0.355	14.014	0.000
Boixadós et al. 2004	Task Climate 2	0.319	0.279	0.357	14.870	0.000
Bortoli et al. 2009	Mastery climate 1	0.316	0.276	0.355	14.640	0.000
Bortoli et al. 2011	Mastery climate 1	0.317	0.277	0.356	14.715	0.000
Bortoli et al. 2012	Mastery climate 1	0.316	0.276	0.355	14.662	0.000
Cecchini et al. 2005	Task Climate 1	0.312	0.272	0.351	14.581	0.000
Cumming et al. 2007	Task Climate 1	0.313	0.273	0.351	14.540	0.000
Curran et al. 2015	Combined	0.308	0.269	0.345	14.832	0.000
Dorsch et al. 2016	Combined	0.316	0.276	0.355	14.643	0.000
Draugelis et al. 2014	Combined	0.315	0.275	0.354	14.585	0.000
Eys et al. 2013	Combined	0.317	0.276	0.356	14.429	0.000
Gjesdal et al. 2018	Mastery Climate 1	0.305	0.271	0.339	16.308	0.000
Guzmán & García 2014	Mastery Climate 3	0.318	0.278	0.357	14.816	0.000
Habeeb et al. 2023	Combined	0.315	0.275	0.354	14.480	0.000
Harwood et al. 2019	Combined	0.316	0.276	0.355	14.600	0.000
Jaakkola et al. 2015	Task-involving climate 1	0.309	0.270	0.347	14.580	0.000
Kipp & Weiss 2013	Mastery Climate 6	0.316	0.276	0.355	14.679	0.000
Lemyre et al. 2008	Mastery Climate 2	0.317	0.277	0.356	14.786	0.000
MacDonald et al. 2011	Combined	0.316	0.274	0.356	14.072	0.000
Monteiro et al. 2018	Task-involving 2	0.318	0.278	0.357	14.792	0.000
Morales-Belando et al. 2021	Task Climate 1	0.318	0.279	0.356	14.885	0.000
Newton et al. 2000	Task Climate 1	0.309	0.270	0.347	14.617	0.000
Núñez et al. 2011	Combined	0.316	0.275	0.355	14.440	0.000
Quested & Duda 2009	Task climate 5	0.312	0.272	0.351	14.603	0.000
Quested & Duda 2010	Task-involving 4	0.312	0.272	0.351	14.523	0.000
Robazza et al. 2021	Mastery Climate 1	0.312	0.272	0.351	14.532	0.000
Robazza et al. 2022	Combined	0.316	0.275	0.355	14.410	0.000
Ruiz et al. 2017	Combined	0.320	0.282	0.357	15.706	0.000
Ruiz et al. 2019	Combined	0.319	0.281	0.357	15.247	0.000
Santos-Rosa et al. 2022	Task-involving 1	0.316	0.276	0.354	14.651	0.000
Sari & Bizan 2022	Combined	0.313	0.273	0.352	14.527	0.000
Scott et al. 2021	Combined	0.316	0.277	0.355	14.711	0.000
Smith, Balaguer et al. 2006	Combined	0.315	0.275	0.354	14.537	0.000
Solstad & Lemyre 2014	Mastery Climate 1	0.313	0.273	0.351	14.552	0.000
Stark & Newton 2014	Task Climate 1	0.312	0.272	0.350	14.572	0.000
Tamminen et al. 2016	Task Climate 1	0.310	0.270	0.348	14.611	0.000
Trbojevic Jocic & Petrovic 2021	Task climate 2	0.317	0.277	0.356	14.706	0.000
Vazou et al. 2006	Combined	0.314	0.273	0.353	14.434	0.000
Vosloo et al. 2009	Combined	0.315	0.275	0.354	14.627	0.000
Weiss 2015	Mastery Climate 1	0.313	0.273	0.352	14.510	0.000
Weiss et al. 2009	Mastery Climate 1	0.316	0.276	0.355	14.728	0.000
Zanatta et al. 2018	Task-involving climate 1	0.313	0.273	0.352	14.483	0.000
Pooled		0.314	0.275	0.353	14.810	0.000
Prediction Interval		0.314	0.043	0.543	0.000	0.000

Correlation (95% CI) with study removed

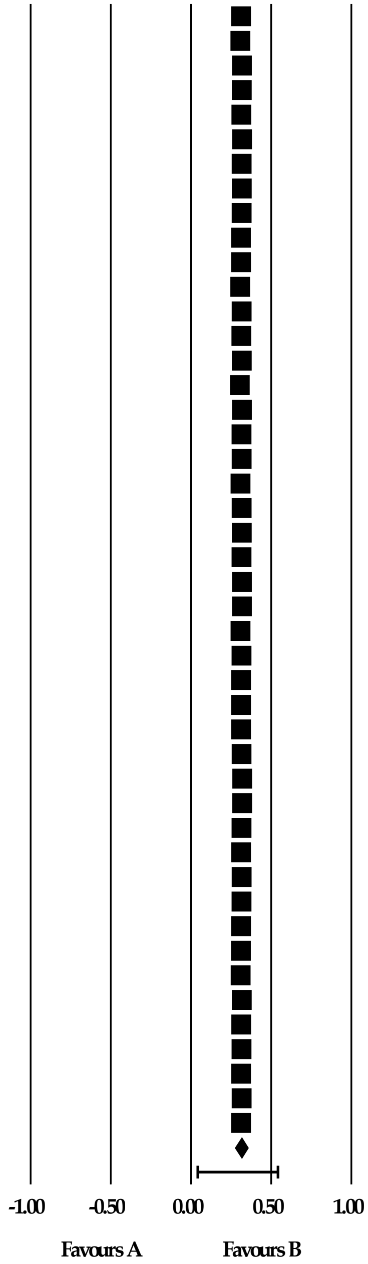

Supp Figure 1. Remove-one study results for task/mastery climate and positive affect/mood.

Study name	Subgroup within study	Statistics with study removed				
		Point	Lower limit	Upper limit	Z-Value	p-Value
Abrahamsen & Kristiansen 2015	Mastery climate 1	-0.134	-0.176	-0.093	-6.286	0.000
Abrahamsen et al. 2008a	Combined	-0.129	-0.172	-0.086	-5.812	0.000
Abrahamsen et al. 2008b	Combined	-0.129	-0.171	-0.085	-5.801	0.000
Al-Yaaribi & Kavussanu 2018	Mastery climate 2	-0.124	-0.167	-0.082	-5.650	0.000
Baric 2011	Mastery climate 1	-0.135	-0.176	-0.093	-6.287	0.000
Bono & Livi 2016	Task Climate 2	-0.126	-0.168	-0.083	-5.731	0.000
Bortoli et al. 2009	Mastery climate 2	-0.126	-0.169	-0.083	-5.699	0.000
Bortoli et al. 2012	Mastery climate 2	-0.124	-0.167	-0.081	-5.646	0.000
Carr & Wyon 2003	Combined	-0.127	-0.170	-0.084	-5.734	0.000
Cecchini et al. 2005	Task Climate 2	-0.127	-0.170	-0.085	-5.809	0.000
Dorsch et al. 2016	Combined	-0.130	-0.172	-0.087	-5.869	0.000
Draugelis et al. 2014	Combined	-0.129	-0.171	-0.085	-5.796	0.000
Garcia-Mas et al. 2011	Combined	-0.128	-0.171	-0.085	-5.828	0.000
Gjesdal et al. 2018	Mastery Climate 2	-0.125	-0.168	-0.081	-5.609	0.000
Gómez-López et al. 2020	Combined	-0.130	-0.173	-0.086	-5.832	0.000
Mora et al. 2014 s1	Combined	-0.127	-0.169	-0.084	-5.786	0.000
Mora et al. 2014 s2	Combined	-0.123	-0.165	-0.080	-5.628	0.000
Newton et al. 2000	Task Climate 2	-0.124	-0.167	-0.081	-5.640	0.000
Nordin-Bates et al. 2012	Combined	-0.129	-0.172	-0.086	-5.822	0.000
Ntoumanis & Biddle 1998	Combined	-0.129	-0.172	-0.086	-5.848	0.000
O'Rourke et al. 2011	Combined	-0.124	-0.167	-0.081	-5.625	0.000
O'Rourke et al. 2014	Combined	-0.125	-0.168	-0.082	-5.663	0.000
Pensgaard & Roberts 2000	Mastery climate 1	-0.129	-0.171	-0.087	-5.905	0.000
Pineda-Espejel et al. 2015	Combined	-0.129	-0.171	-0.086	-5.809	0.000
Pineda-Espejel et al. 2021	Combined	-0.129	-0.172	-0.086	-5.831	0.000
Quested & Duda 2009	Task climate 6	-0.122	-0.164	-0.080	-5.613	0.000
Quested & Duda 2010	Task-involving 5	-0.123	-0.165	-0.080	-5.613	0.000
Robazza et al. 2021	Mastery Climate 2	-0.137	-0.177	-0.095	-6.425	0.000
Robazza et al. 2022	Combined	-0.128	-0.171	-0.083	-5.622	0.000
Ruiz et al. 2017	Combined	-0.130	-0.174	-0.085	-5.643	0.000
Ruiz et al. 2019	Combined	-0.126	-0.170	-0.082	-5.587	0.000
Santos-Rosa et al. 2022	Combined	-0.129	-0.172	-0.086	-5.824	0.000
Sheehan et al. 2018a	Combined	-0.123	-0.166	-0.081	-5.631	0.000
Sheehan et al. 2018b	Combined	-0.126	-0.169	-0.083	-5.681	0.000
Smith, Smoll et al. 2006	Combined	-0.122	-0.163	-0.081	-5.779	0.000
Stark & Newton 2014	Task Climate 2	-0.125	-0.167	-0.082	-5.711	0.000
Steffen et al. 2009	Combined	-0.132	-0.173	-0.090	-6.153	0.000
Vazou et al. 2006	Combined	-0.130	-0.173	-0.086	-5.820	0.000
Walling et al. 1993	Mastery Climate 1	-0.126	-0.168	-0.083	-5.727	0.000
Wu et al. 2021	Combined	-0.119	-0.157	-0.081	-6.026	0.000
Pooled		-0.127	-0.169	-0.085	-5.866	0.000
Prediction Interval		-0.127	-0.365	0.127	0.000	0.000

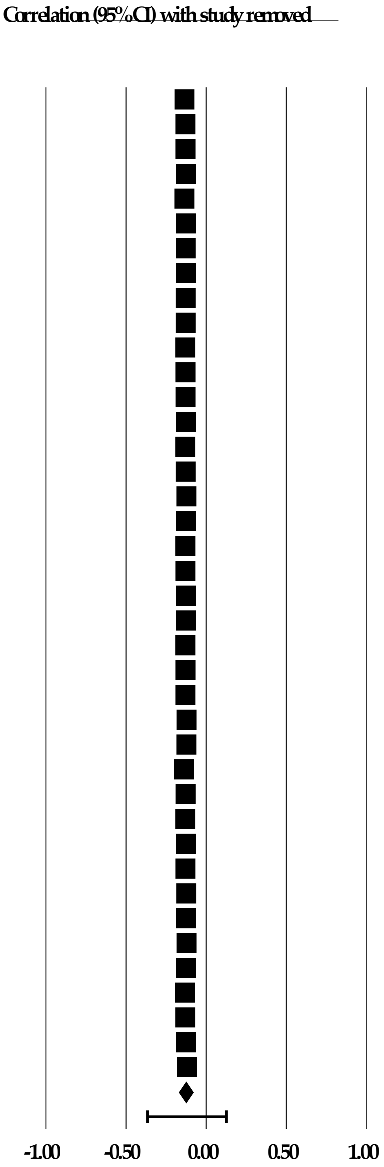

Supp Figure 2. Remove-one study results for task/mastery climate and negative affect/mood.

Study name	Subgroup within study	Statistics with study removed				
		Point	Lower limit	Upper limit	Z-Value	p-Value
Abraldes et al. 2016	Combined	0.381	0.288	0.466	7.563	0.000
Alferman et al. 2013 s1	Mastery Climate 1	0.383	0.293	0.466	7.768	0.000
Alferman et al. 2013 s2	Mastery Climate 1	0.373	0.282	0.458	7.472	0.000
Balaguer et al. 1999	Combined	0.377	0.282	0.464	7.283	0.000
Balaguer et al. 2002	Task Climate 1	0.380	0.288	0.465	7.599	0.000
Bekiari & Syrmpas 2015	Mastery climate 1	0.330	0.269	0.390	9.887	0.000
Blecharz et al. 2014 s1	Task-involving 1	0.361	0.270	0.445	7.317	0.000
Blecharz et al. 2014 s2	Combined	0.382	0.291	0.467	7.662	0.000
Boixadós et al. 2004	Task Climate 1	0.361	0.271	0.445	7.404	0.000
Bono & Livi 2016	Task Climate 1	0.376	0.285	0.461	7.552	0.000
Calvo & Topa 2019	Task-oriented climate 1	0.376	0.284	0.461	7.503	0.000
García-Calvo et al. 2014	Combined	0.385	0.295	0.468	7.823	0.000
Gillham et al. 2013	Combined	0.378	0.283	0.466	7.278	0.000
Guzmán & García 2014	Mastery Climate 2	0.374	0.281	0.460	7.382	0.000
Haugen et al. 2020	Mastery climate 1	0.378	0.284	0.464	7.366	0.000
Holgado et al. 2010	Task-involving 1	0.382	0.289	0.467	7.549	0.000
Newton et al. 2000	Task Climate 3	0.370	0.277	0.457	7.298	0.000
Papaioannou et al. 2008	Combined	0.376	0.272	0.472	6.638	0.000
Tirbojevic Jocic et al. 2020	Task climate 1	0.359	0.268	0.444	7.300	0.000
Trenz & Zusho 2011	Mastery climate 1	0.360	0.269	0.445	7.303	0.000
Walling et al. 1993	Mastery Climate 2	0.371	0.279	0.457	7.405	0.000
Pooled		0.372	0.283	0.455	7.683	0.000
Prediction Interval		0.372	-0.085	0.700	0.000	0.000

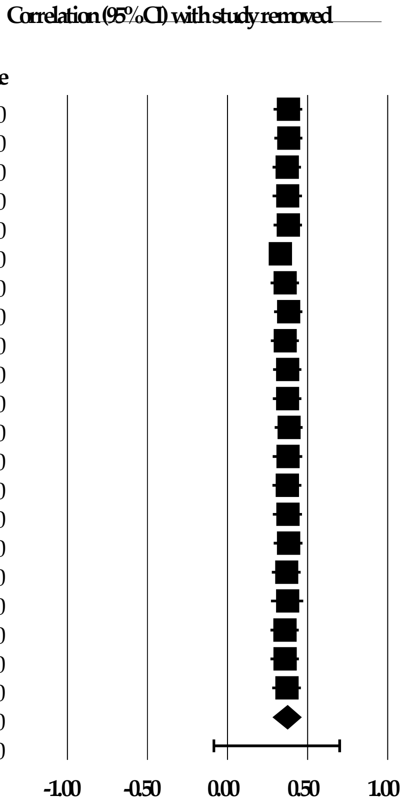

Supp Figure 3. Remove-one study results for task/mastery climate and satisfaction.

Study name	Subgroup within study	Statistics with study removed				
		Point	Lower limit	Upper limit	Z-Value	p-Value
Al-Yaaribi & Kavussanu 2018	Performance climate 1	-0.115	-0.163	-0.066	-4.600	0.000
Atkins et al. 2013	Combined	-0.124	-0.170	-0.078	-5.265	0.000
Boixadós et al. 2004	Ego Climate 2	-0.120	-0.168	-0.071	-4.827	0.000
Bortoli et al. 2009	Performance climate 1	-0.116	-0.164	-0.067	-4.634	0.000
Bortoli et al. 2011	Ego Climate 1	-0.116	-0.164	-0.068	-4.661	0.000
Bortoli et al. 2012	Performance climate 1	-0.118	-0.166	-0.069	-4.732	0.000
Cecchini et al. 2005	Performance climate 1	-0.110	-0.158	-0.062	-4.488	0.000
Cumming et al. 2007	Ego Climate 1	-0.112	-0.160	-0.064	-4.535	0.000
Curran et al. 2015	Combined	-0.115	-0.163	-0.066	-4.600	0.000
Dorsch et al. 2016	Combined	-0.116	-0.164	-0.067	-4.640	0.000
Draugelis et al. 2014	Combined	-0.115	-0.164	-0.067	-4.624	0.000
Eys et al. 2013	Combined	-0.120	-0.168	-0.072	-4.838	0.000
Gjesdal et al. 2018	Performance Climate-1	-0.102	-0.140	-0.063	-5.126	0.000
Guzmán & García 2014	Performance Climate-3	-0.113	-0.161	-0.065	-4.553	0.000
Habeeb et al. 2023	Combined	-0.120	-0.168	-0.071	-4.791	0.000
Harwood et al. 2019	Combined	-0.118	-0.166	-0.069	-4.721	0.000
Jaakkola et al. 2015	Ego-involving climate-1	-0.122	-0.170	-0.075	-5.000	0.000
Kipp & Weiss 2013	Performance Climate-6	-0.113	-0.161	-0.064	-4.546	0.000
Lemyre et al. 2008	Performance Climate-2	-0.116	-0.164	-0.068	-4.691	0.000
MacDonald et al. 2011	Combined	-0.120	-0.168	-0.071	-4.822	0.000
Monteiro et al. 2018	Ego-involving 2	-0.114	-0.163	-0.065	-4.557	0.000
Morales-Belando et al. 2021	Ego Climate 1	-0.113	-0.161	-0.065	-4.590	0.000
Newton et al. 2000	Ego Climate 1	-0.110	-0.157	-0.062	-4.485	0.000
Quested & Duda 2009	Ego climate 5	-0.112	-0.159	-0.064	-4.540	0.000
Quested & Duda 2010	Ego-involving 4	-0.112	-0.159	-0.063	-4.515	0.000
Robazza et al. 2021	Ego Climate 1	-0.108	-0.154	-0.060	-4.449	0.000
Robazza et al. 2022	Combined	-0.115	-0.164	-0.066	-4.535	0.000
Ruiz et al. 2017	Combined	-0.117	-0.167	-0.067	-4.555	0.000
Ruiz et al. 2019	Combined	-0.118	-0.167	-0.068	-4.627	0.000
Santos-Rosa et al. 2022	Ego-involving 1	-0.117	-0.165	-0.068	-4.691	0.000
Scott et al. 2021	Combined	-0.115	-0.163	-0.066	-4.626	0.000
Smith, Balaguer et al. 2006	Combined	-0.117	-0.165	-0.068	-4.645	0.000
Solstad & Lemyre 2014	Performance Climate-1	-0.114	-0.162	-0.065	-4.584	0.000
Stark & Newton 2014	Ego Climate 1	-0.115	-0.163	-0.067	-4.661	0.000
Tamminen et al. 2016	Ego Climate 1	-0.117	-0.166	-0.069	-4.699	0.000
Vazou et al. 2006	Combined	-0.114	-0.163	-0.065	-4.545	0.000
Weiss 2015	Performance Climate-1	-0.113	-0.161	-0.065	-4.549	0.000
Weiss et al. 2009	Performance Climate-1	-0.113	-0.161	-0.065	-4.580	0.000
Zanatta et al. 2018	Ego-involving climate-1	-0.118	-0.166	-0.069	-4.685	0.000
Pooled		-0.115	-0.162	-0.068	-4.718	0.000

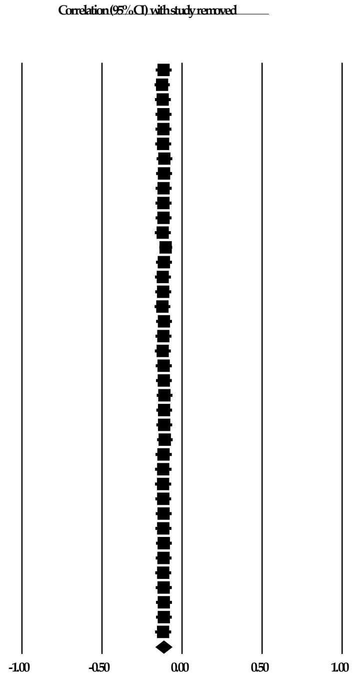

Supp Figure 4. Remove-one study results for ego/performance climate and positive affect/mood.

Meta Analysis

Study name	Subgroup within study	Statistics with study removed: Point	Lower limit	Upper limit	Z-Value	p-Value
Al-Yaaribi & Kavussanu 2018	Performance climate 1	-0.114	-0.161	-0.066	-4.664	0.000
Atkins et al. 2013	Combined	-0.123	-0.168	-0.078	-5.330	0.000
Boixadós et al. 2004	Ego Climate 2	-0.119	-0.166	-0.071	-4.890	0.000
Bortoli et al. 2009	Performance climate 1	-0.115	-0.162	-0.067	-4.697	0.000
Bortoli et al. 2011	Ego Climate 1	-0.115	-0.162	-0.068	-4.723	0.000
Bortoli et al. 2012	Performance climate 1	-0.117	-0.164	-0.069	-4.794	0.000
Cecchini et al. 2005	Performance climate 1	-0.109	-0.156	-0.063	-4.554	0.000
Cumming et al. 2007	Ego Climate 1	-0.111	-0.158	-0.064	-4.599	0.000
Curran et al. 2015	Combined	-0.114	-0.162	-0.066	-4.663	0.000
Dorsch et al. 2016	Combined	-0.115	-0.162	-0.067	-4.703	0.000
Draugelis et al. 2014	Combined	-0.114	-0.162	-0.067	-4.687	0.000
Eys et al. 2013	Combined	-0.119	-0.166	-0.072	-4.903	0.000
Gjesdal et al. 2018	Performance Climate 1	-0.101	-0.139	-0.063	-5.206	0.000
Guzmán & García 2014	Performance Climate 3	-0.112	-0.159	-0.065	-4.617	0.000
Habeeb et al. 2023	Combined	-0.119	-0.166	-0.071	-4.854	0.000
Harwood et al. 2019	Combined	-0.117	-0.164	-0.069	-4.784	0.000
Jaakkola et al. 2015	Ego-involving climate 1	-0.121	-0.168	-0.075	-5.062	0.000
Kipp & Weiss 2013	Performance Climate 6	-0.112	-0.159	-0.065	-4.610	0.000
Lemyre et al. 2008	Performance Climate 2	-0.115	-0.162	-0.068	-4.754	0.000
MacDonald et al. 2011	Combined	-0.119	-0.166	-0.072	-4.888	0.000
Monteiro et al. 2018	Ego-involving 2	-0.114	-0.161	-0.066	-4.621	0.000
Morales-Belando et al. 2021	Ego Climate 1	-0.113	-0.159	-0.065	-4.654	0.000
Newton et al. 2000	Ego Climate 1	-0.109	-0.155	-0.062	-4.550	0.000
Quested & Duda 2009	Ego climate 5	-0.111	-0.157	-0.064	-4.605	0.000
Quested & Duda 2010	Ego-involving 4	-0.111	-0.157	-0.064	-4.579	0.000
Robazza et al. 2021	Ego Climate 1	-0.107	-0.153	-0.061	-4.515	0.000
Robazza et al. 2022	Combined	-0.114	-0.162	-0.066	-4.598	0.000
Ruiz et al. 2017	Combined	-0.116	-0.165	-0.067	-4.618	0.000
Ruiz et al. 2019	Combined	-0.117	-0.165	-0.068	-4.690	0.000
Santos-Rosa et al. 2022	Ego-involving 1	-0.116	-0.163	-0.068	-4.753	0.000
Sari & Bizan 2022	Combined	-0.115	-0.162	-0.068	-4.718	0.000
Scott et al. 2021	Combined	-0.114	-0.161	-0.067	-4.689	0.000
Smith, Balaguer et al. 2006	Combined	-0.116	-0.163	-0.068	-4.708	0.000
Solstad & Lemyre 2014	Performance Climate 1	-0.113	-0.160	-0.065	-4.648	0.000
Stark & Newton 2014	Ego Climate 1	-0.114	-0.161	-0.067	-4.724	0.000
Tamminen et al. 2016	Ego Climate 1	-0.116	-0.164	-0.069	-4.761	0.000
Vazou et al. 2006	Combined	-0.114	-0.161	-0.065	-4.609	0.000
Weiss 2015	Performance Climate 1	-0.112	-0.159	-0.065	-4.613	0.000
Weiss et al. 2009	Performance Climate 1	-0.113	-0.159	-0.065	-4.644	0.000
Zanatta et al. 2018	Ego-involving climate 1	-0.117	-0.164	-0.069	-4.748	0.000
Pooled		-0.114	-0.161	-0.068	-4.781	0.000
Prediction Interval		-0.114	-0.384	0.173	0.000	0.000

Correlation (95% CI) with study removed

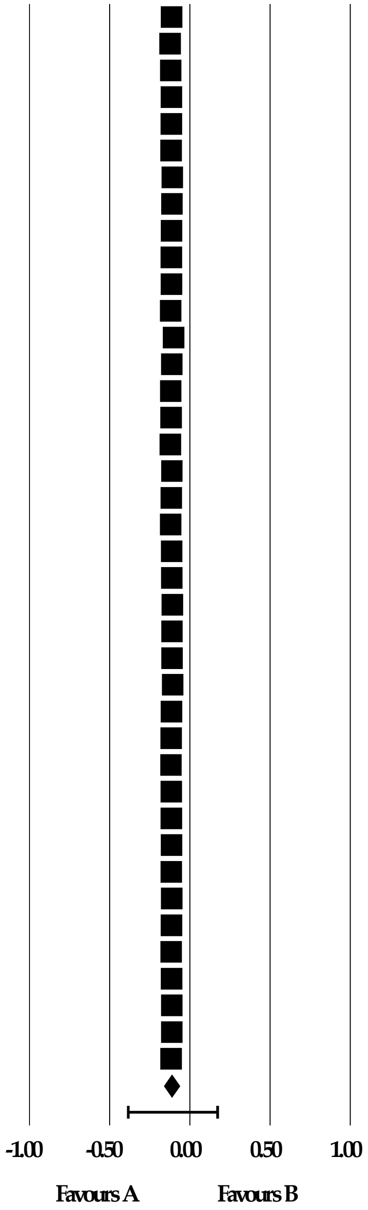

Supp Figure 5. Remove-one study results for ego/performance climate and negative affect/mood.

Study name	Subgroup within study	Statistics with study removed				
		Point	Lower limit	Upper limit	Z-Value	p-Value
Alferman et al. 2013 s1	Performance Climate 1	-0.190	-0.334	-0.038	-2.435	0.015
Alferman et al. 2013 s2	Performance Climate 1	-0.202	-0.345	-0.050	-2.598	0.009
Balaguer et al. 1999	Combined	-0.184	-0.336	-0.022	-2.227	0.026
Balaguer et al. 2002	Ego Climate 1	-0.199	-0.343	-0.046	-2.547	0.011
Bekiari & Syrmpas 2015	Performance climate 1	-0.114	-0.184	-0.042	-3.116	0.002
Blecharz et al. 2014 s2	Combined	-0.188	-0.335	-0.033	-2.367	0.018
Boixadós et al. 2004	Ego Climate 1	-0.188	-0.337	-0.029	-2.316	0.021
Bono & Livi 2016	Ego Climate 1	-0.186	-0.331	-0.032	-2.363	0.018
Calvo & Topa 2019	Ego-oriented climate 1	-0.190	-0.335	-0.036	-2.408	0.016
García-Calvo et al. 2014	Combined	-0.186	-0.343	-0.018	-2.169	0.030
Gillham et al. 2013	Combined	-0.183	-0.337	-0.020	-2.194	0.028
Guzmán & García 2014	Performance Climate 2	-0.187	-0.335	-0.030	-2.331	0.020
Haugen et al. 2020	Performance climate 1	-0.175	-0.326	-0.016	-2.152	0.031
Holgado et al. 2010	Ego-involving 1	-0.206	-0.345	-0.058	-2.711	0.007
Newton et al. 2000	Ego Climate 3	-0.181	-0.331	-0.023	-2.243	0.025
Tribojevic Jocic et al. 2020	Combined	-0.178	-0.325	-0.022	-2.228	0.026
Trenz & Zusho 2011	Performance climate 1	-0.171	-0.317	-0.017	-2.170	0.030
Walling et al. 1993	Performance Climate 2	-0.178	-0.325	-0.023	-2.244	0.025
Pooled		-0.183	-0.323	-0.034	-2.405	0.016
Prediction Interval		-0.183	-0.705	0.469	0.000	0.000

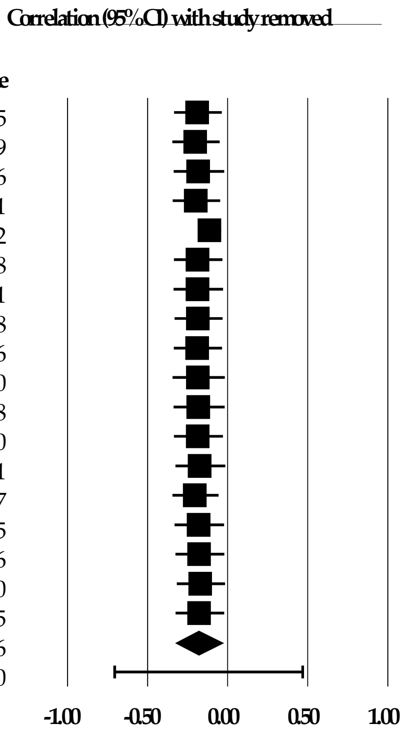

Supp Figure 6. Remove-one study results for ego/performance climate and satisfaction.

Supplement Figures. Cumulative analysis by year figures.

Meta Analysis

Subgroup within study	Study name	Cumulative statistics				
		Point	Lower limit	Upper limit	Z-Value	p-Value
Task Climate 1	Newton et al. 2000	0.520	0.443	0.589	11.264	0.000
Task Climate 2	Boixadós et al. 2004	0.339	-0.083	0.658	1.584	0.113
Task Climate 1	Cecchini et al. 2005	0.380	0.070	0.623	2.379	0.017
Combined	Smith, Balaguer et al. 2006	0.351	0.161	0.516	3.515	0.000
Combined	Vazou et al. 2006	0.348	0.218	0.466	5.028	0.000
Task Climate 1	Cumming et al. 2007	0.356	0.245	0.458	5.976	0.000
Mastery Climate 2	Lemyre et al. 2008	0.334	0.229	0.431	5.970	0.000
Mastery climate 1	Bortoli et al. 2009	0.321	0.228	0.408	6.486	0.000
Task climate 5	Quested & Duda 2009	0.333	0.244	0.416	6.989	0.000
Combined	Vosloo et al. 2009	0.326	0.245	0.402	7.558	0.000
Mastery Climate 1	Weiss et al. 2009	0.317	0.241	0.390	7.786	0.000
Task-involving 4	Quested & Duda 2010	0.326	0.255	0.393	8.555	0.000
Mastery climate 1	Bortoli et al. 2011	0.316	0.249	0.381	8.723	0.000
Combined	MacDonald et al. 2011	0.312	0.257	0.365	10.501	0.000
Combined	Núñez et al. 2011	0.308	0.258	0.356	11.584	0.000
Mastery climate 1	Bortoli et al. 2012	0.303	0.256	0.349	11.988	0.000
Combined	Atkins et al. 2013	0.295	0.248	0.340	11.756	0.000
Combined	Eys et al. 2013	0.289	0.246	0.331	12.484	0.000
Mastery Climate 6	Kipp & Weiss 2013	0.286	0.244	0.327	12.840	0.000
Combined	Draugelis et al. 2014	0.286	0.246	0.325	13.427	0.000
Mastery Climate 3	Guzmán & García 2014	0.280	0.241	0.319	13.314	0.000
Mastery Climate 1	Solstad & Lemyre 2014	0.285	0.246	0.323	13.712	0.000
Task Climate 1	Stark & Newton 2014	0.291	0.252	0.329	13.937	0.000
Combined	Atkins et al. 2015 s1	0.286	0.251	0.321	15.137	0.000
Combined	Atkins et al. 2015 s2	0.289	0.255	0.321	16.096	0.000
Combined	Curran et al. 2015	0.304	0.265	0.341	14.787	0.000
Task-involving climate 1	Jaakkola et al. 2015	0.313	0.274	0.351	14.841	0.000
Mastery Climate 1	Weiss 2015	0.316	0.278	0.353	15.311	0.000
Combined	Dorsch et al. 2016	0.313	0.276	0.349	15.572	0.000
Task Climate 1	Tamminen et al. 2016	0.321	0.283	0.358	15.597	0.000
Combined	Ruiz et al. 2017	0.313	0.271	0.354	13.789	0.000
Mastery climate 1	Al-Yaaribi & Kavussanu 2018	0.316	0.274	0.356	14.143	0.000
Mastery Climate 1	Gjesdal et al. 2018	0.327	0.280	0.374	12.672	0.000
Task-involving 2	Monteiro et al. 2018	0.322	0.275	0.368	12.678	0.000
Task-involving climate 1	Zanatta et al. 2018	0.324	0.279	0.369	13.067	0.000
Combined	Harwood et al. 2019	0.322	0.277	0.366	13.291	0.000
Combined	Ruiz et al. 2019	0.316	0.271	0.360	12.908	0.000
Task Climate 1	Morales-Belando et al. 2021	0.312	0.267	0.356	12.832	0.000
Mastery Climate 1	Robazza et al. 2021	0.314	0.270	0.358	13.128	0.000
Combined	Scott et al. 2021	0.312	0.268	0.355	13.231	0.000
Task climate 2	Trbojevic Jocic & Petrovic 2021	0.310	0.267	0.352	13.343	0.000
Combined	Robazza et al. 2022	0.308	0.267	0.349	13.750	0.000
Task-involving 1	Santos-Rosa et al. 2022	0.307	0.266	0.347	13.915	0.000
Combined	Sari & Bizan 2022	0.309	0.269	0.349	14.205	0.000
Task-involving climate 1	Amaro et al. 2023	0.315	0.275	0.354	14.480	0.000
Combined	Habeeb et al. 2023	0.314	0.275	0.353	14.810	0.000
	Pooled	0.314	0.275	0.353	14.810	0.000
	Prediction Interval	0.314	0.043	0.543	0.000	0.000

Cumulative correlation (95% CI)

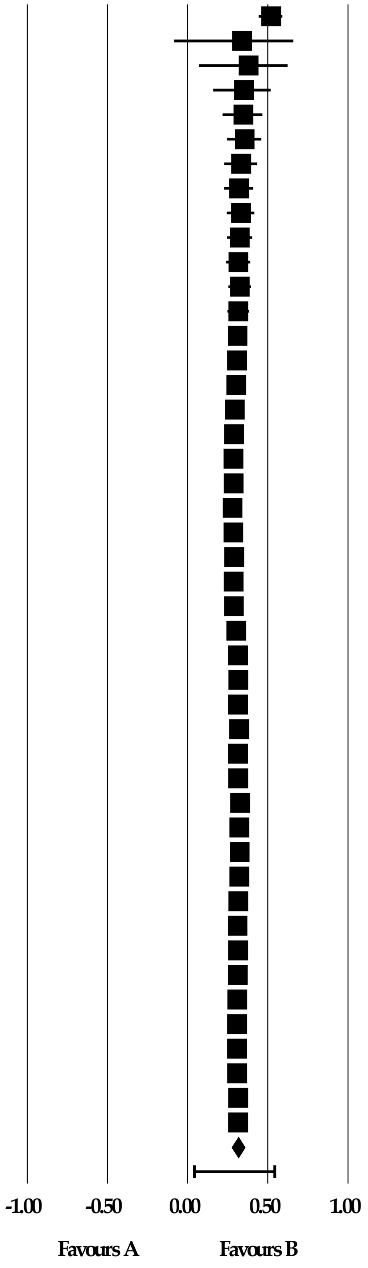

Supp Figure 8. Cumulative analysis by year for task/mastery climate and positive affect/mood.

Subgroup within study	Study name	Cumulative statistics: Point	Lower limit	Upper limit	Z-Value	p-Value
Mastery Climate 1	Walling et al. 1993	-0.180	-0.322	-0.030	-2.345	0.019
Combined	Ntoumanis & Biddle 1998	-0.108	-0.227	0.014	-1.734	0.083
Task Climate 2	Newton et al. 2000	-0.160	-0.275	-0.041	-2.634	0.008
Mastery climate 1	Pensgaard & Roberts 2000	-0.138	-0.245	-0.026	-2.421	0.015
Combined	Carr & Wyon 2003	-0.137	-0.213	-0.059	-3.433	0.001
Task Climate 2	Cecchini et al. 2005	-0.136	-0.203	-0.068	-3.903	0.000
Combined	Smith, Smoll et al. 2006	-0.163	-0.252	-0.071	-3.464	0.001
Combined	Vazou et al. 2006	-0.143	-0.236	-0.047	-2.901	0.004
Combined	Abrahamsen et al. 2008a	-0.133	-0.219	-0.045	-2.958	0.003
Combined	Abrahamsen et al. 2008b	-0.128	-0.207	-0.047	-3.102	0.002
Mastery climate 2	Bortoli et al. 2009	-0.131	-0.202	-0.059	-3.546	0.000
Task climate 6	Quested & Duda 2009	-0.148	-0.219	-0.075	-3.968	0.000
Combined	Steffen et al. 2009	-0.134	-0.213	-0.054	-3.260	0.001
Task-involving 5	Quested & Duda 2010	-0.147	-0.224	-0.068	-3.638	0.000
Mastery climate 1	Baric 2011	-0.126	-0.203	-0.047	-3.115	0.002
Combined	Garcia-Mas et al. 2011	-0.123	-0.198	-0.047	-3.176	0.001
Combined	O'Rourke et al. 2011	-0.130	-0.201	-0.058	-3.507	0.000
Mastery climate 2	Bortoli et al. 2012	-0.136	-0.205	-0.066	-3.805	0.000
Combined	Nordin-Bates et al. 2012	-0.131	-0.196	-0.065	-3.888	0.000
Combined	Draugelis et al. 2014	-0.128	-0.190	-0.066	-3.998	0.000
Combined	Mora et al. 2014 s1	-0.128	-0.189	-0.067	-4.109	0.000
Combined	Mora et al. 2014 s2	-0.137	-0.196	-0.076	-4.417	0.000
Combined	O'Rourke et al. 2014	-0.140	-0.197	-0.081	-4.671	0.000
Task Climate 2	Stark & Newton 2014	-0.143	-0.199	-0.086	-4.864	0.000
Mastery climate 1	Abrahamsen & Kristiansen 2015	-0.130	-0.188	-0.071	-4.304	0.000
Combined	Pineda-Espejel et al. 2015	-0.127	-0.183	-0.071	-4.393	0.000
Task Climate 2	Bono & Livi 2016	-0.130	-0.185	-0.074	-4.559	0.000
Combined	Dorsch et al. 2016	-0.126	-0.179	-0.072	-4.579	0.000
Combined	Ruiz et al. 2017	-0.123	-0.171	-0.074	-4.944	0.000
Mastery climate 2	Al-Yaaribi & Kavussanu 2018	-0.127	-0.174	-0.079	-5.193	0.000
Mastery Climate 2	Gjesdal et al. 2018	-0.130	-0.176	-0.084	-5.462	0.000
Combined	Sheehan et al. 2018a	-0.135	-0.180	-0.089	-5.721	0.000
Combined	Sheehan et al. 2018b	-0.136	-0.180	-0.091	-5.937	0.000
Combined	Ruiz et al. 2019	-0.137	-0.179	-0.094	-6.264	0.000
Combined	Gómez-López et al. 2020	-0.133	-0.174	-0.092	-6.302	0.000
Combined	Pineda-Espejel et al. 2021	-0.131	-0.171	-0.091	-6.345	0.000
Mastery Climate 2	Robazza et al. 2021	-0.121	-0.162	-0.080	-5.707	0.000
Combined	Wu et al. 2021	-0.130	-0.174	-0.084	-5.579	0.000
Combined	Robazza et al. 2022	-0.129	-0.172	-0.086	-5.824	0.000
Combined	Santos-Rosa et al. 2022	-0.127	-0.169	-0.085	-5.866	0.000
	Pooled	-0.127	-0.169	-0.085	-5.866	0.000
	Prediction Interval	-0.127	-0.365	0.127	0.000	0.000

Cumulative correlation (95% CI)

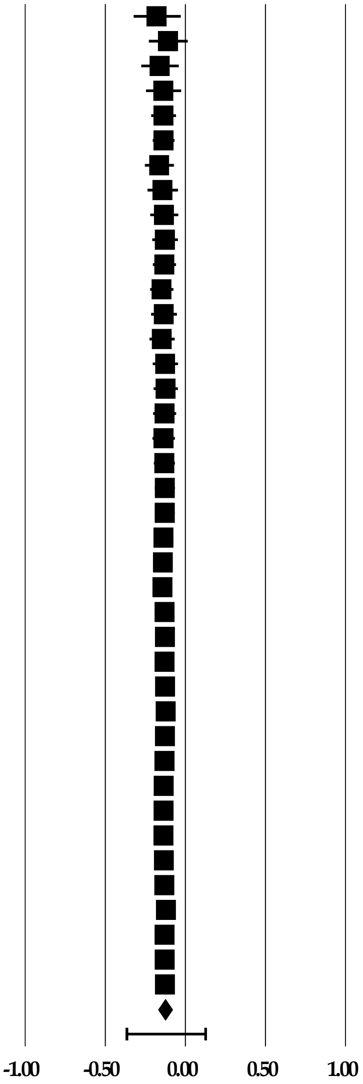

Supp Figure 9. Cumulative analysis by year for task/mastery climate and negative affect/mood.

Subgroup within study	Study name	Point	Lower limit	Upper limit	Z-Value	p-Value
Mastery Climate 2	Walling et al. 1993	0.390	0.254	0.511	5.306	0.000
Combined	Balaguer et al. 1999	0.324	0.231	0.411	6.545	0.000
Task Climate 3	Newton et al. 2000	0.358	0.272	0.437	7.718	0.000
Task Climate 1	Balaguer et al. 2002	0.330	0.244	0.411	7.144	0.000
Task Climate 1	Boixadós et al. 2004	0.382	0.250	0.500	5.373	0.000
Combined	Papaioannou et al. 2008	0.370	0.272	0.460	6.997	0.000
Task-involving 1	Holgado et al. 2010	0.344	0.250	0.431	6.846	0.000
Mastery climate 1	Trenz & Zusho 2011	0.373	0.280	0.459	7.349	0.000
Mastery Climate 1	Alferman et al. 2013 s1	0.354	0.262	0.440	7.133	0.000
Mastery Climate 1	Alferman et al. 2013 s2	0.354	0.268	0.434	7.621	0.000
Combined	Gillham et al. 2013	0.345	0.269	0.417	8.384	0.000
Task-involving 1	Blecharz et al. 2014 s1	0.361	0.286	0.432	8.782	0.000
Combined	Blecharz et al. 2014 s2	0.347	0.273	0.416	8.689	0.000
Combined	García-Calvo et al. 2014	0.329	0.251	0.403	7.865	0.000
Mastery Climate 2	Guzmán & García 2014	0.330	0.256	0.399	8.363	0.000
Mastery climate 1	Bekiari & Syrmpas 2015	0.382	0.275	0.481	6.523	0.000
Combined	Abraldes et al. 2016	0.372	0.269	0.467	6.650	0.000
Task Climate 1	Bono & Livi 2016	0.368	0.268	0.460	6.788	0.000
Task-oriented climate 1	Calvo & Topa 2019	0.364	0.268	0.453	6.977	0.000
Mastery climate 1	Haugen et al. 2020	0.359	0.268	0.444	7.300	0.000
Task climate 1	Tribojevic Jocic et al. 2020	0.372	0.283	0.455	7.683	0.000
	Pooled	0.372	0.283	0.455	7.683	0.000
	Prediction Interval	0.372	-0.085	0.700	0.000	0.000

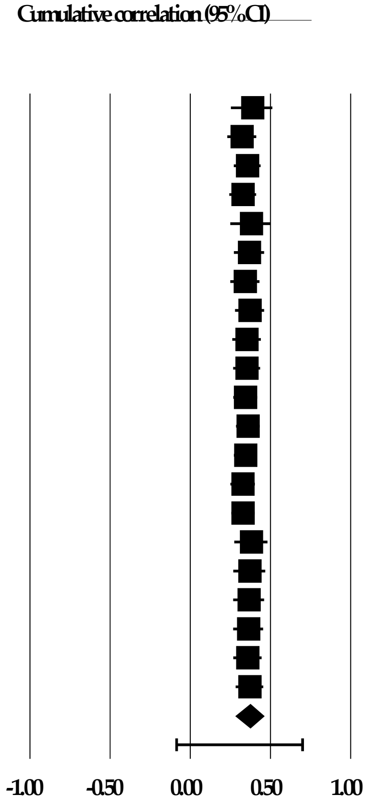

Supp Figure 10. Cumulative analysis by year for task/mastery climate and satisfaction.

Meta Analysis

Subgroup within study	Study name	Cumulative statistics				
		Point	Lower limit	Upper limit	Z-Value	p-Value
Ego Climate 1	Newton et al. 2000	-0.310	-0.398	-0.217	-6.265	0.000
Ego Climate 2	Boixadós et al. 2004	-0.134	-0.460	0.225	-0.726	0.468
Performance climate 1	Cecchini et al. 2005	-0.208	-0.464	0.080	-1.419	0.156
Combined	Smith, Balaguer et al. 2006	-0.167	-0.337	0.015	-1.801	0.072
Combined	Vazou et al. 2006	-0.158	-0.278	-0.032	-2.456	0.014
Ego Climate 1	Cumming et al. 2007	-0.169	-0.274	-0.059	-2.998	0.003
Performance Climate 2	Lemyre et al. 2008	-0.156	-0.253	-0.057	-3.062	0.002
Performance climate 1	Bortoli et al. 2009	-0.146	-0.230	-0.061	-3.352	0.001
Ego climate 5	Quested & Duda 2009	-0.158	-0.239	-0.075	-3.713	0.000
Performance Climate 1	Weiss et al. 2009	-0.161	-0.237	-0.084	-4.053	0.000
Ego-involving 4	Quested & Duda 2010	-0.170	-0.241	-0.098	-4.548	0.000
Ego Climate 1	Bortoli et al. 2011	-0.162	-0.228	-0.095	-4.679	0.000
Combined	MacDonald et al. 2011	-0.147	-0.223	-0.068	-3.655	0.000
Performance climate 1	Bortoli et al. 2012	-0.137	-0.209	-0.063	-3.636	0.000
Combined	Atkins et al. 2013	-0.113	-0.192	-0.033	-2.767	0.006
Combined	Eys et al. 2013	-0.100	-0.171	-0.028	-2.709	0.007
Performance Climate 6	Kipp & Weiss 2013	-0.107	-0.176	-0.036	-2.962	0.003
Combined	Draugelis et al. 2014	-0.107	-0.173	-0.039	-3.098	0.002
Performance Climate 3	Guzmán & García 2014	-0.112	-0.177	-0.046	-3.326	0.001
Performance Climate 1	Solstad & Lemyre 2014	-0.115	-0.178	-0.051	-3.512	0.000
Ego Climate 1	Stark & Newton 2014	-0.115	-0.177	-0.053	-3.591	0.000
Combined	Curran et al. 2015	-0.115	-0.175	-0.055	-3.749	0.000
Ego-involving climate 1	Jaakkola et al. 2015	-0.103	-0.162	-0.044	-3.385	0.001
Performance Climate 1	Weiss 2015	-0.107	-0.165	-0.049	-3.592	0.000
Combined	Dorsch et al. 2016	-0.106	-0.162	-0.050	-3.700	0.000
Ego Climate 1	Tamminen et al. 2016	-0.103	-0.157	-0.049	-3.739	0.000
Combined	Ruiz et al. 2017	-0.100	-0.150	-0.051	-3.944	0.000
Performance climate 1	Al-Yaaribi & Kavussanu 2018	-0.102	-0.150	-0.053	-4.100	0.000
Performance Climate 1	Gjesdal et al. 2018	-0.119	-0.180	-0.057	-3.758	0.000
Ego-involving 2	Monteiro et al. 2018	-0.120	-0.179	-0.060	-3.933	0.000
Ego-involving climate 1	Zanatta et al. 2018	-0.117	-0.173	-0.059	-3.972	0.000
Combined	Harwood et al. 2019	-0.114	-0.169	-0.058	-3.971	0.000
Combined	Ruiz et al. 2019	-0.110	-0.163	-0.057	-4.069	0.000
Ego Climate 1	Morales-Belando et al. 2021	-0.113	-0.164	-0.060	-4.206	0.000
Ego Climate 1	Robazza et al. 2021	-0.121	-0.173	-0.068	-4.482	0.000
Combined	Scott et al. 2021	-0.121	-0.172	-0.070	-4.573	0.000
Combined	Robazza et al. 2022	-0.121	-0.170	-0.072	-4.764	0.000
Ego-involving 1	Santos-Rosa et al. 2022	-0.120	-0.168	-0.071	-4.791	0.000
Combined	Sari & Bizan 2022	-0.119	-0.166	-0.071	-4.854	0.000
Combined	Habeeb et al. 2023	-0.114	-0.161	-0.068	-4.781	0.000
	Pooled	-0.114	-0.161	-0.068	-4.781	0.000
	Prediction Interval	-0.114	-0.384	0.173	0.000	0.000

Cumulative correlation (95% CI)

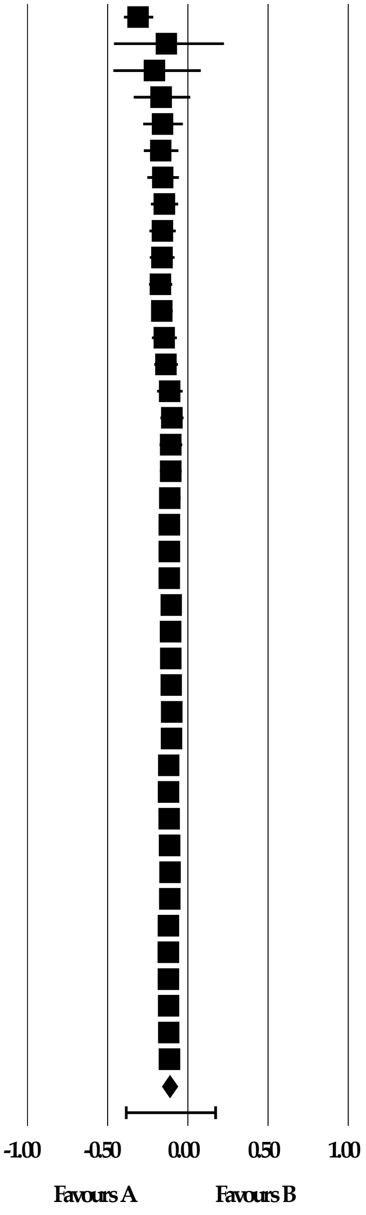

Supp Figure 11. Cumulative analysis by year for ego/performance climate and positive affect/mood.

Subgroup within study	Study name	Cumulative statistics				
		Point	Lower limit	Upper limit	Z-Value	p-Value
Performance Climate 1	Walling et al. 1993	0.390	0.254	0.511	5.306	0.000
Combined	Ntoumanis & Biddle 1998	0.181	-0.254	0.555	0.811	0.418
Ego Climate 2	Newton et al. 2000	0.258	-0.047	0.519	1.664	0.096
Ego Climate 1	Pensgaard & Roberts 2000	0.208	-0.052	0.442	1.576	0.115
Combined	Carr & Wyon 2003	0.216	0.039	0.380	2.380	0.017
Performance climate 2	Cecchini et al. 2005	0.252	0.090	0.400	3.028	0.002
Combined	Smith, Smoll et al. 2006	0.262	0.147	0.369	4.391	0.000
Combined	Vazou et al. 2006	0.250	0.154	0.342	4.974	0.000
Combined	Abrahamsen et al. 2008a	0.246	0.161	0.326	5.581	0.000
Combined	Abrahamsen et al. 2008b	0.231	0.150	0.308	5.473	0.000
Performance climate 2	Bortoli et al. 2009	0.229	0.156	0.300	6.037	0.000
Ego climate 6	Quested & Duda 2009	0.236	0.165	0.304	6.400	0.000
Combined	Steffen et al. 2009	0.237	0.182	0.292	8.129	0.000
Ego-involving 5	Quested & Duda 2010	0.245	0.192	0.296	8.833	0.000
Performance climate 1	Baric 2011	0.220	0.156	0.283	6.543	0.000
Combined	Garcia-Mas et al. 2011	0.217	0.155	0.278	6.687	0.000
Combined	O'Rourke et al. 2011	0.223	0.165	0.279	7.383	0.000
Performance climate 2	Bortoli et al. 2012	0.221	0.166	0.275	7.703	0.000
Combined	Nordin-Bates et al. 2012	0.221	0.169	0.272	8.186	0.000
Combined	Draugelis et al. 2014	0.216	0.166	0.265	8.228	0.000
Combined	Mora et al. 2014 s1	0.216	0.167	0.264	8.417	0.000
Combined	Mora et al. 2014 s2	0.216	0.168	0.263	8.607	0.000
Combined	O'Rourke et al. 2014	0.216	0.170	0.261	8.987	0.000
Ego Climate 2	Stark & Newton 2014	0.222	0.177	0.267	9.308	0.000
Combined	Pineda-Espejel et al. 2015	0.218	0.174	0.262	9.384	0.000
Ego Climate 2	Bono & Livi 2016	0.217	0.173	0.260	9.503	0.000
Combined	Dorsch et al. 2016	0.210	0.165	0.254	9.013	0.000
Combined	Ruiz et al. 2017	0.205	0.159	0.250	8.532	0.000
Performance climate 2	Al-Yaaribi & Kavussanu 2018	0.203	0.158	0.247	8.724	0.000
Performance Climate 2	Gjesdal et al. 2018	0.211	0.165	0.256	8.793	0.000
Combined	Sheehan et al. 2018a	0.217	0.172	0.262	9.131	0.000
Combined	Sheehan et al. 2018b	0.215	0.171	0.258	9.295	0.000
Combined	Ruiz et al. 2019	0.213	0.170	0.254	9.638	0.000
Combined	Gómez-López et al. 2020	0.205	0.161	0.248	8.993	0.000
Ego Climate 2	Robazza et al. 2021	0.195	0.150	0.239	8.281	0.000
Combined	Wu et al. 2021	0.198	0.154	0.242	8.637	0.000
Combined	Robazza et al. 2022	0.198	0.156	0.239	9.037	0.000
Combined	Santos-Rosa et al. 2022	0.198	0.157	0.238	9.265	0.000
	Pooled	0.198	0.157	0.238	9.265	0.000
	Prediction Interval	0.198	-0.048	0.421	0.000	0.000

Cumulative correlation (95% CI)

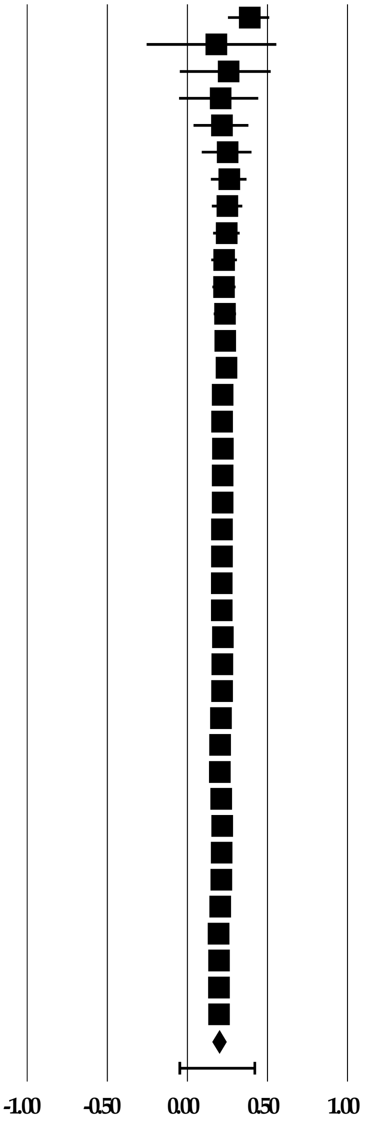

Supp Figure 12. Cumulative analysis by year for ego/performance climate and negative affect/mood.

Subgroup within study	Study name	Cumulative statistics				
		Point	Lower limit	Upper limit	Z-Value	p-Value
Performance Climate 2	Walling et al. 1993	-0.260	-0.395	-0.113	-3.429	0.001
Combined	Balaguer et al. 1999	-0.188	-0.283	-0.089	-3.699	0.000
Ego Climate 3	Newton et al. 2000	-0.184	-0.238	-0.129	-6.429	0.000
Ego Climate 1	Balaguer et al. 2002	-0.132	-0.255	-0.004	-2.021	0.043
Ego Climate 1	Boixadós et al. 2004	-0.124	-0.218	-0.027	-2.499	0.012
Ego-involving 1	Holgado et al. 2010	-0.065	-0.209	0.081	-0.873	0.383
Performance climate 1	Trenz & Zusho 2011	-0.108	-0.248	0.037	-1.462	0.144
Performance Climate 1	Alferman et al. 2013 s1	-0.100	-0.232	0.035	-1.457	0.145
Performance Climate 1	Alferman et al. 2013 s2	-0.073	-0.199	0.057	-1.102	0.271
Combined	Gillham et al. 2013	-0.083	-0.192	0.028	-1.464	0.143
Combined	Blecharz et al. 2014 s2	-0.083	-0.183	0.019	-1.602	0.109
Combined	García-Calvo et al. 2014	-0.087	-0.172	-0.001	-1.987	0.047
Performance Climate 2	Guzmán & García 2014	-0.089	-0.168	-0.009	-2.193	0.028
Performance climate 1	Bekiari & Syrmpas 2015	-0.181	-0.353	0.003	-1.928	0.054
Ego Climate 1	Bono & Livi 2016	-0.177	-0.342	-0.001	-1.973	0.048
Ego-oriented climate 1	Calvo & Topa 2019	-0.169	-0.328	-0.001	-1.975	0.048
Performance climate 1	Haugen et al. 2020	-0.178	-0.325	-0.022	-2.228	0.026
Combined	Tribojevic Jocic et al. 2020	-0.183	-0.323	-0.034	-2.405	0.016
	Pooled	-0.183	-0.323	-0.034	-2.405	0.016
	Prediction Interval	-0.183	-0.705	0.469	0.000	0.000

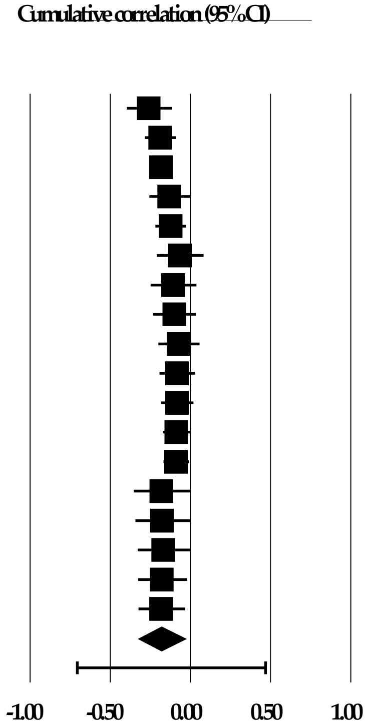

Supp Figure 13. Cumulative analysis by year for ego/performance climate and satisfaction.
